# Synthesis and preliminary assessment of fluorescent probes based on the competitive GPCR antagonists vismodegib and masupirdine

**DOI:** 10.1039/d6ra01214k

**Published:** 2026-04-01

**Authors:** Carlson Alexander, Tsz-Lam Cheung, Chowan Ashok Kumar, Catherine H. H. Hor, Huishan Li, Xiuzhi Zou, David Parker

**Affiliations:** a Department of Chemistry, Hong Kong Baptist University Kowloon Tong Hong Kong SAR China davidparker@hkbu.edu.hk

## Abstract

A series of six new fluorescent probes has been synthesised based on vismodegib, a leading antagonist for the G-protein coupled receptor smoothened, and the potent and selective 5-HT_6_ (serotonin) receptor antagonist, masupirdine, that bind with nanomolar affinities to the endogenous receptors. The X-ray structure of masupirdine has been determined at 100 K; it crystallises in the monoclinic system in space group *P*2_1_/*c*. Each compound in the series of red and green fluorescent dyes showed no significant toxicity when administered to NIH-3T3 and A549 cells at sub-micromolar concentrations. Two neutral naphthalimide conjugates of vismodegib, exhibited fast and non-specific cell uptake and showed no tendency to localise in the mitochondria or lysosomes, as determined by live cell imaging experiments using confocal microscopy. In contrast, a masupirdine–naphthalimide conjugate showed a rapid and pronounced staining of the lysosomes, at 25 nM probe concentrations within 15 minutes. The anionic conjugates based on Alexa-532 and cyanine-5 fluorophores showed no cell uptake and bound more weakly to a non-specific protein, as required for their potential use as fluorescent probes that target the GPCRs.

## Abstract 2

Synthesis, cell viability and imaging of the first family of fluorescent conjugates (1,8-naphthalimide, cyanine-5, Alexa-532), based on vismodegib and masupirdine (competitive antagonists for GPCR) is reported.

## Graphical abstract

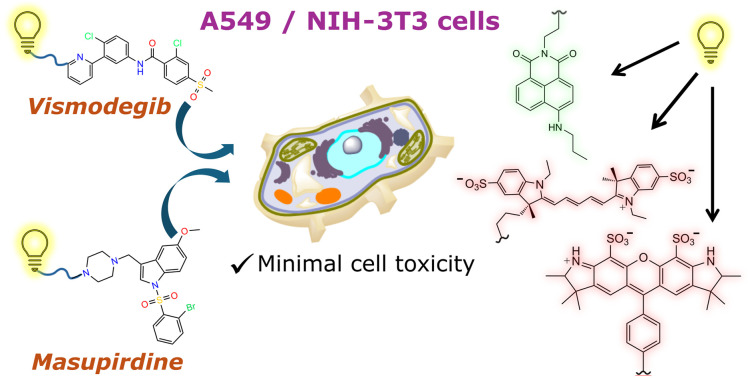

## Introduction

We report the synthesis and characterisation of the first family of fluorescent conjugates based on vismodegib and masupirdine. These compounds are well-established competitive antagonists for the G-protein coupled receptors, smoothened and human 5-HT_6_, that are of key importance in the hedgehog and serotonin signalling pathways.

In seeking to devise a new luminescent probe for the hedgehog signalling pathway, we were attracted by the idea of using vismodegib as the targeting moiety and resolved to conjugate it to a set of emissive dyes with complementary excitation and emission characteristics. Vismodegib is an orally administered inhibitor of the hedgehog signalling pathway, with an IC_50_ value of 3 nM. It was approved for use by the Food and Drug Administration in 2012, for the treatment of advanced basal-cell carcinoma and medulloblastoma.^1^ It clears only slowly from the body, allowing prolonged exposure. It has been shown to bind selectively to the key G-protein coupled receptor, smoothened, (SMO) that is essential for normal embryonic development and tissue homeostasis.^2^ This receptor is primarily found on the cell membrane, but importantly can also localise to primary cilia.^3^ Vismodegib functions as a competitive antagonist for the transmembrane protein SMO, inhibiting the activation and nuclear translocation of several factors involved in the hedgehog signalling pathway, and preventing the activation of downstream hedgehog target genes.^4^

**Fig. 1:**
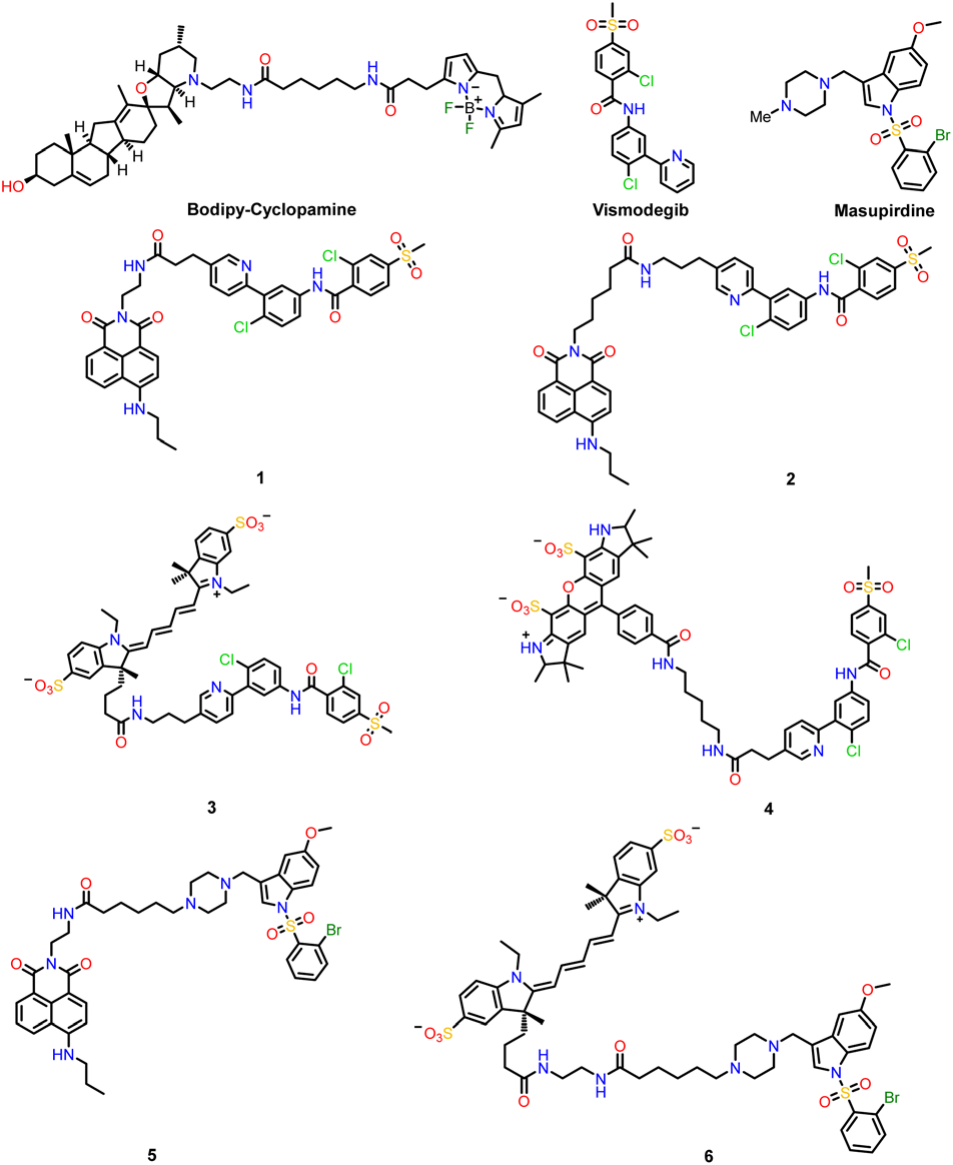
Structures of BODIPY cyclopamine, vismodegib, masupirdine and the fluorescent conjugates, 1–6, examined herein as cell staining probes.

Masupirdine (SUVN 502; Fig. 1 and Scheme S1)^5^ is a selective 5-HT_6_ (serotonin) receptor antagonist (*K*_i_ 2 nM, for the human 5-HT_6_ GPCR), with pro-cognitive properties in a range of mammalian cognition models. It is being considered for the treatment of agitation in Alzheimer's disease and has attracted clinical interest widely.^5*a*–*c*^ The piperazine ring has a p*K*_a_ of 8.4, and when protonated forms a crucial salt bridge with a conserved Asp residue in one of the 7 alpha-helical transmembrane domains (TM3). In parallel with the study of vismodegib conjugate probes, fluorescent conjugates of masupirdine have been prepared.

For over 20 years, BODIPY-cyclopamine has been used as a fluorescent probe conjugate for screening affinity of drugs to the smoothened receptor. The steroidal alkaloid, cyclopamine, is a plant-derived teratogen that specifically blocks cellular responses to hedgehog signalling.^6^ Its inhibitory effect is mediated by binding to the hepta-helical bundle of Smoothened (Smo). The probe retains moderate potency in smoothened hedgehog signalling inhibition (IC_50_ = 150 nM), targeting the cholesterol binding pocket.^7^ In the conjugate structure, a long spacer group (13 atoms) occurs between the BODIPY moiety and the steroid targeting group (Fig. 1).

### Structural modification criteria for antagonist linkage

**Fig. 2:**
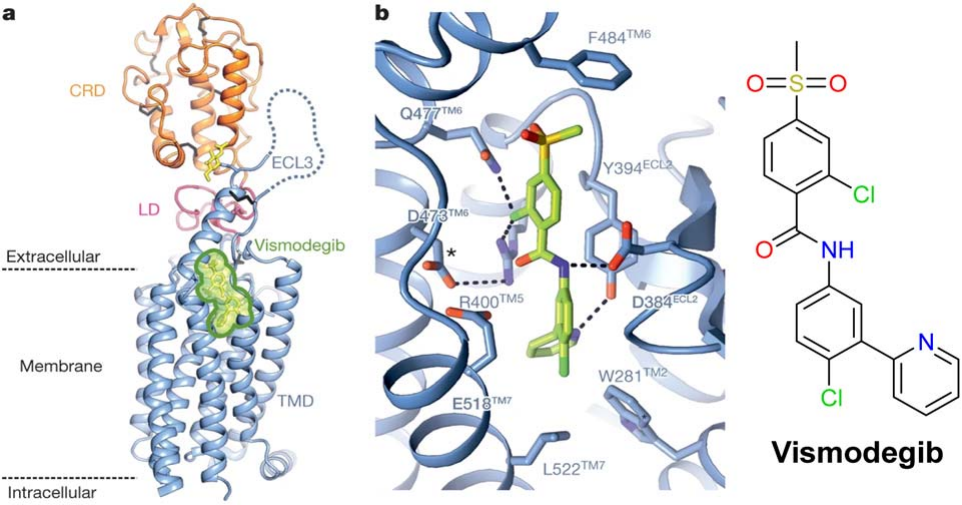
(a) Structure of the extracellular and transmembrane domains of human SMO in its vismodegib complex (adapted and reproduced with permission from reference 4a); (b) the local vismodegib binding site (the asterisk denotes the location of Asp 473, a residue mutated in vismodegib-resistant tumours).

A key design issue was to select a suitable linkage point on the antagonist structure for each system. The X-ray structure of smoothened bound to vismodegib has been reported to 3.3 Å resolution,^4^ revealing the presence of the hepta-helical transmembrane domain and an extracellular cysteine-rich domain. Vismodegib occupies the transmembrane site and the pyridine ring and 4-substituted chlorophenyl rings are deeply buried within it, forming hydrophobic interactions with the transmembrane domain core (Fig. 2). The chlorophenylmethylsulfone moiety is oriented towards the extracellular domains and the entrance to the hydrophobic binding site. This sulfone sub-structure is stabilised by strong hydrogen bonding to the side chains of Gln-477 and Arg-400 and there is a favourable hydrophobic stacking interaction between the phenylsulfonyl group and the aryl group in Phe-484. Asp-473 is a key residue stabilising the hydrogen bonding network around Arg-400 notably; in the SMO-Asp-473-His mutant, vismodegib binding is severely compromised.

Given these key structural features, the linkage position on vismodegib structure was reasoned to be best made to the pyridine ring meta-position, so that the fluorophore-containing substituent can be directed away from the key hydrogen bonding network, notwithstanding its location in a hydrophobic and potentially less accessible environment. The inclusion of a relatively nonpolar spacer chain was envisaged to be compatible with the hydrophobic nature of the local membrane environment.

With the masupirdine conjugates, a decision was taken to retain the molecular structure of the rigid aromatic entity and make a derivative by functionalising the piperazine ring while retaining its inherent basicity. Thus, the methyl substituent was transformed into a short pentamethylene chain bearing an acid group to allow amide coupling to an amine derivative of the well separated fluorophore.

### Fluorophore selection criteria

The series of fluorescent probes, 1–6, was designed based on vismodegib or masupirdine, with a view to examining their behaviour as acceptors in future FRET assays, as GPCR selective probes in over-expressed cell models and as simple cellular stains themselves. The fluorescent reporter groups chosen were selected to be either a charge-neutral 1,8 naphthalimide group^8^*i.e.*1, 2 and 5 with up to 10 atom spacers, (*λ*_ex_/*λ*_em_: 448/545 nm), or a more polar anionic cyanine-5 dye, 3 and 6,^9^ (*λ*_ex_/*λ*_em_: 640/670 nm) with eight and 13 atom spacers. The anionic Alexa-532 fluorophore^10^ (*λ*_ex_/*λ*_em_: 514/570 nm) was also prepared, linked *via* an eleven atom chain to vismodegib, in compound 4.

Naphthalimides are popular green emitting fluorophores because of their ease of synthesis and derivatisation and have been used both as both FRET donors^11,12^ and acceptors^13,14^ in a variety of sensing and biomolecule labelling examples. The yellow-emitting fluorophores based on Alexa-532 have also found innumerable uses as FRET labels in cell biology, notably in flow cytometry and for live cell imaging applications.^15,16^ The cyanine-5 dyes are the pre-eminent red-emitting fluorophores and are widely used as FRET acceptors and for live cell imaging and *in vivo* applications.^17,18^ The red-emitting cyanine-5 moiety has proved particularly useful in time-resolved FRET studies, as it can serve as an efficient acceptor to Eu emitting donors that possess long emission lifetimes.^19^

In summary, this small family of six dye-conjugates were designed to possess complementary excitation and emission wavelength characteristics (Fig. 1). It was reasoned that the overall negative charge of probes 3, 4 and 6 should tend to suppress non-specific protein binding and self-aggregation and therefore tend to inhibit non-specific cell uptake at low probe concentrations.^19–21^

## Results and discussion

### Synthesis and structural characterisation

**Fig. 3:**
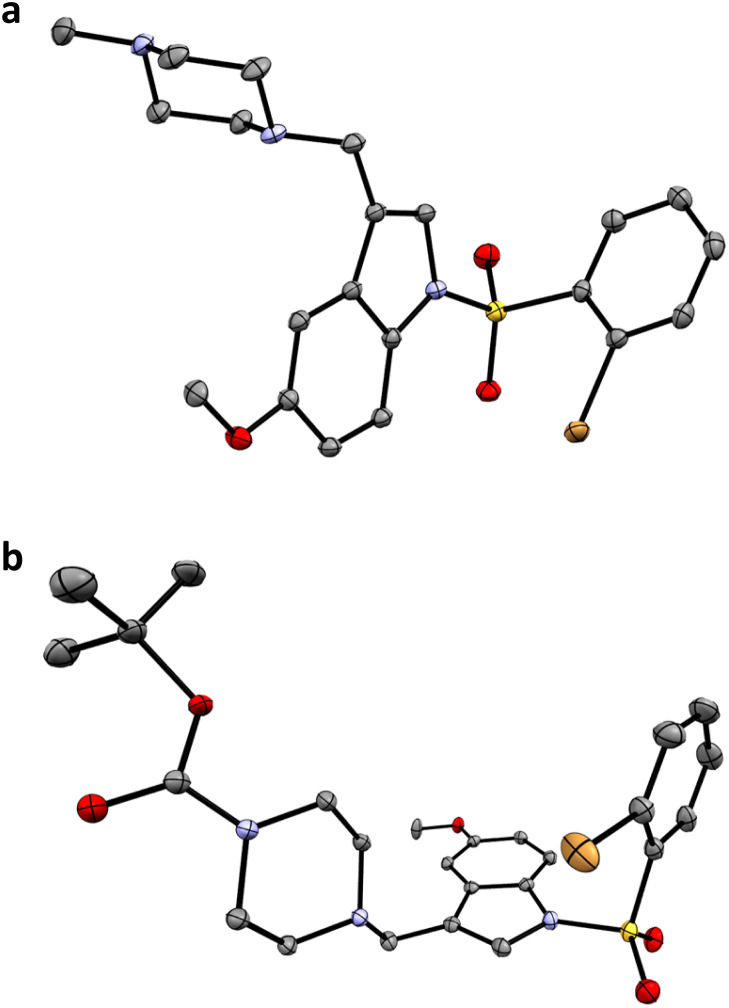
X-ray structures of masupirdine (a) and its 4′-piperazinyl-*N*-Boc derivative compound 12 in the SI, (b). Thermal ellipsoids are drawn at the 50% probability level; carbon in grey, nitrogen in purple, oxygen in red, sulphur in yellow, bromine in gold, bonds in black.

The synthesis of the probes followed standard methods with only minor adaptations made to classical literature procedures; full details are given in the SI, (Schemes S1–S5).^22,23^ The X-ray structures of masupirdine and its 4′-piperazinyl-*N*-Boc synthetic intermediate (CCDC 2529009 and 2529010) were determined at 100 K. Each structure highlights the relatively rigid molecular conformation around the sulphonamide group, and in each structure the piperazine ring adopts the chair conformation (Fig. 3 and Table S3). It is noteworthy that the structure of vismodegib has been determined previously, and in that case powder diffraction methods were used, for a sample measured at 295 K.^24^

### Photophysical characterisation and protein affinity determination

**Fig. 4:**
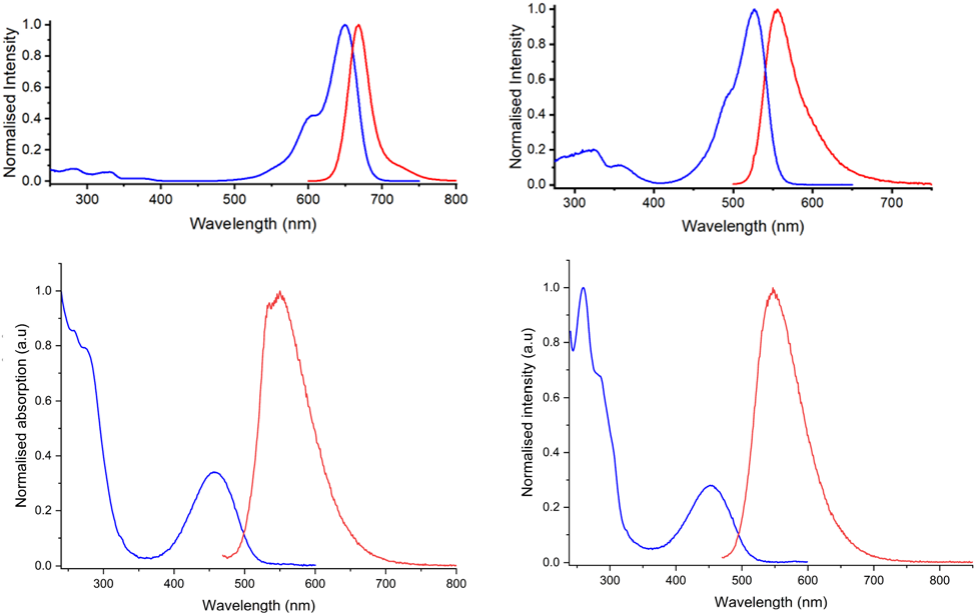
(Upper): absorption (blue) and emission (red, *λ*_ex_ 651 nm) spectra for 3 (left) and 4 (right, *λ*_ex_ 528 nm), (295 K, 10 µM, 0.01 M HEPES pH 7.4); (lower): normalised absorption (blue) and fluorescence emission spectra (red): 1 (left, *λ*_ex_ 454 nm), 5 (right, *λ*_ex_ 454 nm); (10 µM, 0.01 M HEPES pH 7.4, 295 K).

Absorption and emission spectral data (Fig. 4) for compounds 1–6 are collected in Table 1, together with their molecular weight data and bovine serum albumin protein binding constants. No significant pH dependence was found in the absorption and emission behaviour of each compound examined, (Fig. S31/32; S35/36; S38/39 SI). The extinction coefficient data measured were comparable to those reported for these commonly studied fluorophores and their simple derivatives.^9–18^

**Table 1:** Selected photophysical data for vismodegib, masupirdine and compounds 1–6 (pH 7.4, 50 mM HEPES, 50 mM NaCl, 295 K)^b^^,^^c^^,^^d^^,^^e^^,^^f^

Compound	MW	*λ*_abs_/nm	*λ*_em_/nm	ε/mM^−1^ cm^−1^	Log *K* (BSA)
Vismodegib	420	276	343	19.8	5.28 (±2.8%)
Masupirdine	478	298	348	10.4	6.18 (±4.3%)^14^
1	772	458	550	13.2	6.15 (±10%)
2	828	458	551	13.8	4.75 (±2.5%)
3	1102	651	669	160	a
4	1185	528	556	56	4.50 (±3%)
5	858	452	550	13.6	5.25 (±4.1%)
6	1244	650	669	131	a

a No significant spectral changes were observed following incremental addition of BSA.

b Log *K* values assume a dominant 1 : 1 binding stoichiometry.

c No pH dependence was observed in absorption/emission spectra between pH 4.5 and 8.0.

d Data for vismodegib were obtained in DMSO.

e ± Values refer to the standard error for the binding constants in log_10_ units, analysing the binding isotherms of three independent measurements plotted in DYNAFIT^®^, employing the NL2SOL algorithm, with a confidence interval at the 95% probability level.^26^

f Samples were dissolved in 80 : 20 water/methanol (1 mM, 3 mL) and were diluted to between 1 and 10 µM in 50 mM HEPES, 50 mM NaCl, pH 7.4.

**Fig. 5:**
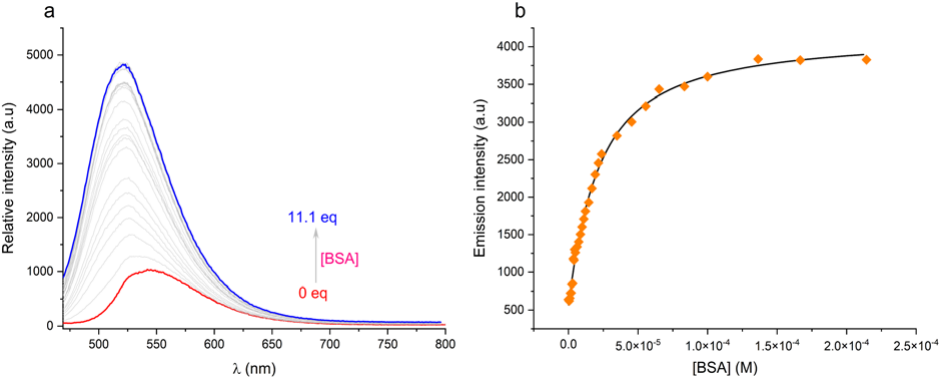
(a) Fluorescence titration of the naphthalimide derivative, 2 (1 µM) with bovine serum albumin (0.01 M HEPES buffer, pH 7.4, 295 K, *λ*_ex_ 454 nm, *λ*_em_ 542 nm). (b) Showing the fit to the experimental data points for a 1 : 1 binding stoichiometry; log *K* = 6.15 (±10%); see SI for other examples, statistical analysis and full data sets.

Bovine serum albumin (BSA)^25^ was selected as a model protein for binding studies because it is the most abundant protein present in the cell incubation medium used for the confocal microscopy imaging experiments, as described below and in the SI. The log *K* values were determined by non-linear, least squares iterative analysis and ranged from 4.50 for compound 4 to 6.15 for the neutral naphthalimide derivative, 1 (Fig. 5 and S30, S34, S42: see section 4 in SI for statistical analyses). Thus, as the concentrations used for cell staining are 1 µM or less, only compound 1 amongst these fluorescent probes will be significantly protein bound in the cell incubation medium, when present at concentrations around 1 µM. The other probes will typically be less than 10% protein bound, when added at the sub-micromolar concentrations that were used in the cell imaging experiments (*vide infra*).

### Preliminary cell imaging studies using confocal microscopy

The cellular distribution and uptake profiles of each compound were evaluated in a series of cell imaging experiments, using mouse skin fibroblasts (NIH-3T3) and human lung adenocarcinoma (A549) cells as models to assess non-specific cell staining in normal and cancer cells, respectively. A Nikon AXR confocal microscope was used in this work, equipped with seven laser excitation sources, including 445 nm for excitation of the naphthalimide compounds 1, 2 and 5, 514 nm for compound 4 and 640 nm for the cyanine-5 dye conjugates, 3 and 6.

Quantitative analyses were based on three independent replicates, performed on different days with separate cell passages. For each condition within a replicate, images were acquired from three random, non-overlapping fields of view. From these fields, a total of three representative cells were selected for co-localisation quantitative analysis, based on predefined criteria, resulting in a total of nine cells analysed per condition, across all replicates. Prior to the main experiments, the optimal imaging conditions were found. This process included varying the concentration of each compound to determine the lowest effective concentration that yielded a robust fluorescent signal. In parallel various incubation times were tested to establish the optimal protocol. Similarly, the best incubation conditions for organelle trackers (LysoBrite Deep Red and MitoTracker Deep Red) were empirically determined, following manufacturer guidelines as a starting point. For all quantitative imaging, confocal acquisition parameters, including laser power, detector gain, pinhole aperture, and pixel dwell time were held constant across each sample and replicate to ensure comparability.

**Fig. 6:**
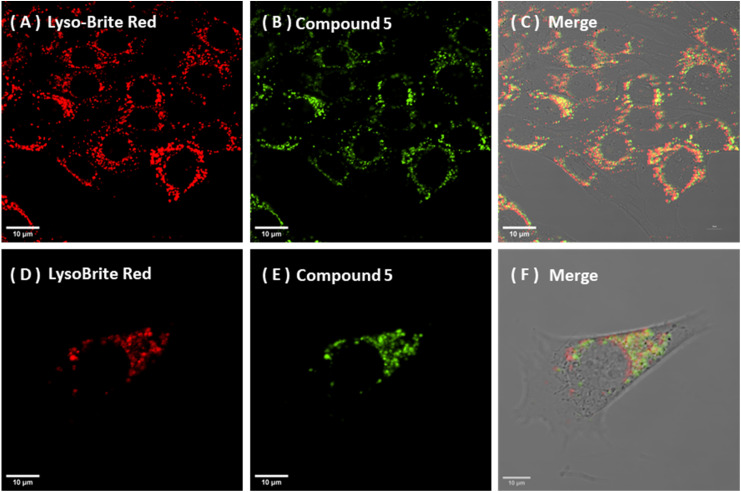
Confocal microscopy images showing time and concentration-dependent lysosomal staining of compound 5 in NIH-3T3 cells: (A–C): NIH-3T3, 25 nM 15 min: LysoBrite Deep Red (A, *λ*_ex_ 561 nm, *λ*_em_ 610–620 nm), Compound 5 channel (B, *λ*_ex_ 445 nm, *λ*_em_ 500–555 nm); merged image (C). (D–F): NIH-3T3, 50 nM 60 min: LysoBrite Deep Red (D), Compound 5 channel (E), merged image (F). The average Pearson's correlation coefficient was 0.82 ± 0.02 (mean/SD, *n* = 3). Scale bar = 10 µm.

Compound 5 showed a consistent and predominant lysosomal association in NIH-3T3 and A549 cells even at early time points, with comparable co-localisation patterns across each cell type. In NIH-3T3 cells, the intracellular distribution of compound 5 exhibited a clear time/concentration dependence. Dosing at 25 nM for 15 min represented the lowest fluorescent working concentration and proved to be the shortest incubation time that still produced reliable cellular uptake and lysosomal co-localisation profiles. Using 50 nM for 60 min yielded a stronger apparent lysosomal accumulation (Fig. 6 and 8). This conclusion is supported by ROI-based co-localisation analyses using Coloc2 with Costes automatic thresholding methods, giving a Pearson's *r* value (above the Costes threshold) of 0.82(0.02) in NIH-3T3 and 0.72(0.05) in A549 cells respectively, under common 50 nM/60 min incubation conditions.

**Fig. 7:**
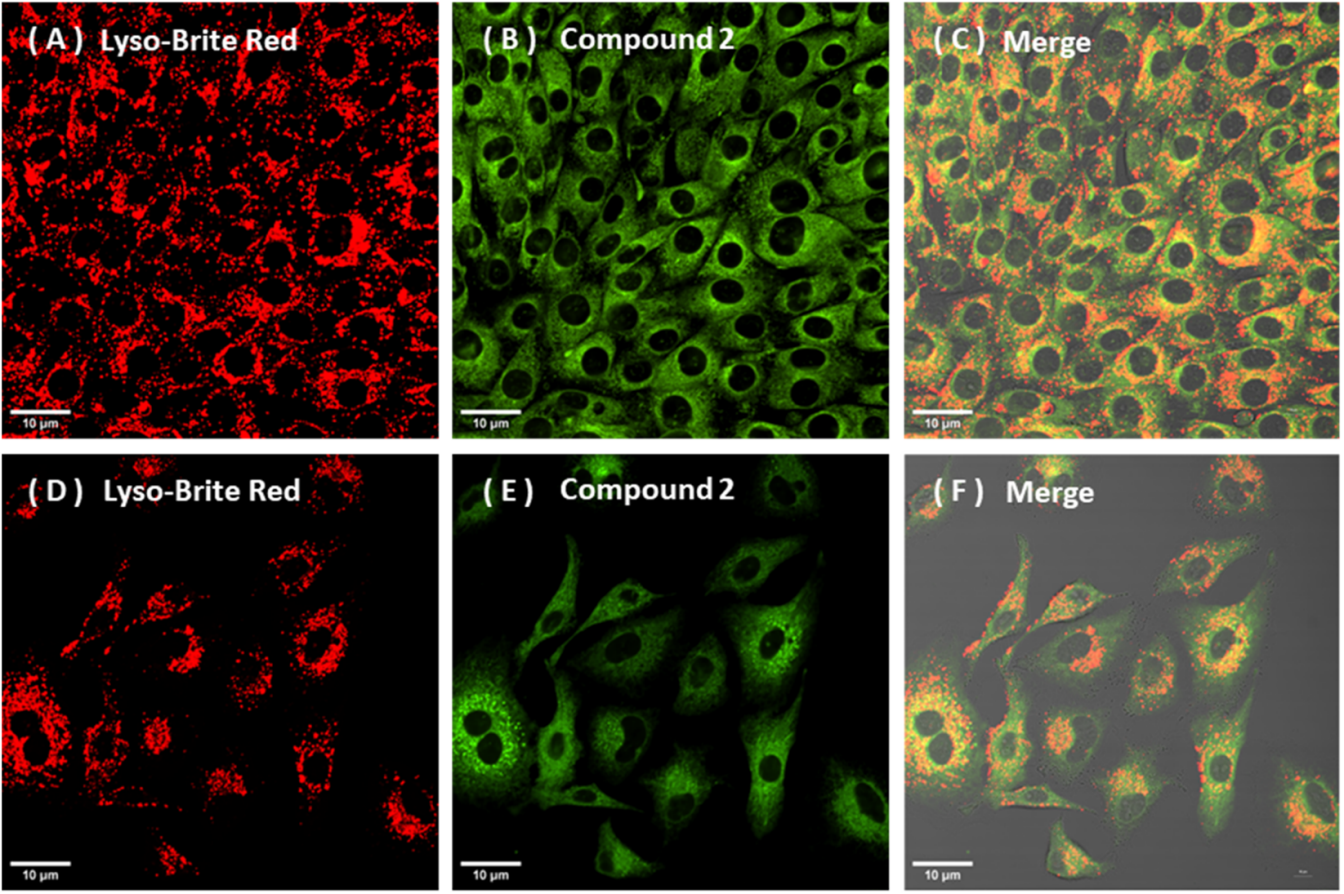
Confocal microscopy images showing the diffuse intracellular distribution of compound 2 without predominant lysosomal localisation in both NIH-3T3(upper) and A549 cells (lower): (A–C): NIH-3T3, 400 nM 60 min: LysoBrite Deep Red (A, *λ*_ex_ 561 nm, *λ*_em_ = 610–620 nm), compound 2 channel (B, *λ*_ex_ 445 nm, *λ*_em_ = 500–555 nm), merged image (C); (D–F): A549 cells, 400 nM 60 min: LysoBrite Deep Red (D), 2 channel (E), merged image (F). Pearson's *r* coefficient values were <0.15 for these sets). Scale bar: 10 µm.

**Fig. 8:**
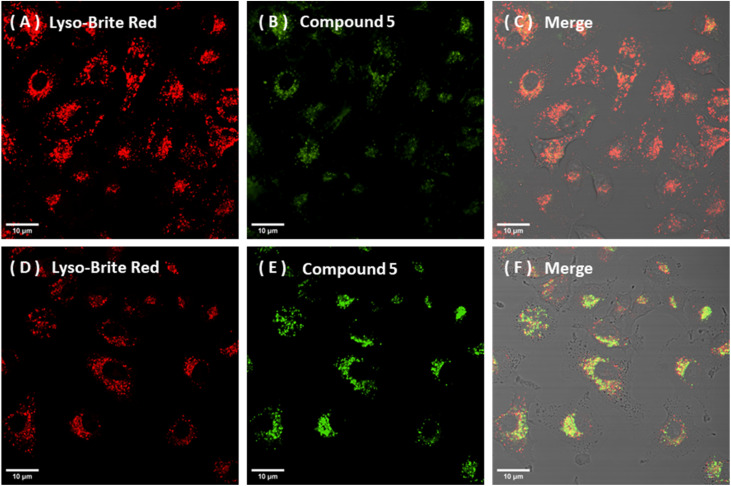
Confocal microscopy images showing the time and concentration-dependent lysosomal association of compound 5 in A549 cells: (A–C) A549, 25 nM 15 min: LysoBrite Deep Red (A, *λ*_ex_ 561 nm, *λ*_em_ = 610–620 nm), Compound 5 channel (B, *λ*_ex_ 445 nm, *λ*_em_ = 500–555 nm), and merged image (C). (D–F): A549, 50 nM 60 min: LysoBrite Deep Red (D), Compound 5 channel (E), merged image (F). G: 2D intensity histogram (5*vs.* LysoBrite Deep Red for (D–F), with Pearson's correlation coefficient *r* = 0.72(0.02). Scale bar: 10 µm.

In contrast, compounds 1 and 2 (Fig. 7 and S4), exhibited a diffuse, non-selective intracellular staining profile (*i.e.* a broad cytoplasmic distribution), without displaying any convincing evidence for organelle specificity under the tested conditions. Representative images together with ROI-based 2D intensity histograms and co-localisation metrics supported these conclusions (Fig. 6–8 and S1–S3).

### Preliminary cell toxicity screening

A standard MTT assay, probing perturbation to mitochondrial redox function, was used for each compound to assess cellular metabolic activity and thereby examine the effect of each compound on cell proliferation, viability and cytotoxicity.^27^ The vismodegib-conjugated probes (compounds 1–4) exhibited negligible toxicity across the range evaluated (30–1000 nM, 24 h), consistent with their observed biocompatibility in the live-cell imaging experiments. The cells maintained >80% viability with both A549 and NIH-3T3 cells, at working concentrations of 1 µM or less.

**Fig. 9:**
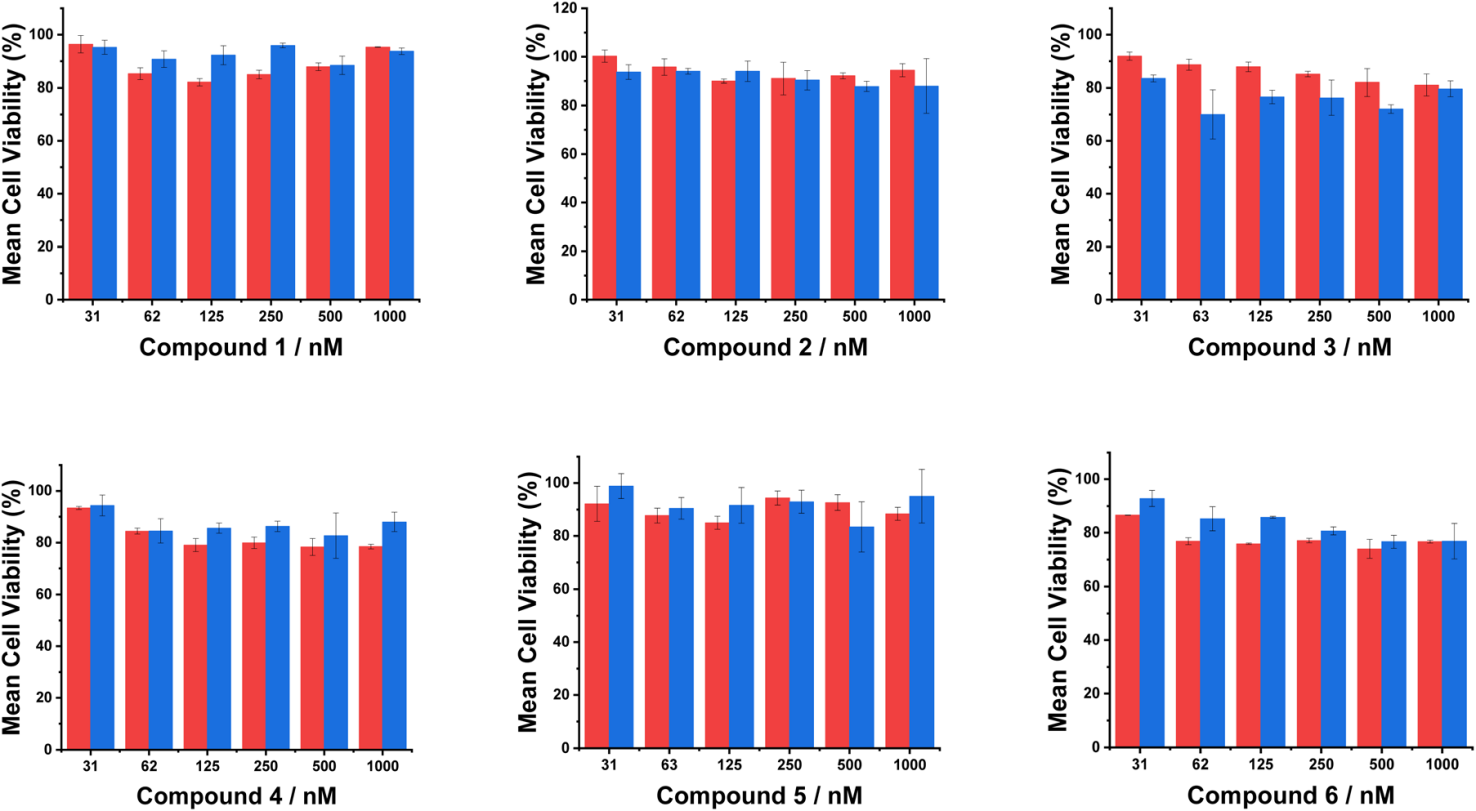
Cell viability data evaluated *via* the standard MTT assay, assessing mitochondrial redox function in A549 cells (red) and NIH-3T3 cells (blue) exposed to increasing concentrations of compounds 1–6 (31–1000 nM) for 24 h. Bar graphs summarise quantitative data with the mean values given showing the percentage standard deviation for three independent experiments (see SI for statistical methods).

For the Masupirdine conjugated systems, 5 and 6, hypothetically targeting the serotonin or 5-hydroxytryptamine GPCRs, no significant cytotoxicity was observed at concentrations up to 1 µM, with cell viability remaining >73%, compared to the untreated control (Fig. 9 and Table S1). The A549 cells were somewhat more sensitive to the presence of compounds 1, 4, and 6, while the NIH-3T3 cells were slightly more sensitive to the presence of 3 and 5. Compound 6 showed the strongest effect on A549 cells (74% viability at 500 nM), (Table S2).

## Conclusions

The design, synthesis and first phase characterisation of a family of six complementary GPCR-targeted probes has been achieved. For the first time in this sense, the conjugates are based on competitive antagonists that possess a nanomolar affinity for the primary binding GPCR receptor site. The key step in the next phase of this work is to assess their effect on the hedgehog and Serotonin signalling pathways and determine if they retain sufficient avidity for the particular cell surface receptor to allow them to be used as probes for the GPCR in over-expressed cell model experiments, or in competitive assays screening the effect of new drug candidates.

Each compound in the series of red and green fluorescent dyes, 1–6, showed no significant toxicity when administered to NIH-3T3 and A549 cells at sub-micromolar concentrations. The two neutral naphthalimide conjugates of vismodegib, 1 and 2, exhibited fast and non-specific cell uptake, as revealed by the confocal microscopy experiments, and showed no tendency to localise in the mitochondria or lysosomes. In contrast, the masupirdine–naphthalimide conjugate, 5, showed a rapid and pronounced staining of the lysosomes, staining selectively at 25 nM probe concentrations within 15 minutes. However, this probe is not likely to be of use on its own simply as a lysosomal stain, as there are many cheaper commercial stains that are used widely. Future work will assess the mechanism of its fast cell uptake process using appropriate promoters and inhibitors of the established cell uptake pathways.^28^

The anionic dye conjugates, 3, 4 and 6 showed no significant cell uptake in every experiment, which augurs well for their planned use to target over-expressed cell surface receptors in models with the smoothened and serotonin GPCRs. Further studies are underway to assess their suitability for this purpose and assess their scope as targeted FRET acceptors in time-resolved cellular assays, for example based on corresponding europium FRET donors.^23^

## Author contributions

The project was conceived by D. P.; the manuscript was written by D. P. with assistance from each co-author, especially C. A.; X. Z. carried the cell culture and confocal microscopy imaging, C. A. and T. L. C. undertook the compound syntheses and photophysical analyses; C. A. K. carried out the crystal structure determination and structural refinement; C. A. undertook measurements of protein binding constants; H. L. undertook the toxicity assays and assisted with microscopy experiments.

## Conflicts of interest

There are no conflicts to declare.

## Supplementary Material

RA-016-D6RA01214K-s001

RA-016-D6RA01214K-s002

## Data Availability

CCDC 2529009 and 2529010 contain the supplementary crystallographic data for this paper.^29*a*,*b*^ Electronic supplementary information (SI) is available which supports the findings of this study. Supplementary information: methods and materials, synthesis and characterisation, fluorescent studies, cell imaging and single crystal X-ray data. See DOI: https://doi.org/10.1039/d6ra01214k.

## References

[cit1] Sekulic A., Migden M. R., Oro A. E., Dirix L., Lewis K. D., Hainsworth J. D., Solomon J. A., Hauschild A. (2012). N. Engl. J. Med..

[cit2] Arensdorf A. M., Marada S., Ogden S. K. (2015). Trends Pharmacol. Sci..

[cit3] Corbit K. C., Aanstad P., Singla V., Norman A. R., Stainier D. Y. R., Reiter J. F. (2005). Nature.

[cit4] Byrne E. F. X., Sircar R., Miller P. S., Hedger G., Luchetti G., Nachtergaele S., Tully M. D., Mydock-McGrane L., Covey D. F., Rambo R. P., Sansom M. S. P., Newstead S., Rohatgi R., Siebold C. (2016). Nature.

[cit5] Nirogi R., Abraham R., Ravula J., Jetta S., Benade V., Shinde A. K., Rasheed M. A., Badange R. K., Goyal V. K., Palacharia V. R. C., Subramanian R., Jayarajan P. (2023). Alzheimer's Dement..

[cit6] Heretsch P., Tzagkaroulaki L., Giannis A. (2010). Angew. Chem., Int. Ed..

[cit7] Chen J. K., Taipale J., Cooper M. K., Beachy P. A. (2002). Genes Dev..

[cit8] Kikuchi K., Adair L. M., Xu Y., Ross H. J., Lu Z., Xie Y., Jacquemin D., Cox R. P., Bell T. D. M., Trevaskis N. L., New E. J., Kaur A. (2025). Chem.–Eur. J..

[cit9] Shindy H. A. (2017). Dyes Pigm..

[cit10] Ladokhin A. S., Haigler H. T. (2005). Biochemistry.

[cit11] Zhang P., Li J., Li B., Xu J., Lu J., Wu S. (2015). Chem. Commun..

[cit12] Lau J. Y., Shaffer C. C., Sanders H. S., Smith B. D. (2025). J. Phys. Chem. A.

[cit13] Xie X., Fan J., Liang M., Jiao X., Wang X., Tang B. (2017). Chem. Commun..

[cit14] Huang C., Jia T., Tang M., Yin Q., Zhang C., Yng Y., Jia N., Xu Y., Qian X. (2014). J. Am. Chem. Soc..

[cit15] Ernst S., Düser M. G., Zarrabi N., Dunn S. D., Börsch M. (2012). Biochim. Biophys. Acta, Bioenerg..

[cit16] Suzuki M., Shindo Y., Yamanaka R., Oka K. (2022). Sci. Rep..

[cit17] Danylchuik D. I., Khalin I., Suseela Y. V., Filser S., Plesnila N., Klymchenko A. S. (2025). Anal. Chem..

[cit18] Zhang Y., Bi J., Xia S., Mazi W., Mikesell L., Luck R. L., Liu H. (2018). Molecules.

[cit19] Delbianco M., Sadovnikova V., Bourrier E., Lamarque L., Mathis G., Zwier J. M., Parker D. (2014). Angew Chem. Int. Ed. Engl..

[cit20] Starck M., Pal R., Parker D. (2016). Chem.–Eur. J..

[cit21] Fradgley J. D., Starck M., Laget M., Bourrier E., Dupuis E., Trinquet L. L. E., Zwier J. M., Parker D. (2021). Chem. Commun..

[cit22] Panchuk-Voloshina N., Haugland R. P., Haugland R. P. (1999). J. Histochem. Cytochem..

[cit23] Cheung T.-L., Alexander C., Li H., Parker D. (2025). Chem. Commun..

[cit24] Kaduk J. A., Gates-Rector S., Blanton T. N. (2023). Powder Diffr..

[cit25] Dungrela D. (2024). “Bovine Serum Albumin (BSA): Structure, Properties, Applications, and Future Research Directions.”. BioScience ISSN.

[cit26] (b) Kuzmič P. , DYNAFIT – A software package for enzymology, Methods Enzymol, 2009, Vol. 467, Ch 10, pp. 247–280 10.1016/S0076-6879(09)67010-5 19897096

[cit27] Ghasemi M., Turnbull T., Sebastian S., Kempson I. (2021). Int. J. Mol. Sci..

[cit28] New E. J., Congreve A., Parker D. (2010). Chem. Sci..

[cit29] (a) CCDC 2529009: Experimental Crystal Structure Determination, 2026, 10.5517/ccdc.csd.cc2qwmy6

