# Supplementary material for: Synthesis and preliminary assessment of fluorescent probes based on the competitive GPCR antagonists vismodegib and masupirdine
Source: RSC Adv. 2026 Apr 1;16(20):17745–52. doi: 10.1039/d6ra01214k (PMC13041632; doi:10.1039/d6ra01214k)
Supplement: RA-016-D6RA01214K-s001 [file RA-016-D6RA01214K-s001.pdf]

Supplementary Information (SI) for RSC Advances.
This journal is © The Royal Society of Chemistry 2026

Supporting Information

Synthesis and Preliminary Assessment of Fluorescent Probes Based on the Competitive GPCR Antagonists Vismodegib and Masupirdine

Carlson Alexander,[a] Tsz-Lam Cheung,[a] Chowan Ashok Kumar,[a] Catherine Hong Huan Hor,[a] Huishan Li,[a] Xiuzhi Zou[a] and David Parker[a]*

[a]Department of Chemistry, Hong Kong Baptist University, Kowloon Tong, Hong Kong SAR, China.

*Correspondence to davidparker@hkbu.edu.hk

Contents to supporting information Page No.

1. Abbreviations ----------S3

2. Materials and solvents ----------S3
   2.1 Reagents and solvents ----------S3
   2.2 Vacuum processing, TLC, flash and column chromatography, pH ----------S4
   2.3 Cellular studies ----------S5

3. Physical methods ----------S5
   3.1 NMR spectroscopy ----------S5
   3.2 Mass spectroscopy and HPLC ----------S5
   3.3 Photophysical methods ----------S6
   3.4 X-ray crystallography ----------S6

4. Statistical analysis ----------S7
   4.1 Binding isotherms ----------S7
   Table S1. Statistical analysis on binding isotherms produced using DYNAFIT® S8

5. Cell culture and microscopy studies ----------S8
   5.1 Cell line and culture ----------S8
   5.2 MTT cell viability assay ----------S8
   Table S2. Summary of the MTT cell viability assay results ----------S9
   5.3 Cell imaging ----------S9
      Fig. S1 Confocal microscopy image for compound **5** ----------S10
      Fig. S2 Confocal microscopy image for compound **2** ----------S11
      Fig. S3 Confocal microscopy image for compound **1** ----------S11

6. Synthesis of compounds ----------S12
   Scheme S1. Synthetic scheme for compounds **5**, **9**, **12**, **15** and **Masupirdine**-------S12
   Scheme S2. Synthesis of compound **1** ----------S13
   Scheme S3. Synthesis of compound **2** ----------S13
   Scheme S4. Synthesis of compound **4** ----------S14
   Scheme S5. Synthesis of compound **6** ----------S14
   Synthesis of compound **7** ----------S15
   Synthesis of compound **8** ----------S15
   Synthesis of compound **9** ----------S16
   Synthesis of compound **5** ----------S17
   Synthesis of compound **1** ----------S18

Synthesis of compound **16** --S19
Synthesis of compound **17** --S20
Synthesis of compound **18** --S20
Synthesis of compound **2** --S21
Synthesis of compound **19** --S22
Synthesis of compound **4** --S23
Synthesis of compound **20** --S23
Synthesis of compound **6** --S24

7. Selected High-Resolution Mass and NMR spectra ------------------------------------S25
Fig. S4 MALDI-TOF mass spectrum of compound **8** ---------------------------S25
Fig. S5 MALDI-TOF mass spectrum of compound **9** ---------------------------S25
Fig. S6 MALDI-TOF mass spectrum of compound **5** ---------------------------S26
Fig. S7 MALDI-TOF mass spectrum of compound **1** ---------------------------S26
Fig. S8 MALDI-TOF mass spectrum of compound **16** ---------------------------S27
Fig. S9 MALDI-TOF mass spectrum of compound **17** ---------------------------S27
Fig. S10 MALDI-TOF mass spectrum of compound **18** ---------------------------S28
Fig. S11 MALDI-TOF mass spectrum of compound **2** ---------------------------S28
Fig. S12 400 MHz ^{1}H NMR spectrum of compound **8** in $CDCl_3$ at 294 K -----------S29
Fig. S13 101 MHz ^{13}C NMR spectrum of compound **8** in $CDCl_3$ at 294 K ----------S29
Fig. S14 400 MHz ^{1}H NMR spectrum of compound **9** in CD_3OD at 294 K ---------S30
Fig. S15 101 MHz ^{13}C NMR spectrum of compound **9** in CD_3OD at 294 K ---------S30
Fig. S16 600 MHz ^{1}H NMR spectrum of compound **5** in $CDCl_3$ at 294 K ---------S31
Fig. S17 151 MHz ^{13}C NMR spectrum of compound **5** in $CDCl_3$ at 294 K ---------S31
Fig. S18 600 MHz ^{1}H NMR spectrum of compound **1** in $CDCl_3$ at 296 K ---------S32
Fig. S19 151 MHz ^{13}C NMR spectrum of compound **1** in $CDCl_3$ at 296 K ---------S32
Fig. S20 400 MHz ^{1}H NMR spectrum of compound **16** in $CDCl_3$ at 294 K ---------S33
Fig. S21 101 MHz ^{13}C NMR spectrum of compound **16** in $CDCl_3$ at 294 K ---------S33
Fig. S22 400 MHz ^{1}H NMR spectrum of compound **17** in $CDCl_3$ at 294 K ---------S34
Fig. S23 101 MHz ^{13}C NMR spectrum of compound **17** in $CDCl_3$ at 294 K ---------S34
Fig. S24 600 MHz ^{1}H NMR spectrum of compound **18** in DMSO-d_6 at 296 K ------S35
Fig. S25 151 MHz ^{13}C NMR spectrum of compound **18** in DMSO-d_6 at 296 K -----S35
Fig. S26 400 MHz ^{1}H NMR spectrum of compound **2** in $CDCl_3$ at 294 K -----------S36
Fig. S27 151 MHz ^{13}C NMR spectrum of compound **2** in $CDCl_3$ at 296 K ----------S36

8. Photophysical studies --S37
Fig. S28 Absorption spectrum of **Vismodegib** in HEPES buffer ------------------S37
Fig. S29 Absorption titration spectra of **Vismodegib** with BSA in HEPES buffer---S37

Fig. S30 Fluorescence titration spectra of **Vismodegib** with BSA in HEPES buffer-S38
Fig. S31 Absorption spectra of **1** at varying pH ----------------------------------S38
Fig. S32 Fluorescence spectra of **1** at varying pH ----------------------------------S39
Fig. S33 Absorption titration spectra of **1** with BSA in HEPES buffer ---------S39
Fig. S34 Fluorescence titration spectra of **1** with BSA in HEPES buffer ---------S40
Fig. S35 Absorption spectra of **2** at varying pH ----------------------------------S40
Fig. S36 Fluorescence spectra of **2** at varying pH ----------------------------------S41
Fig. S37 Absorption titration spectra of **2** with BSA in HEPES buffer ---------S41
Fig. S38 Absorption spectra of **5** at varying pH ----------------------------------S42
Fig. S39 Fluorescence spectra of **5** at varying pH ----------------------------------S42
Fig. S40 Absorption titration spectra of **5** with BSA in HEPES buffer ---------S43
Fig. S41 Fluorescence titration spectra of **5** with BSA in HEPES buffer ---------S43
Fig. S42 Absorption titration spectra of **6** with BSA in HEPES buffer ---------S44
Fig. S43 Fluorescence titration spectra of **6** with BSA in HEPES buffer ---------S44
Fig. S44 Molar extinction coefficient of **Vismodegib** in DMSO ------------------S45
Fig. S45 Molar extinction coefficient of **Masupirdine** in DMSO ------------------S45
Fig. S46 Molar extinction coefficient of **1** in DMSO --------------------------S46
Fig. S47 Molar extinction coefficient of **2** in DMSO --------------------------S46

9. X-ray crystal structure data ---S47
Table S3. Crystal data and structure refinement for **Masupirdine** and compound **12** ------S47

10. References---S48

1. Abbreviations

RT	room temperature
BSA	bovine serum albumin
HEPES	4-(2-hydroxyethyl)piperazine-1-ethanesulfonic acid
DCTB	2-[(2E)-3-(4-*tert*-butylphenyl)-2-methylprop-2-enylidene]malononitrile
HATU	1-[bis(dimethylamino)methylene]-1*H*-1,2,3-triazolo[4,5-b]pyridinium-3-oxide hexafluorophosphate
EDC	1-(3-Dimethylaminopropyl)-3-ethylcarbodiimide hydrochloride
TFA	trifluoroacetic acid
DMSO	dimethyl sulfoxide
DMF	*N*,*N*-dimethyl formamide
DMAP	4-(Dimethylamino)pyridine
MTT	3-(4,5-dimethylthiazol-2-yl)-2,5-diphenyltetrazolium
FBS	foetal bovine serum
PBS	Phosphate buffered saline
A549	human lung adenocarcinoma
NIH-3T3	mouse skin fibroblasts

2. Materials and solvents

2.1 Reagents and solvents

Methanol (99.9%, HPLC grade), ethanol (99.8%, absolute), ethyl acetate (99.8%, GR ACS ISO), sodium acetate (99.0~101.0%, GR ACS), diethyl ether (99.5% GR), isopropanol (GR), *n*-hexane (95% GR) (International Laboratory USA); trifluoroacetic acid (99%), deuterium oxide (99.9% atom % D) (Sigma Aldrich); hydrochloric acid (37%) (VWR Chemicals); dimethyl sulfoxide (GR) (Duksan); chloroform (AR) (ACI labscan); acetone (lab grade, UN1000, Class 3, PGII), dichloromethane (AR), tetrahydrofuran, (Standard chemical, STC); *N*-Boc ethylenediamine (98%), 2-methoxyethanol (Ar charged, AR), (Aladdin chemicals); sodium sulfate (AR), potassium hydroxide (AR), sodium chloride (AR), *n*-propylamine (AR), 4-bromo-1,8-naphthalic anhydride (98%), oxalyl chloride (98%), *tert*-butyl (5-aminopentyl)carbamate (97%), DMAP (99%) (Dieckmann); 6-aminohexanoic acid (>98%) (TCI); *N,N*-Diisopropylethylamine (≥99.7%), HATU (99%), BSA (98%), HEPES sodium salt (99%), acetic acid (ACS, ≥99.7%) (Macklin); DMF (99.6%, extra dry with molecular sieves), EDC (99%) (Energy Chemicals); Chloroform (D, 99.8% + 0.03% *v/v* TMS + silver foil), DMSO-d_6 (D, 99.9%) (Cambridge isotope laboratories, Inc.); methanol-d_4 (99.8 atom % D) (thermo scientific); Vismodegib, 3-(6-(2-chloro-5-(2-chloro-4-(methylsulfonyl)benzamido)phenyl)pyridin-3-yl)propanoic acid, and *N*-(3-(5-(3-aminopropyl)pyridin-2-yl)-4-chlorophenyl)-2-chloro-4-(methylsulfonyl)benzamide were synthesised by Pharmaron, Inc. in Beijing, China. The dye **Dy647-COOH** was a gift from Cisbio Bioassays (Revitty; Codolet, France) and the **AF532-NHS ester** was purchased from BT-Probes®. Deionised water was used throughout the study, obtained from Millipore ultra purification system with an ionic conductance of 18 MΩ.cm.

2.2 Vacuum processing, TLC, flash and column chromatography, pH

A Heidolph Laborota 4000-efficient rotary evaporator was used to evaporate solvents under reduced pressure and compounds were dried using a Schlenk line with a Titan RV3 Vacuum Pump. Filtrations were performed under gravity using a Whatman filter paper (grade 1 circles) on a glass funnel or using celite (Aladdin Chemicals) under vacuum. Column chromatography was performed using silica gel (100-200M mesh, Bidepharm) and flash column chromatography using Biotage® Isolera system 3.3.1. Analytical thin layer chromatography (TLC) was performed on aluminium sheet supported silica gel plates coated with silica gel 60 F-254 (0.2 mm, Merck) using different solvent systems as mobile phase.

The compounds were visualised in TLC by potassium permanganate stain prepared as per literature protocol[1] and under UV light. Preparative TLC was performed on a glass backed silica plate from Analtech Preparative Uniplates (20 × 20 cm), 500mm thick/15 μm particle. For absorption and fluorescence emission of compounds recorded at different pH, pH 4.0 – 5.5 was maintained using 0.01M acetate buffer, 6.5 – 8.0 was maintained using 0.01M HEPES buffer. The pH was measured using Leici PHS-3E pH meter and was calibrated by three standard buffer solutions (pH 4.00, 6.86 and 9.18 at 298K) with standard error of pH 0.01 before every measurement.

2.3 Cellular studies

Mouse embryo NIH-3T3 (ATCC®CRL-1658™) fibroblasts were purchased from ATCC (Manassas, VA). Human lung cancer A549 cells were obtained from the Cell Culture Bank of the Chinese Academy of Sciences' Type Culture Collection Committee (Shanghai). Mito-Tracker Deep red 633 was procured from Beyotime and LysoBrite™ Deep red from AAT Bioquest®. MTT was purchased from Aladdin Chemicals; PBS from Gibco (KH_2PO_4 1.05 mM + $Na_2HPO_4^-.7H_2O$ 2.96 mM + NaCl 155 mM, 1X, pH 7.4, sterile filtered, reference 10010-023).

3. Physical methods

3.1 NMR Spectroscopy

NMR spectra were recorded using a Bruker Ascend™ 400 MHz NMR spectrometer or Bruker Avance Neo 600 MHz NMR spectrometer, at the stated ambient temperatures. All NMR spectra were recorded in deuterated solvents. Chemical shifts were assigned by comparison with the residual proton and carbon resonances of the solvents.[2] The recorded free induction decays were processed using backward linear prediction, optimal exponential weighting, zero-filling, Fourier transform, phasing, and baseline corrected when necessary.

3.2 Mass spectroscopy and HPLC

MALDI-TOF mass spectra were recorded on a Bruker Daltonics autoflex® maX LRF MALDI-MS system. All MALDI-TOF MS samples were run in a DCTB matrix. HPLC

analysis and purification was performed at 295 K with two different set-ups. All chromatograms were reported at 254nm.

Agilent system: Agilent 1100 module HPLC system (Agilent Technologies, Stockport, UK), G1313A Autosampler (Micro-WPS), G1312A Binary Pump, G1315A Diode-Array Detector (DAD) and Agilent 5 HC-C18 (2) column (5 µm, 4.6 × 250 mm).

Shimadzu system. Semi-preparative High Performance Liquid Chromatograph LC-20AR, LC-20AR Solvent Delivery Pump, DGU-40 Degassing unit, LH-40 Liquid Handler, SPD-M40 Photodiode Array Detector, FRC-40 Fraction Collector, CBM-40 System Controller and XBridge® Prep C18 OBD™ column (5 µm, 19 × 100 mm).

Various chromatographic systems were employed for analytical and preparative HPLC:

Method A: (Agilent system) flow rate 1.0 mL/min with H_2O (0.1% TFA) – 20% MeCN (0.1% TFA) as eluents (linear gradient to 80% MeCN (0.1% TFA) [20 min].

Method C: (Agilent system) flow rate 0.5 mL/min with H_2O (0.1% TFA) – 10% MeCN (0.1% TFA) as eluents (linear gradient to 50% MeCN (0.1% TFA) [40 min].

Method D: (Agilent system) flow rate 0.5 mL/min with H_2O (0.1% TFA) – 5% MeCN (0.1% TFA) as eluents (linear gradient to 30% MeCN (0.1% TFA) [40 min].

Method H: (Shimadzu system) flow rate 5.0 mL/min with H_2O (0.1% TFA) – 20% MeCN (0.1% TFA) as eluents (linear gradient to 45% MeCN (0.1% TFA) [40 min].

Method L: (Shimadzu system) flow rate 5.0 mL/min with H_2O (0.1% TFA) – 30% MeCN (0.1% TFA) as eluents (linear gradient to 70% MeCN (0.1% TFA) [40 min].

3.3. Photophysical measurements

Electronic absorption measurements were recorded on an Agilent Cary 60 spectrophotometer operated under Cary WinUV software. Points were recorded at 1 nm intervals. Steady-state excitation and emission spectra were recorded on a Horiba Jobin Yvon FluoroMax® 4 Fluorimeter equipped with a 450 W xenon lamp operated under FluorEssence™ (v3.8) software for Windows. The signal was detected by a Hamamatsu R928 photomultiplier tube. Points were recorded at 1 nm intervals with an 0.5 s integration time.

Excitation and emission spectra were measured with entrance slit ranging from 5 nm to 1 nm while the exit slit was set to 1 nm or 0.5 nm. The absorption and fluorescence measurements were carried out for solutions of samples in 3500 µL quartz macro fluorescence cuvettes (1.05m, four-way slit 3mm cuvette, LG07-104-4C, Guanghou Lige technology Co., Ltd.). All measurements were performed at ambient temperature (22 °C) and the averaged value from 3 independent measurements was used.

3.4 X-ray crystallography

Crystallography data was collected using either a Rigaku Oxford Diffraction Synergy-S diffractometer with a dual source equipped with a Hybrid pixel array detector or using Kα radiation (λ = 0.71073 Å) on a Bruker D8V Venture (Photon II 14 detector, IµS 3.0 microfocus sealed tube sources (Cu and Mo) diffractometer) equipped with a Cobra low temperature device. The crystal was kept at 302 K or 100 K during data collection. Using Olex2,[4] the structure was solved with the ShelXS[5] structure solution program using Direct Methods and refined with the ShelXL[6] refinement package using Least Squares minimisation. All non-hydrogen atoms were refined anisotropically; hydrogen atoms were placed in the calculated positions and refined in riding mode. X-ray crystal structure images were produced using Mercury CSD 3.6 software using data from the Cambridge Structural Database. **Masupirdine** was crystallised by vapour diffusion of hexane into concentrated solution of the compound in dichloromethane. Compound **12** was crystallised by vapour diffusion of hexane into a concentrated solution of the compound in chloroform.

4. Statistical analysis

4.1 Binding isotherms

The fluorescent compound conjugated to vismodegib or masupirdine (host, 1.5 mL) was taken in the cuvette and BSA was added in aliquots using a Gilson® micropipette. Each addition was mixed by agitation using a 1000 µL pipette inside the cuvette, left aside for a minute and the spectral measurements (absorption/excitation/emission) were taken. The change in steady-state fluorescence emission maxima was plotted as a function of the concentration of added BSA and the concentration of BSA was determined alongside a known concentration curve. The resulting titration curve, known as the binding isotherm, was fitted to the NL2SOL algorithm corresponding to the postulated chemical equilibrium (eq. 1) to obtain the association constants for a single binding event (log *K*), via an iterative least

square fitting process using DYNAFIT® version 4 (BioKin, Ltd.), with a confidence interval at the 95% probability level where uncertainty (±) is expressed as coefficient of variation in percentage.[3]

$$[host] + [BSA] \overset{K}{\Leftrightarrow} [host - BSA] \qquad K = \frac{[host - BSA]}{[host][BSA]} \qquad (1)$$

Fitting of the binding isotherm to multiple binding events did not produce a meaningful binding isotherm. Statistical analysis data for the final binding isotherm are tabulated in Table S1. Values for the coefficients of variation in percentage, stock concentration of the host and BSA are mentioned under each binding isotherm.

Table S1. Statistical analysis on binding isotherms produced using DYNAFIT®

**Regression summary**	**Vismodegib vs BSA**	**1 vs BSA**	**2 vs BSA**	**5 vs BSA**
Unweighted sum of squares	3.2784 × 108	160804	172537	29655.8
Weighted sum of squares	3.2784 × 108	160804	172537	29655.8
Mean square	1.92847 × 107	5187.23	9080.91	1482.79
Root Mean Square deviation	4391.44	72.0225	95.2938	38.507
Relative Root Mean Square %	3.00116	1.8777	2.43004	0.797564
R-squared	0.990133	0.995512	0.990336	0.992042
Adjusted R-squared	0.990133	0.995512	0.990336	0.992042
Standard error (μM)	5300	1400	150000	7200
Coefficient of variation (range, μM)	1.79 – 2.01 (×105)	5.41 – 5.98 (×104)	1.15 – 1.78 (×106)	1.63 – 1.93 (×105)
Mean expected number of runs of signs	9.5	15.2	10.3	11
Expected standard deviation	2	2.5	2.1	2.2
Pearson's p-value	0.025	0.038	0.27	0.25
Binding isotherm location	Figure S30	Figure S34	Figure 5	Figure S42

5. Cell Culture and Microscopy studies

5.1 Cell line and culture

Human lung adenocarcinoma A549 and mouse skin fibroblasts NIH-3T3 cells were used in the study. They were cultivated in Dulbecco's Modified Eagle Medium (DMEM) supplemented with 10% foetal bovine serum (FBS) and 1% penicillin/streptomycin

antibiotics (Thermo-Fisher Scientific Inc, USA). All cells are cultivated in a humidified incubator with 5% CO_2 at 37 °C.

5.2 MTT cell viability assay

Two cell lines (A549 and NIH-3T3) (1×10^4 cells/mL) were seeded on 96-multiwell plates overnight in a humidified incubator at 37 °C under 5% CO_2 atmosphere. Different concentrations of each compound were added to the cells. After a 24 h incubation, 3-(4,5-dimethylthiazol-2-yl)-2,5-diphenyltetrazolium (MTT) (0.5 mg/mL) was added to each well, followed by incubation for 4 h at the same conditions. Additionally, 6 wells per plate with 100 µL of PBS as negative controls, adding 10 µL of PBS (no MTT) to three of them, and 10 µL of 5 mg/mL MTT-PBS solution to the remaining three. The formazan formed was dissolved in DMSO, and the absorbance of each well was measured using a microplate reader (PHERAstar FSX) at 570 nm (reference wavelength set to 690 nm) at ambient temperature. Triplicate measurements were performed and averaged to obtain the stated data. OD values were normalized to the untreated control group (set as 100% viability) and blank-corrected using medium-only wells. Cell viability (%) was calculated using the formula (2).[7,8]

$$Cell\ viability\ (\%) = \frac{OD_{Sample} - OD_{blank}}{OD_{control} - OD_{blank}} \times 100\% \qquad (2)$$

Table S2. Summary of the MTT cell viability assay results.

Compound	Approx. IC_{50} for A549	Approx. IC_{50} for NIH-3T3	Lowest viability A549	Lowest viability NIH-3T3
**1**	~125 nM	>1000 nM	82% (125 nM)	88% (500 nM)
**2**	>1000 nM	>1000 nM	90% (125 nM)	88% (500 nM)
**3**	~1000 nM	~63 nM	81% (1000 nM)	70% (63 nM)
**4**	~125 nM	>1000 nM	78% (500 nM)	83% (500 nM)
**5**	>1000 nM	~500 nM	85% (125 nM)	83% (500 nM)
**6**	~500 nM	~500 nM	74% (500 nM)	77% (500 nM)

5.3 Cell Imaging

For cell imaging, A549 and NIH-3T3 cells were seeded onto glass-bottom confocal dishes (5×10^4 cells mL^{-1}, 1.5 mL per dish) and allowed to adhere for 24 to 48 h at 37 °C, under 5% CO_2. Stock solutions of compounds **2** and **5** were prepared in DMSO (5: 1 mM; 2:

12 mM) and diluted into a pre-warmed culture medium immediately before use (final DMSO ≤ 0.01% *v*/*v*). Cells were treated with compound **5** (25 nM, 15 min; 50 nM, 60 min) or compound **2** (400 nM, 60 min) and were protected from light.

For co-localisation studies, MitoTracker Deep Red (50 nM) was added 10–15 min prior to imaging and LysoBrite™ Deep Red was added 30 min prior to imaging. Cells were washed twice with pre-warmed PBS and imaged in fresh medium using a Nikon AXR confocal microscope. Imaging parameters with compounds **2** and **5** were: λ_{ex} = 445 nm, λ_{em} = 500–555 nm; MitoTracker Deep Red, λ_{ex} = 640 nm, λ_{em} = 660–740 nm; LysoBrite™ Deep Red, λ_{ex} = 561 nm, λ_{em} = 610–620 nm (561 nm was the closest available excitation wavelength to the manufacturer-reported absorption maximum).

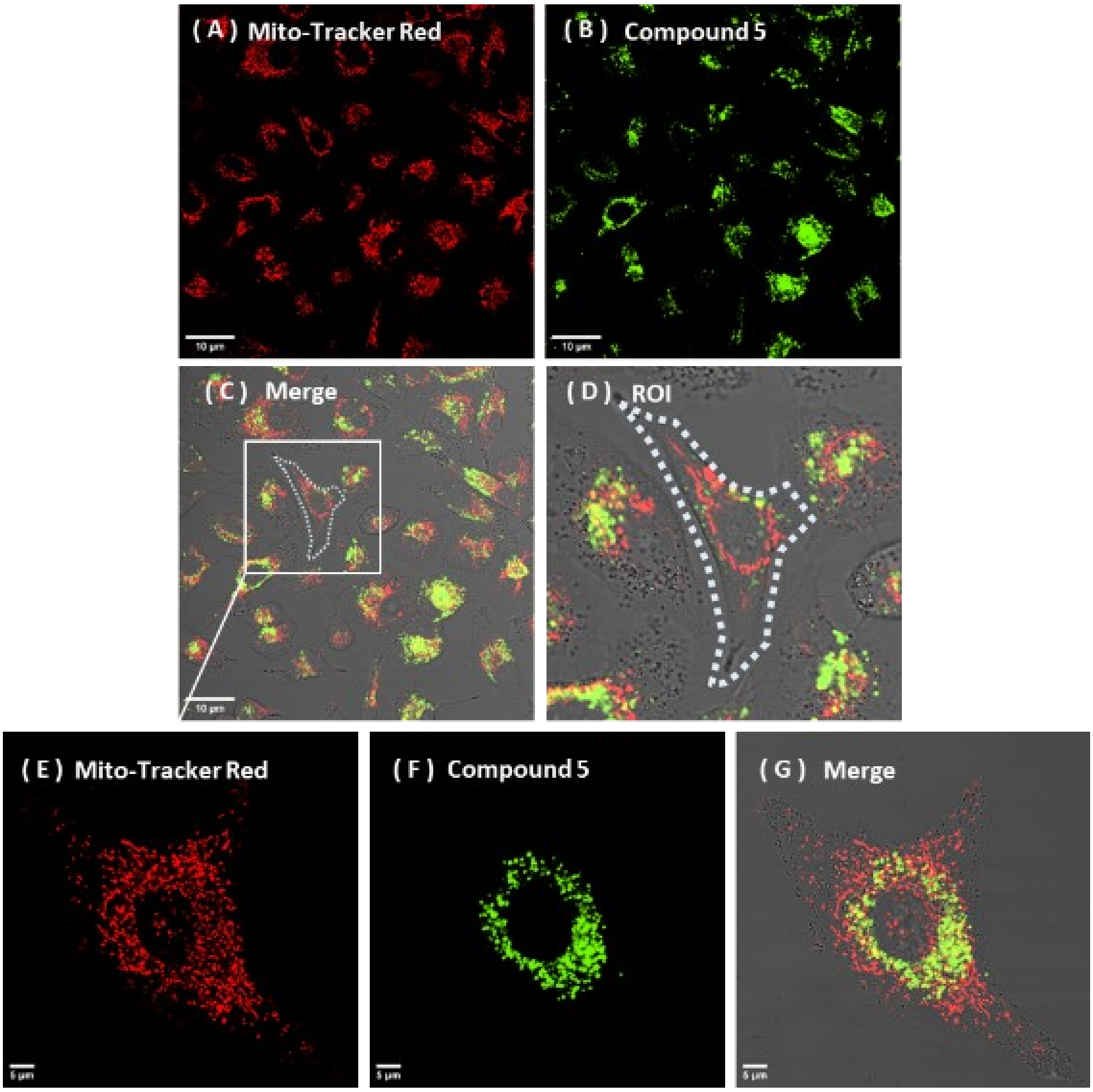

Figure S1. Confocal microscopy images revealing limited mitochondrial association of compound **5** in A549 cells under two incubation conditions: (A–D) 25 nM,15 min: Mito-

Deep Red channel (λ_{ex} = 640 nm, λ_{em} = 660–740 nm) (A), compound **5** channel (λ_{ex} = 445 nm, λ_{em} = 500–555 nm) (B), merged image (C) and (D); (E–G): 50 nM, 60 min: Mito Tracker Deep Red channel (E), compound **5** channel (F), and merged image (G); Pearson's coefficient, *r* for the merged image was less than 0.05. Scale bar for images (A-C) = 10 µm, (E-G) = 5 µm.

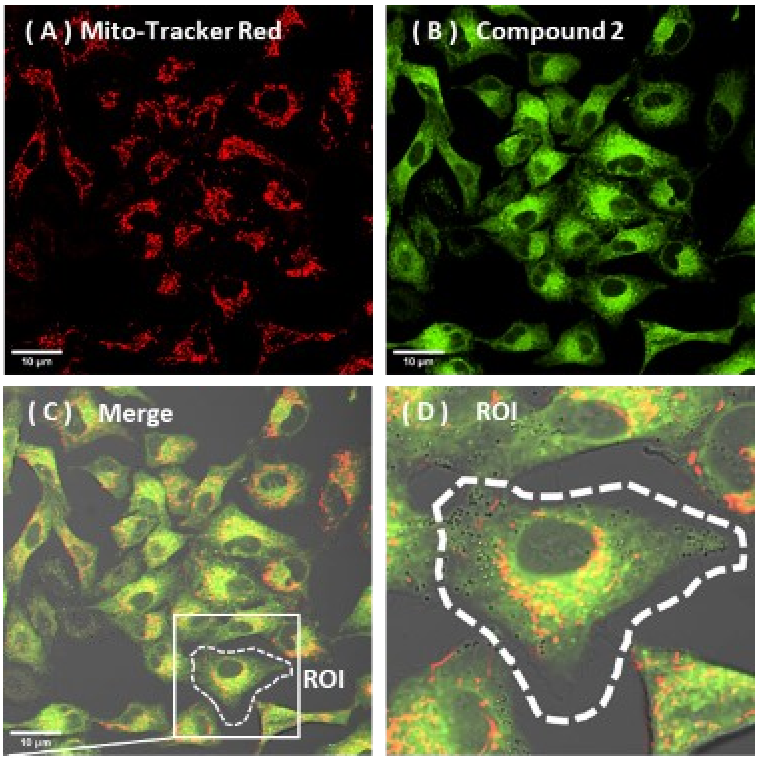

Figure S2. Confocal microscopy images showing the intracellular distribution of compound **2** relative to Mito Tracker Deep Red in A549 cells: (A–D) 400 nM, 60 min: Mito Tracker Deep Red (λ_{ex} = 640 nm, λ_{em} = 660–740 nm) (A), compound **2** channel (λ_{ex} = 445 nm, λ_{em} = 500-555 nm) (B), merged image with ROI indicated (C), and enlarged ROI (D), (Pearson's correlation *r* = 0.38). Scale bar for images (A-C) = 10 µm.

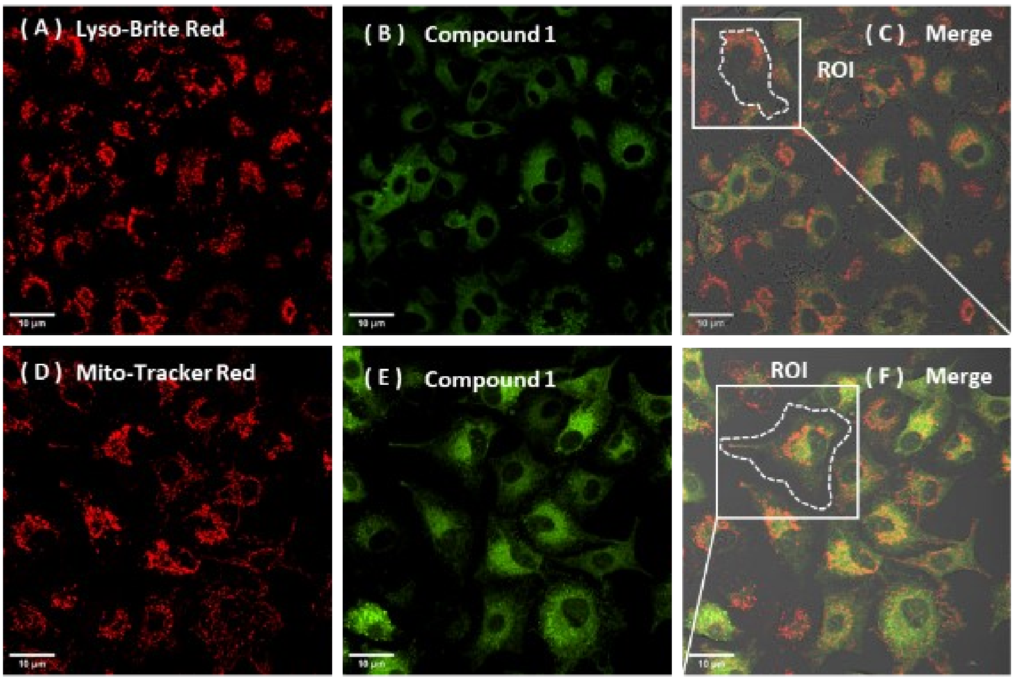

Figure S3. Confocal microscopy images showing the diffuse intracellular distribution of **1** in A549 cells; (A–C) A549, 1000 nM, 60 min (lysosomal staining): LysoBrite™ Deep Red channel (λ_{ex} = 561 nm, λ_{em} = 610–620 nm) (A), Compound **1** channel (λ_{ex} = 445 nm, λ_{em} = 500-555 nm) (B), merged image with ROI indicated (C). (D–F) A549, 1000 nM, 60 min (mitochondrial staining): MitoTracker Deep Red channel (λ_{ex} = 640 nm, λ_{em} = 660–740 nm) (D), Compound **1** channel (E), merged image with ROI indicated. Pearson's *r* values were < 0.03. Scale bar =10 µm.

6. Synthesis of compounds

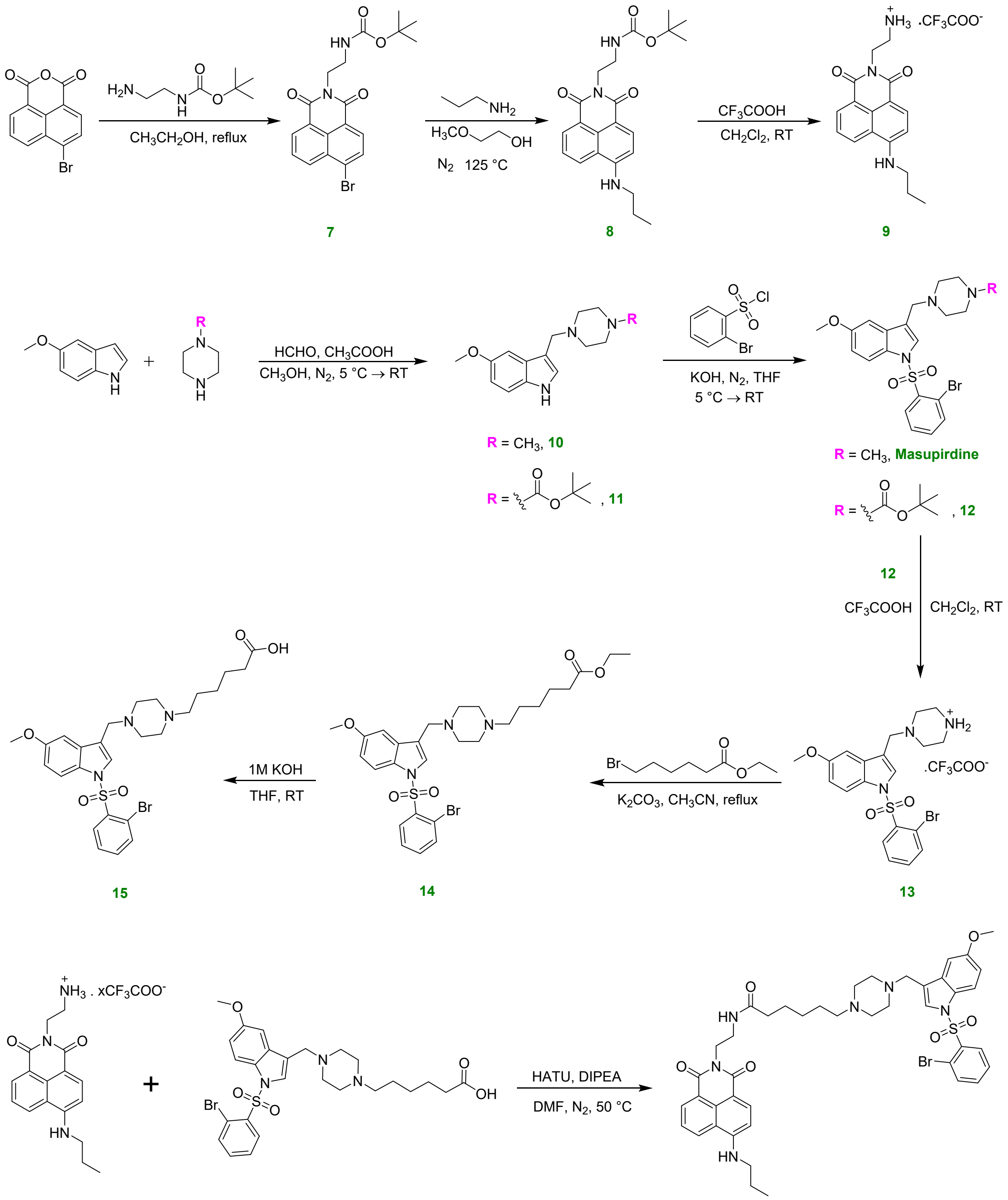

Scheme S1. Synthetic scheme for compounds **5**, **9**, **12**, **15** and **Masupirdine**.

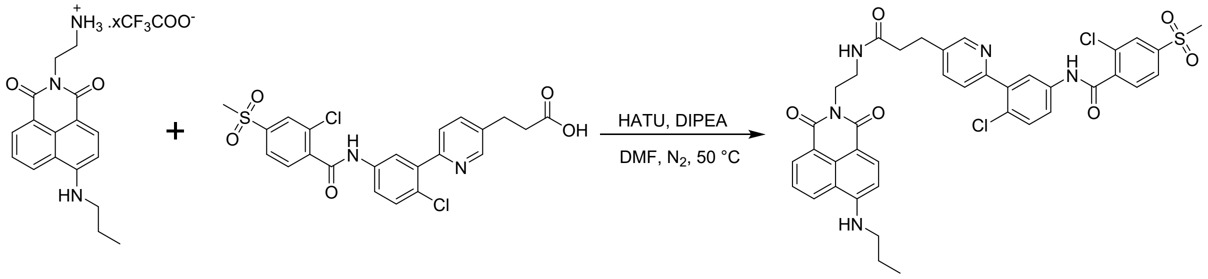

9

1

Scheme S2. Synthesis of compound **1**.

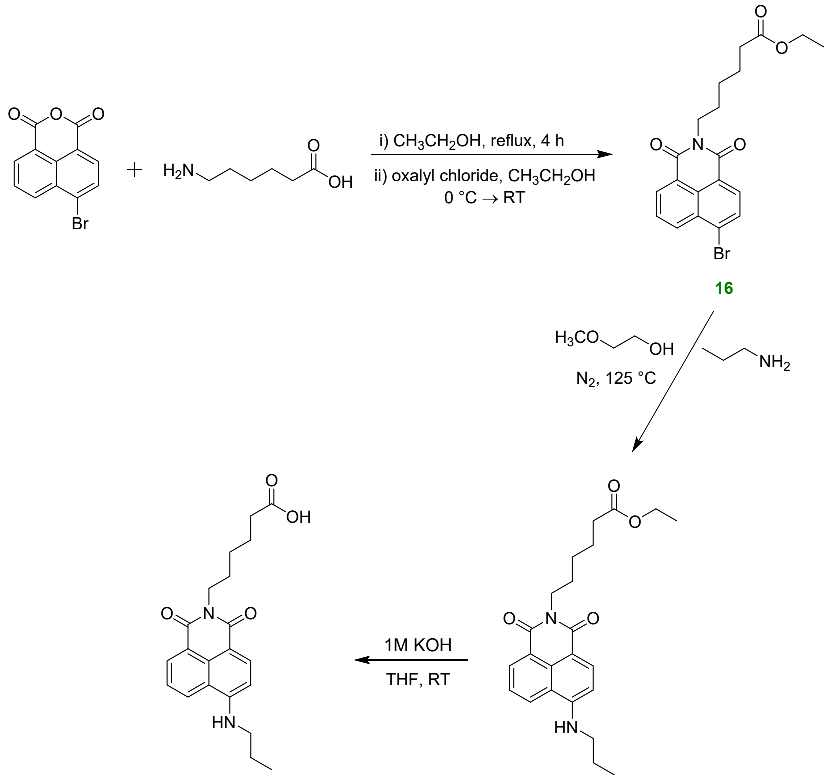

16

18

17

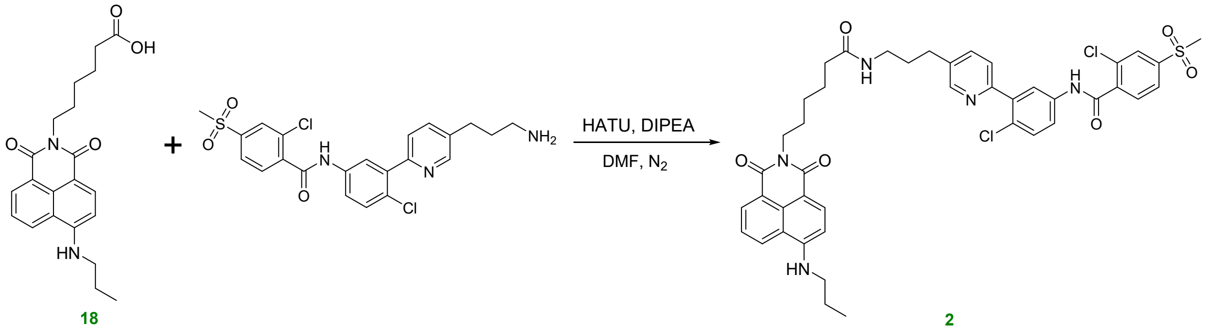

18

2

Scheme S3. Synthesis of compound **2**.

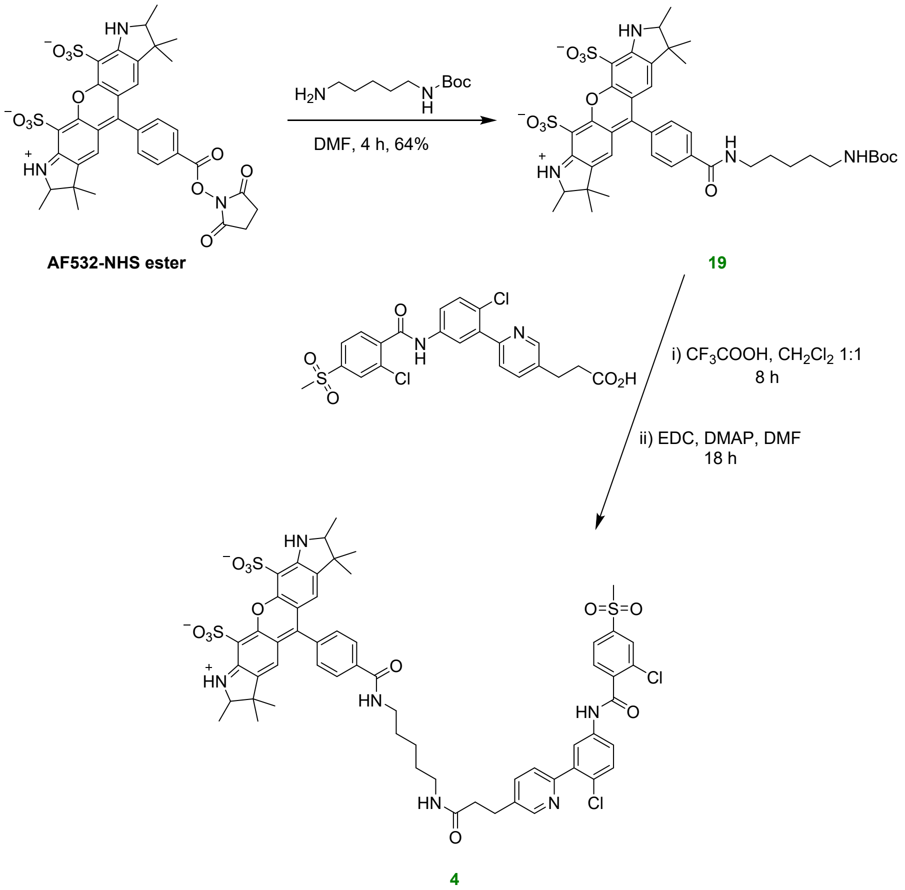

Scheme S4. Synthesis of compound **4**.

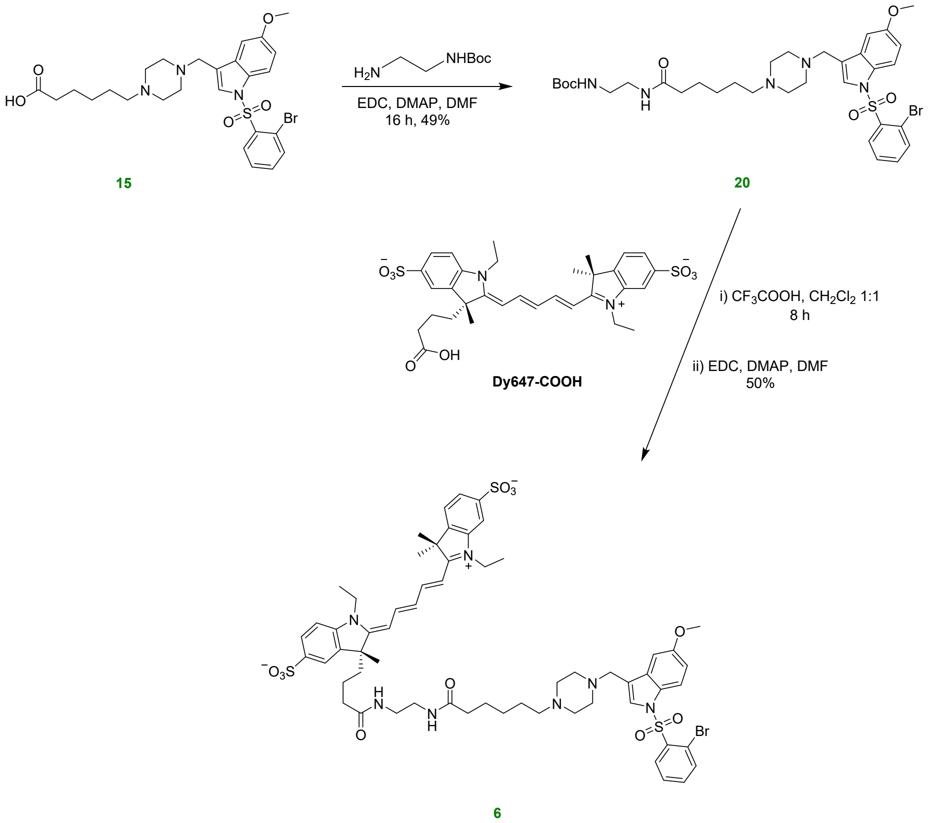

Scheme S5. Synthesis of compound **6**.

Compounds **3**,[9] **10–15**[10] and **Masupirdine**[10] were synthesised as reported.

***tert*-Butyl (2-(6-bromo-1,3-dioxo-1*H*-benzo[*de*]isoquinolin-2(3*H*)-yl)ethyl)carbamate (7)**

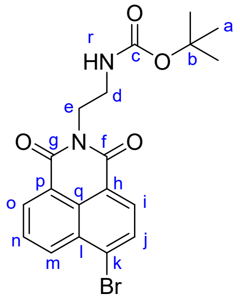

This compound was synthesised using a minor modification to the literature method;[11] 4-bromo-1,8-naphthalic anhydride (3.46 g, 0.0125 mmol 1 eq.) was suspended in 50 mL ethanol, *N*-Boc-ethylenediamine (1.98 mL, 0.0125, 1 eq.) was added and the reaction mixture was heated under reflux for 4 h. The solvent was evaporated under reduced pressure and the resulting crude was purified by silica gel column chromatography using isocratic elution in dichloromethane to afford the title compound **7** as a light brown solid (4.0 g, 73%); m.p. 170–175 °C; R_f = 0.41 (silica, dichloromethane); ^{1}H NMR (400 MHz, $CDCl_3$, 294 K) δ (ppm): 8.62 (d, *J* 7.5 Hz, 1H, H^o), 8.55 – 8.48 (d, *J* 8 Hz, 1H, H^m), 8.37 (d, *J* 8.0 Hz, 1H, H^i), 8.00 (d, *J* 8.0 Hz, 1H, H^j), 7.81 (dd, *J* 7.5, 8 Hz, 1H, H^n), 4.99 (br m, 1H, H^r), 4.33 (t, *J* 6 Hz, 2H, H^d), 3.52 (q, *J* 6 Hz, 2H, H^e), 1.27 (s, 9H, H^a); ^{13}C NMR (101 MHz, $CDCl_3$, 294 K) δ (ppm): 164.0 ($C^{g,f}$), 156.2 (C^c), 133.5 (C^m), 132.3 (C^o), 131.5 (C^i), 131.2 (C^j), 130.6 (C^q), 130.5 (C^l), 129.1 (C^k), 128.2 (C^n), 123.0 (C^h), 122.1 (C^p), 79.3 (C^b), 40.1 (C^d), 39.6 (C^e), 28.3 (C^a); HRMS (MALDI-TOF) *m/z*: $[M + Na]^+$ found, 443.0421. Calc. for $C_{19}H_{19}BrN_2NaO_4$, *M*r = 443.0402.

***tert*-Butyl (2-(1,3-dioxo-6-(propylamino)-1*H*-benzo[*de*]isoquinolin-2(3*H*)-yl)ethyl)carbamate (8)**

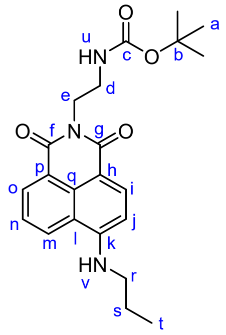

Procedure taken from the literature for a similar compound;[11] compound **7** (2 g, 4.77 mmol, 1 eq.) was added to 40 mL of 2-methoxyethanol. To this suspension, *n*-propylamine (5.9 mL, 71.55 mmol, 15 eq.) was added and the mixture was boiled under N_2 for 12 h. The mixture

was cooled to RT and evaporated to dryness to form an orange brown crude which was purified by column chromatography over silica gel using a gradient elution with dichloromethane/methanol (100:0 × 4 ⟶ 99:1 × 3 ⟶ 97:3 × 2 ⟶ 95:5 × 3, *v/v*) to afford the title compound **8**, as an orange solid (1.5 g, 79%); m.p. 90–95 °C; R_f = 0.37 (silica, dichloromethane/ethanol, 9:1); ^{1}H NMR (400 MHz, $CDCl_3$, 294 K) δ (ppm): 8.40 (d, *J* 7.0 Hz, 1H, H^{o}), 8.35 (d, *J* 8.5 Hz, 1H, H^{i}), 8.04 (d, *J* 8.0 Hz, 1H, H^{m}), 7.43 (t, *J* 8.0 Hz, 1H, H^{n}), 6.61 (d, *J* 8.0 Hz, 1H, H^{j}), 5.69 (t, *J* 5 Hz, 1H, H^{u}), 5.39-5.22 (m, 1H, H^{v}), 4.31 (dd, *J* 7.5, 6.0 Hz, 2H, H^{d}), 3.52 (m, 2H, H^{e}), 3.43 – 3.27 (m, 2H, H^{r}), 1.84 (p, *J* 7.5 Hz, 2H, H^{s}), 1.35 (s, 9H, H^{a}), 1.10 (t, *J* 7.5 Hz, 3H, H^{t}); ^{13}C NMR (101 MHz, $CDCl_3$, 294 K) δ (ppm): 165.1 ($C^{f,g}$), 164.5 (C^{c}), 156.3 (C^{l}), 150.0 (C^{q}), 131.2 (C^{i}), 129.8 (C^{o}), 126.3 (C^{m}), 124.4 (C^{n}), 122.5 (C^{p}), 120.1 (C^{h}), 109.3 (C^{k}), 104.2 (C^{j}), 79.2 (C^{b}), 45.5 (C^{r}), 40.3 (C^{e}), 39.4 (C^{d}), 28.4 (C^{a}), 22.2 (C^{s}), 11.7 (C^{t}); HRMS (MALDI-TOF) *m/z* (% relative intensity): $[M + Na]^+$ found, 420.1861 (100%). Calc. for $C_{22}H_{27}N_3O_4$, *M*r = 420.1891.

(2-Aminoethyl)-6-(propylamino)-1*H*-benzo[*de*]isoquinoline-1,3(2*H*)-dione (9)

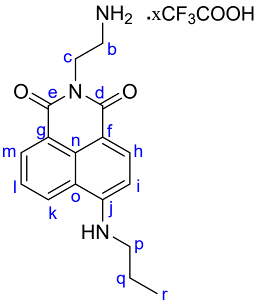

Procedure taken from the literature for a similar compound;[11] compound **8** (1 g, 4.77 mmol, 1 eq.) was added to dichloromethane (10 mL) and stirred. To this mixture, trifluoroacetic acid (3 mL, 71.55 mmol, 15 eq.) was added and the mixture was stirred for 12 h at RT. Solvent was removed under reduced pressure followed by the addition of methanol (100 mL × 6). Evaporation afforded the title compound **9**, as a red solid that was used directly in the next step (600 mg, 85%); m.p. 180–185 °C; R_f = 0.28 (silica, dichloromethane/methanol, 9.5:0.5); ^{1}H NMR (400 MHz, CD_3OD, 294 K) δ (ppm): 8.46 (dd, *J* 8.0, 9.0 Hz, 1H+1H, $H^{m,h}$), 8.29 (d, *J* 8.5 Hz, 1H, H^{k}), 7.57 (dd, *J* 9.0 8.0 Hz, 1H, H^{l}), 6.72 (d, *J* 9.0 Hz, 1H, H^{i}), 4.38 (t, *J* 6.0 Hz, 2H, H^{c}), 3.37 (t, *J* 7.5 Hz, 2H, H^{p}), 3.26 (m, 2H, H^{b}), 1.77 (h, *J* 7.5 Hz, 2H, H^{q}), 1.03 (t, *J* 7.5 Hz, 3H, H^{r}); ^{13}C NMR (101 MHz, CD_3OD, 294 K) δ (ppm): 166.8-166.0 ($C^{e,d}$), 153.1 (C^{f}), 136.3 (C^{k}), 132.4 (C^{g}), 131.6 (C^{n}), 129.7 (C^{h}), 125.4 (C^{m}), 123.2 (C^{l}), 121.8 (C^{o}), 108.7 (C^{j}), 105.1 (C^{i}), 46.2 (C^{p}), 40.3 (C^{c}), 38.7 (C^{b}), 22.7 (C^{q}), 11.9 (C^{r}); ^{19}F NMR (376 MHz, D_2O,

294 K) δ (ppm): -75.66 (CF_3COOH); HRMS (MALDI-TOF) *m/z* (% relative intensity): $[M]^+$ found, 297.1472 (100%). Calc. for $C_{17}H_{19}N_3O_2$, *M*r = 297.1477.

6-(4-((1-((2-Bromophenyl)sulfonyl)-5-methoxy-1*H*-indol-3-yl)methyl)piperazin-1-yl)-*N*-(2-(1,3-dioxo-6-(propylamino)-1*H*-benzo[*de*]isoquinolin-2(3*H*)-yl)ethyl)hexanamide (5)

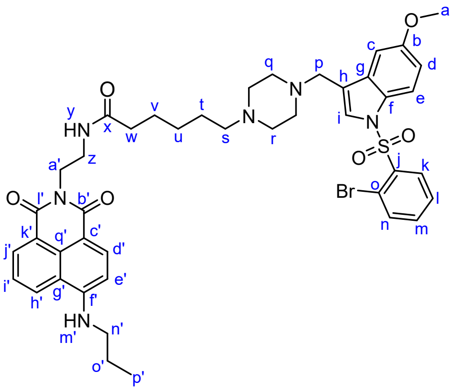

Compound **9** (25 mg, 0.068 mmol, 1 eq.) was dissolved in 1 mL of anhydrous DMF, followed by the addition of *N*,*N*-diisopropylethylamine (35.5 µL, 0.20 mmol, 3.0 eq.) and the mixture was stirred at RT. HATU (38.7 mg, 0.10 mmol, 1.5 eq.) was added and the solution was stirred for 15 min at RT. Compound **15**[10] (34 mg, 0.07 mmol, 1.05 eq.) was added to the mixture and stirred for 15 h at 50 °C. After cooling the reaction mixture to RT, brine (10 mL) was added, and the crude was extracted using ethyl acetate (10 mL × 7). The organic layers were combined and washed with 0.1 M HCl (10 mL × 3), water (5 mL), brine (5 mL) dried over anhydrous sodium sulphate, filtered, and the resulting filtrate was evaporated to dryness to afford a brown oil that was purified twice over flash column chromatography in dichloromethane/methanol (100:0 → 90:10, *v/v*, in 1% increment of methanol) to afford the title compound **5** as a pale orange solid (5 mg, 8%); ^{1}H NMR (600 MHz, $CDCl_3$, 294 K) δ (ppm): 8.57 (d, *J* 7.0 Hz, 1H, $H^{j'}$), 8.43 (d, *J* 8.0 Hz, 1H, $H^{d'}$), 8.32 (dd, *J* 8.0, 2.0 Hz, 1H, H^{n}), 8.16 (d, *J* 8.0 Hz, 1H, $H^{i'}$), 8.06 (br s, 1H, $H^{h'}$), 7.68 – 7.60 (m, 2H, $H^{e,l}$), 7.57 – 7.51 (m, 2H, $H^{k,e'}$), 7.45 (d, *J* 8.0 Hz, 1H, H^{c}), 7.11 (s, 1H, H^{i}), 6.96 – 6.88 (m, 2H, $H^{y,m}$), 6.73 (d, *J* 8.5 Hz, 1H, H^{d}), 4.46 (br s, 1H, H^{z}), 4.40 – 4.35 (t, *J* 5.0 Hz, 1H, $H^{y'}$), 3.80 (s, 3H, H^{a}), 3.69 – 3.52 (m, 8H, $H^{q,r}$), 3.38 (t, *J* 7.0 Hz, 2H, H^{w}), 2.92 (t, *J* 8.0 Hz, 2H, $H^{n'}$), 2.24 – 2.09 (m, 2H, H^{s}), 1.83 (h, *J* 7.0 Hz, 2H, $H^{o'}$), 1.60 (m, 4H, $H^{a',p}$), 1.38 – 1.17 (m, 6H, $H^{v,u,t}$), 1.09 (t, *J* 7.0 Hz, 3H, $H^{p'}$).; ^{13}C NMR (151 MHz, $CDCl_3$, 294 K) δ (ppm): 174.7 (C^{x}), 165.4 ($C^{l'}$), 165.1 ($C^{b'}$), 161.0 ($C^{c'}$), 157.5 (C^{b}), 150.4 (C^{j}), 143.4 ($C^{d'}$), 137.2 ($C^{j'}$), 137.1 ($C^{h'}$), 136.3 (C^{m}), 135.4 (C^{k}), 132.3 (C^{l}), 131.9 (C^{n}), 128.2 (C^{f}), 126.5 (C^{d}), 125.0 (C^{g}), 129.0 ($C^{q'}$), 122.7 ($C^{k'}$), 122.4 ($C^{i'}$), 121.0 ($C^{j'}$), 120.8 (C^{c}), 120.3 ($C^{d'}$), 116.4 (C^{e}), 115.7 (C^{o}), 114.5 (C^{h}), 112.6 ($C^{q'}$), 109.3 (C^{f}),

104.7 ($C^{e'}$), 100.8 (C^{i}), 57.0 (C^{q}), 55.7 (C^{a}), 51.0 (C^{r}), 48.8 (C^{p}), 47.9 (C^{w}), 45.6 ($C^{n'}$), 40.2 ($C^{a'}$), 39.0 (C^{z}), 35.4 (C^{s}), 29.9 (C^{u}), 25.3 (C^{t}), 24.3 (C^{v}), 22.3 ($C^{o'}$), 11.7 ($C^{p'}$); HRMS (MALDI-TOF) *m/z* (% relative intensity): $[M + H]^+$ found, 859.5570 (100%). Calc. for $C_{43}H_{50}BrN_6O_6S$, *M*r = 859.2678.

2-Chloro-*N*-(4-chloro-3-(5-(3-((2-(1,3-dioxo-6-(propylamino)-1*H*-benzo[*de*]isoquinolin-2(3*H*)-yl)ethyl)amino)-3-oxopropyl)pyridin-2-yl)phenyl)-4-(methylsulfonyl)benzamide (1)

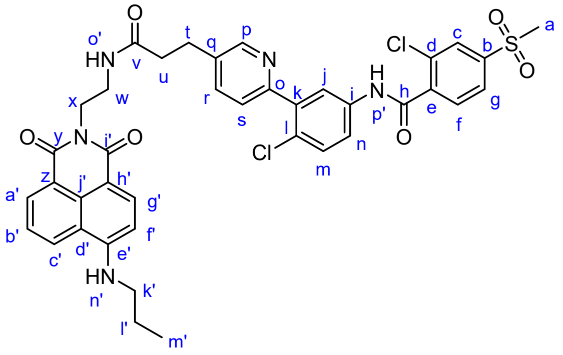

Compound **9** (25 mg, 0.050 mmol, 1 eq.) was dissolved in anhydrous DMF (2 mL), followed by the addition of *N*,*N*-diisopropylethylamine (26.5 µL, 0.152 mmol, 3.0 eq.) and the mixture was stirred at RT. HATU (28.9 mg, 0.076 mmol, 1.5 eq.) was added and the solution was stirred for 15 min at RT. 3-(6-(2-Chloro-5-(2-chloro-4-(methylsulfonyl)benzamido)phenyl)pyridin-3-yl)propanoic acid (15.8 mg, 0.053 mmol, 1.05 eq.) was added to the mixture and stirred for 15 h at 50 °C. After cooling the reaction mixture to RT, brine (10mL) was added, and the crude was extracted using ethyl acetate (10 mL × 7). The organic layers were combined and washed with 0.1 M HCl (10 mL × 3), water (5 mL), brine (5 mL), dried over anhydrous sodium sulphate, filtered, and the resulting filtrate was evaporated to dryness to afford a yellow oil that was purified by column chromatography over silica gel using gradient elution with chloroform/isopropanol (100:0 → 90:10, *v/v*, in 1% increment of isopropanol). The resulting crude was purified over preparative TLC in 90:10 chloroform/isopropanol *v/v*, to afford the title compound as a yellow solid (12 mg, 18%); m.p. 193–195 °C; R_f = 0.35 (silica, chloroform/isopropanol, 9.5:0.5); ^{1}H NMR (600 MHz, $CDCl_3$, 296 K) δ 10.25 (s, 1H, $H^{p'}$), 8.40 (d, *J* 7.0 Hz, 1H, H^{p}), 8.31 (d, *J* 8.5 Hz, 1H, $H^{g'}$), 8.15 (dd, *J* 9.0, 2.5 Hz, 1H, $H^{a'}$), 8.00 (d, *J* 7.0 Hz,1H, $H^{b'}$), 7.94 (d, *J* 8.0 Hz, 1H, H^{m}), 7.81 (d, *J* 8.0 Hz, 1H, H^{g}), 7.62 (dd, *J* 8.5, 8.0 Hz, 1H, H^{j}), 7.50 – 7.33 (m, 5H, $H^{n,s,r,f,c}$), 7.22 (d, *J* 8.0 Hz, 1H, $H^{c'}$), 6.74 (t, *J* 5.0 Hz, 1H, $H^{o'}$), 6.61 (d, *J* 8.5 Hz, 1H, $H^{f'}$), 5.38 (t, *J* 5.0 Hz, 1H, $H^{n'}$), 4.27 – 4.19 (m, 2H, H^{x}), 3.54 (dt, *J* 5.0 Hz, 2H, H^{w}), 3.33 (dt, *J* 7.0, 5.0 Hz, 2H, $H^{k'}$), 2.89 (s, 3H, H^{a}), 2.79 (t, *J* 7.5 Hz, 2H, H^{t}), 2.38 (t, *J* 7.5 Hz, 2H, H^{u}), 1.84 (p, *J* 7.5 Hz, 2H, $H^{l'}$), 1.15 –

1.08 (t, *J* 7.5 Hz, 3H, $H^{m'}$); ^{13}C NMR (151 MHz, $CDCl_3$, 296 K) δ 171.7 (C^{v}), 165.2 (C^{y}), 164.8 ($C^{i'}$), 164.1 (C^{h}), 153.2 (C^{o}), 150.0 ($C^{e'}$), 148.7 (C^{b}), 142.6 (C^{j}), 140.9 (C^{i}), 138.2 (C^{k}), 137.4 (C^{e}), 136.1 (C^{d}), 135.9 (C^{p}), 134.8 (C^{q}), 132.3 (C^{r}), 131.3 (C^{s}), 130.9 (C^{f}), 130.0 (C^{l}), 129.8 ($C^{j'}$), 128.8 ($C^{h'}$), 127.1 (C^{c}), 126.4 (C^{n}), 125.9 (C^{m}), 125.0 ($C^{b'}$), 124.6 ($C^{c'}$), 122.9 (C^{s}), 122.6 (C^{n}), 121.6 (C^{g}), 120.1 ($C^{a'}$), 109.4 ($C^{g'}$), 104.4 ($C^{f'}$), 45.6 ($C^{k'}$), 44.3 (C^{a}), 39.6 (C^{w}), 39.2 (C^{x}), 37.1 (C^{u}), 28.0 (C^{t}), 22.4 ($C^{l'}$), 11.8 ($C^{m'}$); HRMS (MALDI-TOF) *m/z* (% relative intensity): $[M]^+$ found, 772.1736 (100%). Calc. for $C_{39}H_{35}Cl_2N_5O_6S$, *M*r = 772.1758.

Ethyl 6-(6-bromo-1,3-dioxo-1*H*-benzo[*de*]isoquinolin-2(3*H*)-yl)hexanoate (16)

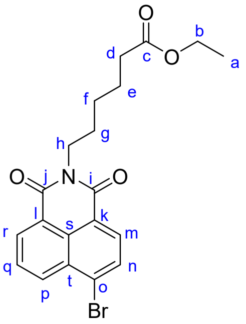

4-Bromo-1,8-naphthalic anhydride (500 mg, 1.804 mmol 1 eq.) was suspended in 50 mL ethanol, 6-aminohexanoic acid (289.3 mg, 1.804 mmol, 1 eq.) was added and the reaction mixture was heated under reflux for 4 h. After colling to RT, the reaction mixture was filtered, and the filtrate was cooled to 0 °C. Oxalyl chloride (183 µL, 2.165 mmol, 1.2 eq.) was added dropwise to this solution and left to warm to RT while stirring. After 16 h, the solvent was removed and the residue was purified by column chromatography over silica gel using a gradient elution with dichloromethane/methanol (100:0 × 2 → 99:1 → 98:2 → 97:3, *v/v*) to afford the title compound **16**, as a pale yellow solid (151 mg, 20%); m.p. 178–180 °C; R_f = 0.25 (silica, dichloromethane/hexane, 1:1); ^{1}H NMR (400 MHz, $CDCl_3$, 294 K) δ (ppm): 8.49 (dd, *J* 7.0, 1.0 Hz, 1H, H^{r}), 8.37 (d, *J* 8.5 Hz, 1H, H^{p}), 8.23 (d, *J* 8.0 Hz, 1H, H^{m}), 7.88 (d, *J* 8.0 Hz, 1H, H^{n}), 7.71 (t, *J* 8.0 Hz, 1H, H^{q}), 4.07 (q, *J* 7.0 Hz, 2H + 2H, $H^{h,b}$), 2.27 (t, *J* 7.5 Hz, 2H, H^{d}), 1.68 (m, 2H + 2H, $H^{e,g}$), 1.50 – 1.34 (m, 2H, H^{f}), 1.20 (t, *J* 7.0 Hz, 3H, H^{a}); ^{13}C NMR (101 MHz, $CDCl_3$, 294 K) δ (ppm): 173.6 (C^{c}), 163.4 (C^{i}), 163.3 (C^{j}), 133.0 (C^{p}), 131.9 (C^{r}), 131.0 (C^{m}), 130.9 (C^{n}), 130.3 (C^{k}), 130.1 (C^{l}), 128.7 (C^{o}), 128.0 (C^{q}), 122.9 (C^{t}), 122.0 (C^{s}), 60.2 (C^{b}), 40.3 (C^{h}), 34.2 (C^{d}), 27.7 (C^{g}), 26.6 (C^{f}), 24.7 (C^{e}), 14.3 (C^{a}); HRMS (MALDI-TOF) *m/z* (% relative intensity): $[M + Na]^+$ found, 442.0463 (100%). Calc. for $C_{20}H_{20}BrNO_4$, *M*r = 442.0450.

Ethyl 6-(1,3-dioxo-6-(propylamino)-1*H*-benzo[*de*]isoquinolin-2(3*H*)-yl)hexanoate (17)

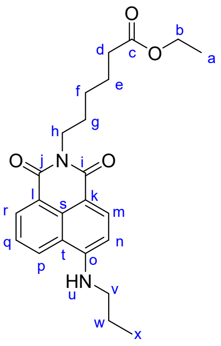

Compound **16** (150 mg, 0.35 mmol, 1 eq.) was added to 2 mL of 2-methoxyethanol. To this solution, *n*-propylamine (442 µL, 5.38 mmol, 15 eq.) was added upon stirring and the mixture was refluxed under N_2 for 12 h. The mixture was cooled to RT and evaporated to dryness to form an orange-brown crude which was passed through a silica plug in dichloromethane. The resulting crude was purified over preparative TLC in 90:10 dichloromethane/diethyl ether *v/v*, to afford the title compound **17**, as a yellow solid (130 mg, 90%); m.p. 220–225 °C; R_f = 0.27 (silica, dichloromethane/diethyl ether, 9:1); ^{1}H NMR (400 MHz, $CDCl_3$, 294 K) δ (ppm): 8.52 (dd, *J* 7.0, 1.0 Hz, 1H, H^r), 8.41 (d, *J* 8.0 Hz, 1H, H^m), 8.09 (d, *J* 8.0 Hz, 1H, H^p), 7.55 (t, *J* 7.0 Hz, 1H, H^q), 6.67 (d, *J* 8.5 Hz, 1H, H^n), 5.42 (t, *J* 8.0 Hz, 1H, H^u), 4.24 – 3.98 (m, 2H+2H, $H^{h,b}$), 3.35 (q, *J* 7.0 Hz, 2H, H^v), 2.29 (t, *J* = 8.0 Hz, 2H, H^d), 1.91 – 1.57 (m, 2H+2H+2H. $H^{w,e,g}$), 1.50 – 1.36 (p, *J* 8.0 Hz, 2H, H^f), 1.22 (t, *J* 7.0 Hz, 3H, H^a), 1.09 (t, *J* 7.0 Hz, 3H, H^x); ^{13}C NMR (101 MHz, $CDCl_3$, 294 K) δ (ppm): 173.9 (C^c), 164.8 (C^j), 164.2 (C^i), 149.6 (C^o), 134.6 (C^m), 131.2 (C^r), 129.9 (C^l), 126.0 (C^p), 124.7 (C^q), 123.1 (C^k), 120.2 (C^t), 110.1 (C^s), 104.4 (C^n), 60.3 (C^b), 45.5 (C^v), 40.0 (C^h), 34.4 (C^d), 27.9 (C^e), 26.8 (C^f), 24.9 (C^g), 22.3 (C^w), 14.3 (C^a), 11.8 (C^x); HRMS (MALDI-TOF) *m/z* (% relative intensity): $[M]^+$ found, 396.2065 (100%). Calc. for $C_{23}H_{28}N_2O_4$, *M*r = 396.2043.

6-(1,3-Dioxo-6-(propylamino)-1*H*-benzo[*de*]isoquinolin-2(3*H*)-yl)hexanoic acid (18)

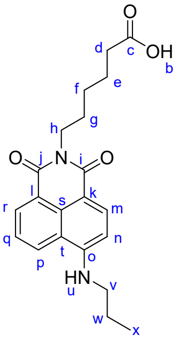

Compound **17** (130 mg, 0.33 mmol) was dissolved in tetrahydrofuran (6 mL). 1M potassium hydroxide (3 mL) was added and stirred at RT for 12 h. The solvents were removed under reduced pressure to form an oily residue which was neutralised by the dropwise addition of 1M HCl to form a precipitate which was isolated by centrifugation and dried in vacuum to afford the title compound **18,** as an orange solid (50 mg, 41%); m.p. 180–185 °C; ^{1}H NMR (600 MHz, DMSO-d_6, 296 K) δ (ppm): 11.97 (br s, 1H, H^{b}), 8.72 (d, *J* 8.0 Hz, 1H, H^{r}), 8.39 (d, *J* 7.0 Hz, 1H, H^{p}), 8.22 (d, *J* 8.5 Hz, 1H, H^{m}), 7.85 (t, *J* 5.5 Hz, 1H, H^{u}), 7.63 (t, *J* 8.0 Hz, 1H, H^{q}), 6.73 (d, *J* 8.5 Hz, 1H, H^{n}), 3.97 (t, *J* 7.5 Hz, 2H, H^{h}), 3.32 (q, *J* 6.5 Hz, 2H, H^{v}), 2.20 (t, *J* 7.0 Hz, 2H, H^{d}), 1.70 (h, *J* 7.0 Hz, 2H, H^{w}), 1.55 (m, 2H+2H, $H^{g,e}$), 1.31 (p, *J* 8.0 Hz, 2H, H^{f}), 0.97 (t, *J* = 7.0 Hz, 3H, H^{x}); ^{13}C NMR (151 MHz, DMSO-d_6, 296 K) δ (ppm): 174.5 (C^{c}), 163.8 (C^{j}), 162.9 (C^{i}), 150.8 (C^{o}), 134.3 (C^{m}), 130.7 (C^{p}), 129.5 (C^{r}), 128.7 (C^{k}), 124.2 (C^{q}), 121.8 (C^{s}), 120.1 (C^{l}), 107.4 (C^{t}), 103.8 (C^{n}), 79.3 (C^{h}), 44.6 (C^{v}), 33.6 (C^{d}), 27.5 (C^{g}), 26.1 (C^{f}), 24.3 (C^{e}), 21.2 (C^{w}), 11.6 (C^{x}); HRMS (MALDI-TOF) *m/z* (% relative intensity): $[M]^{+}$ found, 368.9935 (100%). Calc. for $C_{21}H_{24}N_2O_4$, *M*r = 368.1730.

2-Chloro-*N*-(4-chloro-3-(5-(3-(6-(1,3-dioxo-6-(propylamino)-1*H*-benzo[*de*]isoquinolin-2(3*H*)-yl)hexanamido)propyl)pyridin-2-yl)phenyl)-4-(methylsulfonyl)benzamide (2)

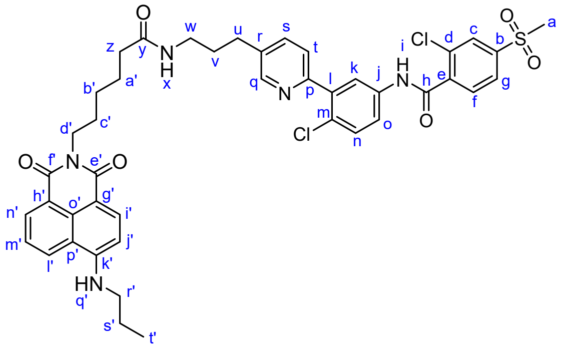

Compound **18** (25 mg, 0.068 mmol, 1 eq.) was dissolved in 1 mL of anhydrous DMF, followed by the addition of *N*,*N*-diisopropylethylamine (35.5 µL, 0.20 mmol, 3.0 eq.) and the mixture was stirred at RT. HATU (38.7 mg, 0.10 mmol, 1.5 eq.) was added and the solution was stirred for 15 min at RT. *N*-(3-(5-(3-aminopropyl)pyridin-2-yl)-4-chlorophenyl)-2-chloro-4-(methylsulfonyl)benzamide (34 mg, 0.07 mmol, 1.05 eq.) was added to the mixture and stirred for 15 h at 50 °C. After cooling the reaction mixture to RT, brine (10mL) was added, and the crude was extracted using ethyl acetate (10 mL × 7). The organic layers were combined and washed with 0.1 M HCl (10 mL × 3), water (5 mL), brine (5 mL), dried over anhydrous sodium sulfate, filtered, and the resulting filtrate was evaporated to dryness to afford a brown oil that was purified over preparative TLC in 95:5 chloroform/isopropanol *v/v*,

to afford the title compound **2**, as an orange solid (15 mg, 56%); m.p. 130 – 135 °C; R_f = 0.30 (silica, chloroform/isopropanol, 9.5:0.5); ^{1}H NMR (400 MHz, $CDCl_3$, 294 K) δ 10.48 (s, 1H, H^{i}), 8.49 (dd, *J* 7.0, 1.0 Hz, 1H, H^{q}), 8.37 (d, *J* 8.0 Hz, 1H, $H^{n'}$), 8.19 – 8.07 (dd, *J* 8.5, 1 Hz, 1H, $H^{l'}$), 8.01 (br m, 1H, $H^{m'}$), 7.97 (dd, *J* 8.5, 2.5 Hz, 1H, H^{g}), 7.84 (d, *J* 1.5 Hz, 1H, H^{f}), 7.72 – 7.63 (m, 1H+1H, $H^{o,s}$), 7.61 – 7.46 (m, 1H+1H+1H+1H, $H^{k,c,n,t}$), 7.41 (d, *J* 8.5 Hz, 1H, H^{n}), 6.68 (d, *J* 8.5 Hz, 1H, $H^{j'}$), 6.19 (t, *J* 5.5 Hz, 1H, $H^{q'}$), 5.56 (t, *J* 5.0 Hz, 1H, H^{x}), 4.11 (t, *J* 7.0 Hz, 2H, $H^{d'}$), 3.36 (q, *J* 7.0 Hz, 2H, $H^{r'}$), 3.24 (q, *J* 6.5 Hz, 2H, H^{w}), 3.01 (s, 3H, H^{a}), 2.53 (t, *J* 7.5 Hz, 2H, H^{u}), 2.18 (t, *J* 7.5 Hz, 2H, H^{z}), 1.83 (h, *J* 7.0 Hz, 2H, $H^{s'}$), 1.77 – 1.58 (m, 2H+2H, $H^{v+e'}$), 1.46 – 1.35 (m, 2H, $H^{b'}$), 1.29 (m, 2H, $H^{a'}$), 1.10 (t, *J* 7.0 Hz, 3H, $H^{t'}$); ^{13}C NMR (151 MHz, $CDCl_3$, 296 K) δ 173.5 (C^{y}), 164.8 (C^{h}), 164.2 ($C^{f'}$), 164.1 ($C^{e'}$), 153.6 ($C^{k'}$), 149.9 (C^{p}), 148.6 ($C^{m'}$), 142.5 (C^{b}), 141.2 (C^{j}), 138.5 (C^{l}), 137.2 (C^{r}), 136.5 (C^{e}), 136.4 (C^{d}), 134.6 ($C^{n'}$), 132.5 (C^{m}), 131.2 ($C^{o'}$), 130.9 (C^{n}), 130.0 (C^{q}), 129.9 (C^{c}), 128.8 (C^{f}), 127.4 ($C^{h'}$), 126.3 ($C^{l'}$), 125.8 (C^{s}), 125.2 (C^{t}), 124.7 ($C^{i'}$), 123.2 (C^{o}), 123.0 ($C^{p'}$), 121.8 (C^{g}), 120.3 ($C^{g'}$), 109.9 (C^{k}), 104.4 ($C^{j'}$), 45.6 ($C^{r'}$), 44.4 (C^{a}), 39.9 ($C^{d'}$), 39.0 (C^{v}), 36.5 (C^{z}), 30.8 (C^{u}), 30.1 ($C^{a'}$), 27.8 ($C^{e'}$), 26.6 ($C^{b'}$), 25.5 ($C^{e'}$), 22.3 ($C^{s'}$), 11.8 ($C^{t'}$); HRMS (MALDI-TOF) *m/z* (% relative intensity): $[M + H]^+$ found, 828.2361 (100%). Calc. for $C_{43}H_{43}Cl_2N_5O_6S$, *M*r = 828.2384.

5-(4-((5-((*tert*-Butoxycarbonyl)amino)pentyl)carbamoyl)phenyl)-2,3,3,7,7,8-hexamethyl-2,3,7,8-tetrahydro-1*H*-pyrano[3,2-*f*:5,6-*f'*]diindol-9-ium-10,12-disulfonate (19)

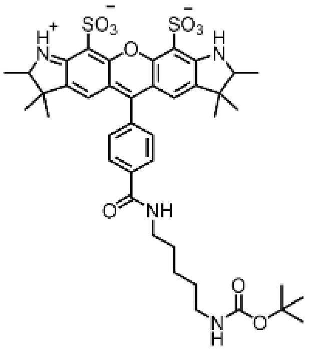

To a solution of *tert*-butyl (5-aminopentyl)carbamate (0.35 µL, 1.66 µmol) in DMF (200 µL) was added the **AF-532-NHS ester** (0.95 mg, 1.38 µmol). The mixture was stirred at room temperature for 4 h. Upon completion, the crude was purified by preparative HPLC (*Method L, t_R:* 16.7 min) to afford the title product **19** as a pink solid (0.68 mg, 64%). *Method A,* t_R: 15.0 min. Purity: 99.2%. **HRMS (MALDI)** calc. for $[C_{40}H_{49}NaN_4O_{10}S_2]^+$ $[M+Na]^+$ 833.2861, found 833.2834.

5-(4-((5-(3-(6-(2-Chloro-5-(2-chloro-4-(methylsulfonyl)benzamido)phenyl)pyridin-3-yl)propanamido)pentyl)carbamoyl)phenyl)-2,3,3,7,7,8-hexamethyl-2,3,7,8-tetrahydro-1*H*-pyrano[3,2-*f*:5,6-*f'*]diindol-9-ium-10,12-disulfonate (4)

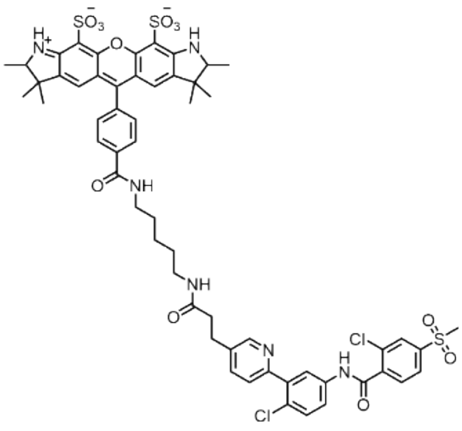

To a solution of compound **19** (2.70 mg, 3.28 µmol) in dichloromethane (0.6 mL) was added trifluoroacetic acid (0.2 mL). The mixture was stirred at room temperature for 16 h. Upon completion, dichloromethane (3 mL) was added, and the solvent was removed under reduced pressure. The addition of dichloromethane was repeated twice to remove any residual TFA. The crude was redissolved in DMF (500 µL), to which was added 3-(6-(2-chloro-5-(2-chloro-4-(methylsulfonyl)benzamido)phenyl)pyridin-3-yl)propanoic acid (1.94 mg, 3.94 µmol), EDC (611 µg, 3.94 µmol) and DMAP (481 µg, 3.94 µmol). Upon completion, the crude was purified by preparative HPLC (*Method H, **t_R**:* 26.6 min) to afford a pink solid **4** (1.95 mg, 50%). *Method A,* **t_R**: 12.0 min. Purity: 99.4%. **HRMS (MALDI)** calc. for [$C_{57}H_{59}Cl_2N_6O_{12}S_3$] $[M+H]^+$ 1187.272, found 1187.275. λ_{max} = 528 nm; ε = 56,000 M^{-1} cm^{-1}.

***tert*-Butyl (2-(6-(4-((1-((2-bromophenyl)sulfonyl)-5-methoxy-1*H*-indol-3-yl)methyl)piperazin-1-yl)hexanamido)ethyl)carbamate (20)**

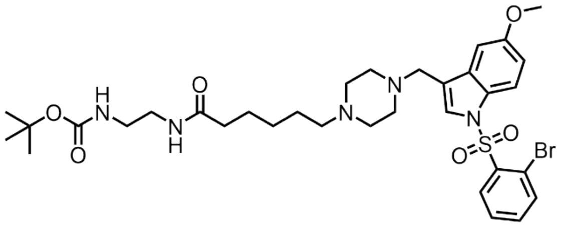

To a solution of compound **15**[10] (5.0 mg, 8.67 µmol) in DMF (200 µL) was added *N*-Boc-ethylenediamine (1.4 mg, 8.7 µmol), EDC (1.6 mg, 10.4 µmol) and DMAP (1.3 mg, 10.4 µmol). The mixture was stirred at room temperature for 16 h. Upon completion, the crude was purified by preparative HPLC (*Method D, **t_R**:* 16.0 min) to afford the title product

20 as a pale-yellow solid (3.1 mg, 49%). *Method A,* t_R: 13.6 min. Purity: 99.7%. **HRMS (MALDI)** calc. for $[C_{33}H_{46}BrN_5O_6S]^+$ $[M]^+$ 721.2331, found 721.2353.

2-((1*E*,3*E*)-5-((*R*,*Z*)-3-(4-((2-(6-(4-((1-((2-Bromophenyl)sulfonyl)-5-methoxy-1*H*-indol-3-yl)methyl)piperazin-1-yl)hexanamido)ethyl)amino)-4-oxobutyl)-1-ethyl-3-methyl-5-sulfonatoindolin-2-ylidene)penta-1,3-dien-1-yl)-1-ethyl-3,3-dimethyl-3*H*-indol-1-ium-6-sulfonate (6)

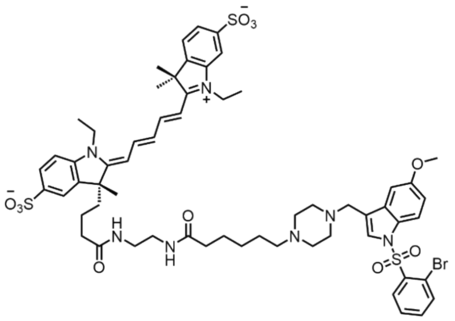

To a solution of compound **20** (3.1 mg, 4.2 µmol) in dichloromethane (0.5 mL) was added trifluoroacetic acid (0.5 mL). The mixture was stirred at room temperature for 16 h. Upon completion, dichloromethane (5 mL) was added to dilute the solution, and the solvent was removed under reduced pressure. The rinsing was repeated twice to remove the TFA residue. The crude protonated amine was then redissolved in dimethylformamide (500 µL) and was added the cyanine acid **Dy647-COOH** (3.3 mg, 5.0 µmol), EDC (780 µg, 5.0 µmol) and DMAP (610 µg, 5.0 µmol). The mixture was stirred at room temperature for 16 h. Upon completion, the crude was purified by preparative HPLC (*Method C,* t_R*:* 30.1 min) to afford the title product **6** as a blue solid (2.2 mg, 43%). *Method A,* t_R: 12.3 min. Purity: 98.5%. **HRMS (MALDI)** calc. for $[C_{60}H_{73}BrN_7O_{11}S_3]^-$ $[M]^-$ 1244.371, found 1244.375. λ_{max} 650 nm; ε 131,000 M^{-1} cm^{-1}.

7. Selected High-Resolution Mass and NMR spectra

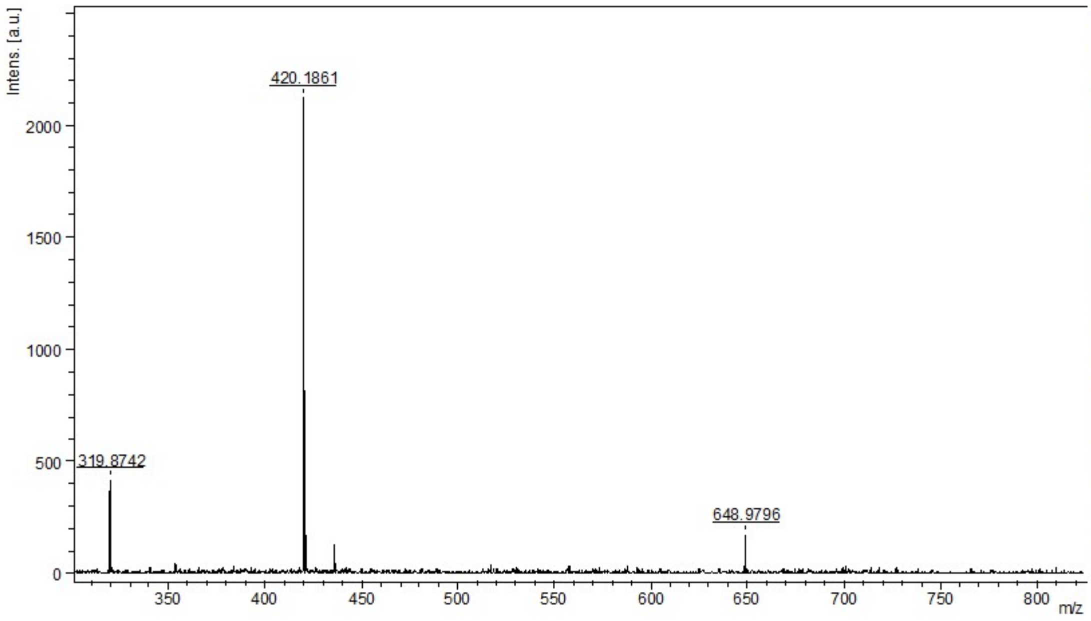

Figure S4. MALDI-TOF mass spectrum of compound **8**.

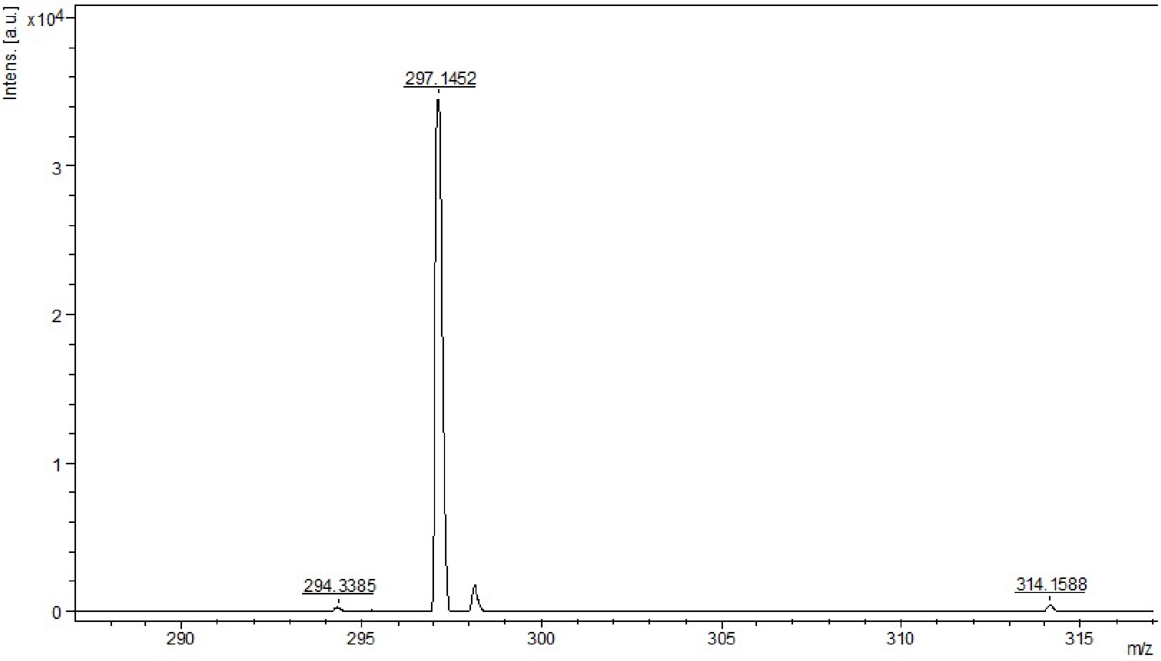

Figure S5. MALDI-TOF mass spectrum of compound **9**.

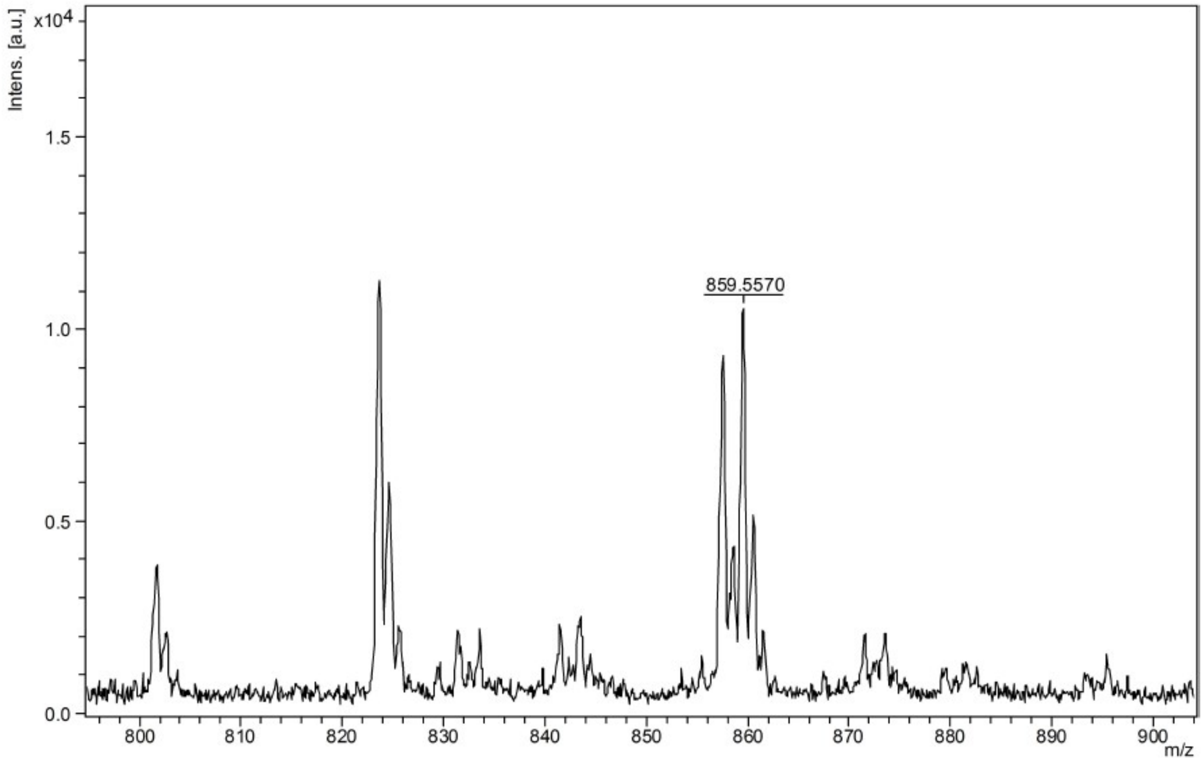

Figure S6. MALDI-TOF mass spectrum of compound **5**.

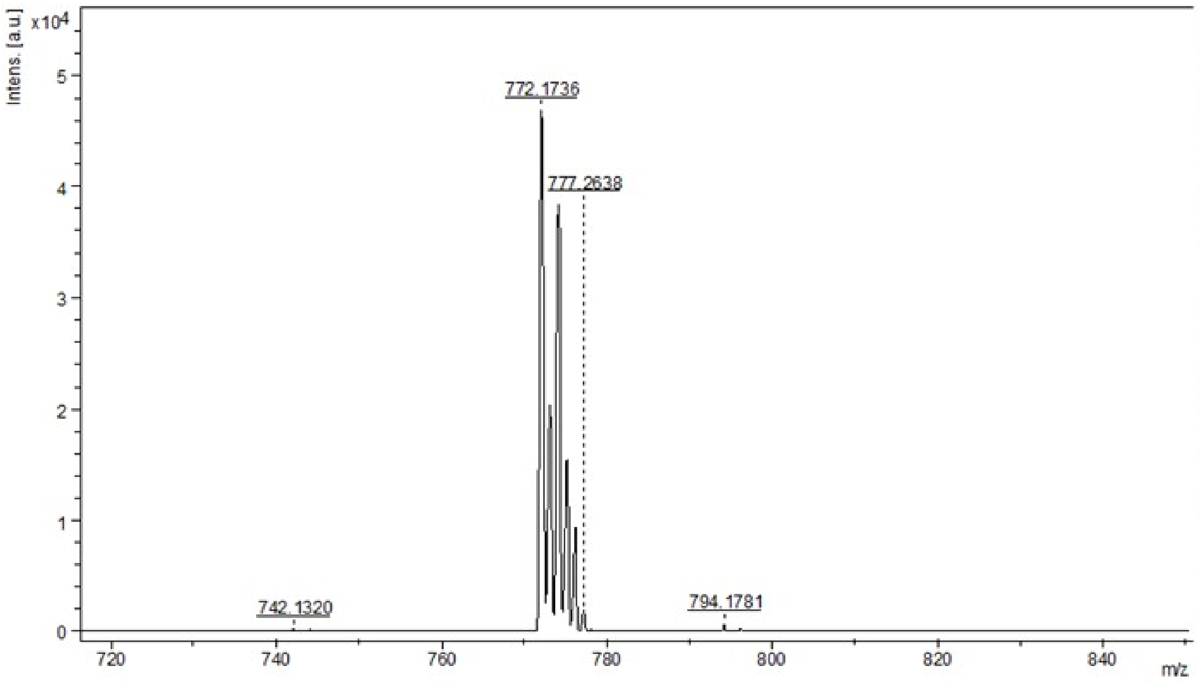

Figure S7. MALDI-TOF mass spectrum of compound **1**.

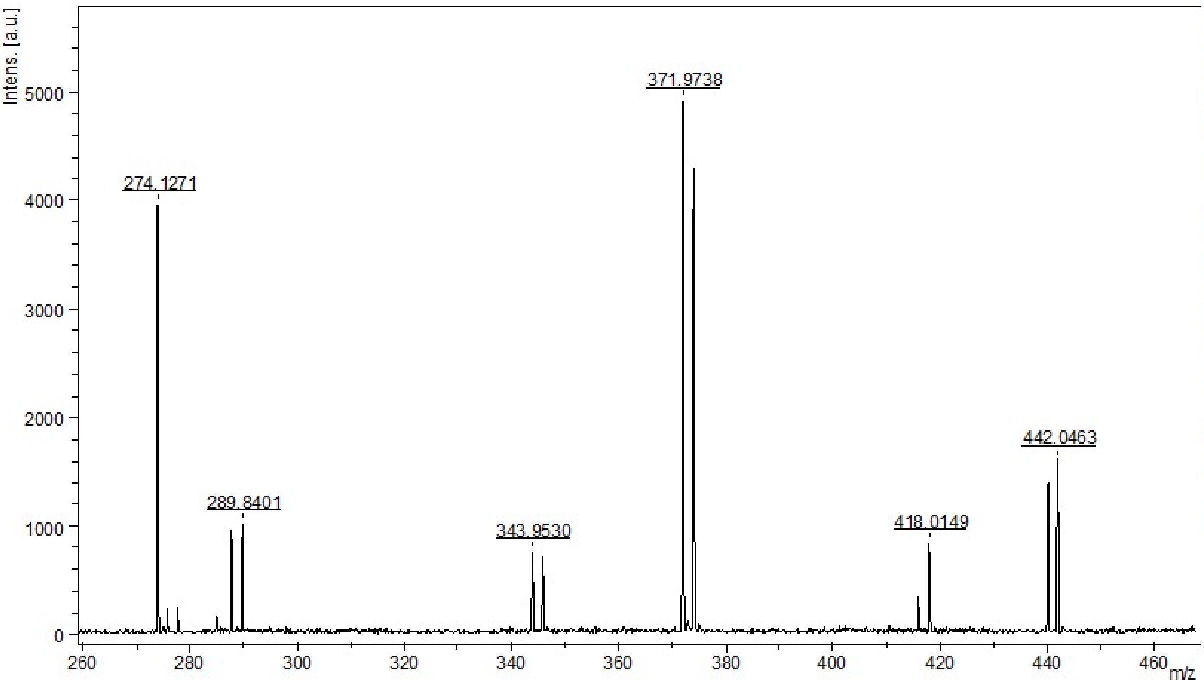

Figure S8. MALDI-TOF mass spectrum of compound **16**.

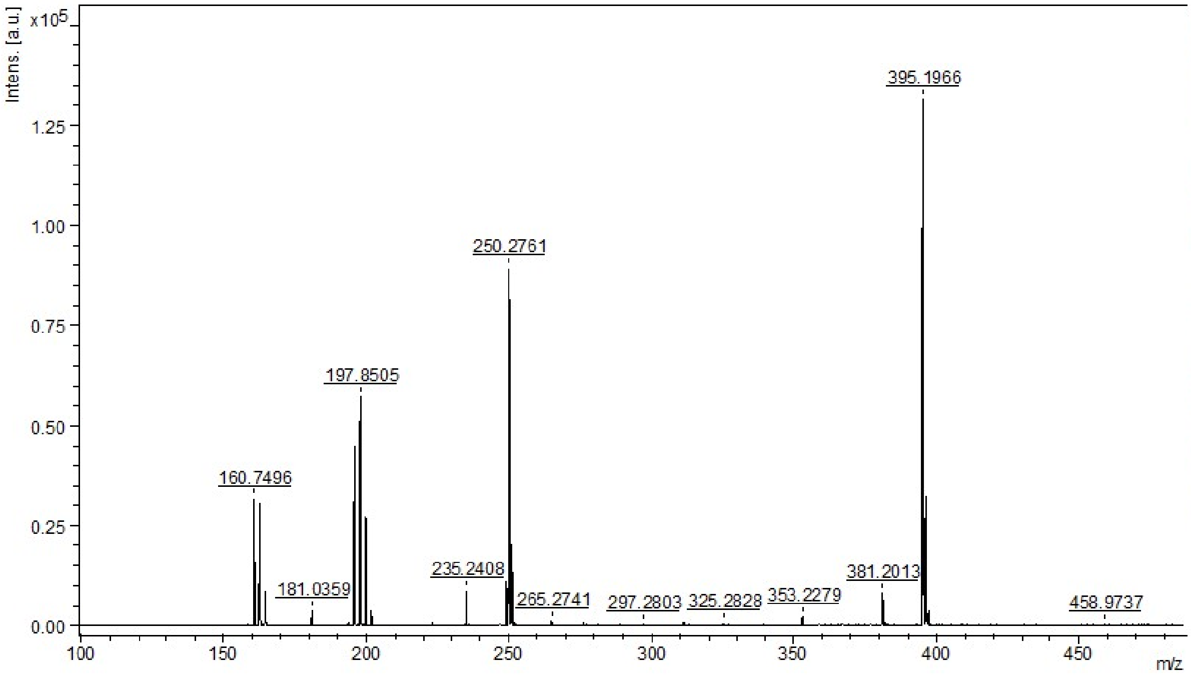

Figure S9. MALDI-TOF mass spectrum of compound **17**.

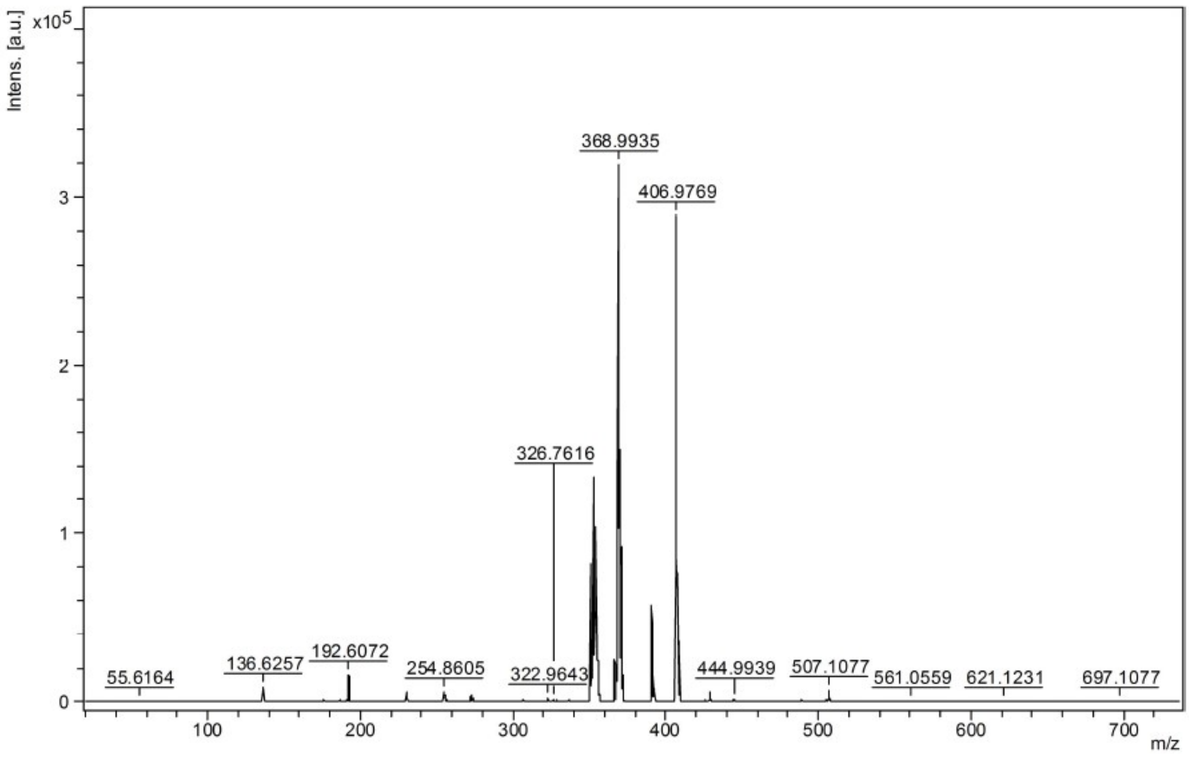

Figure S10. MALDI-TOF mass spectrum of compound **18**.

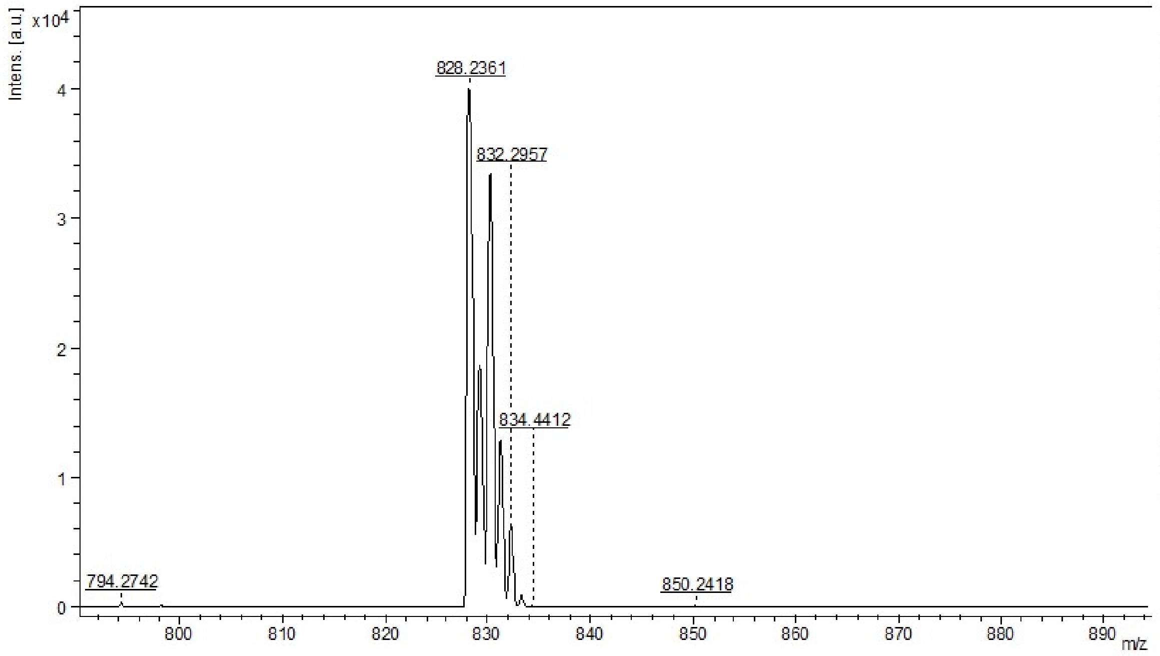

Figure S11. MALDI-TOF mass spectrum of compound **2**.

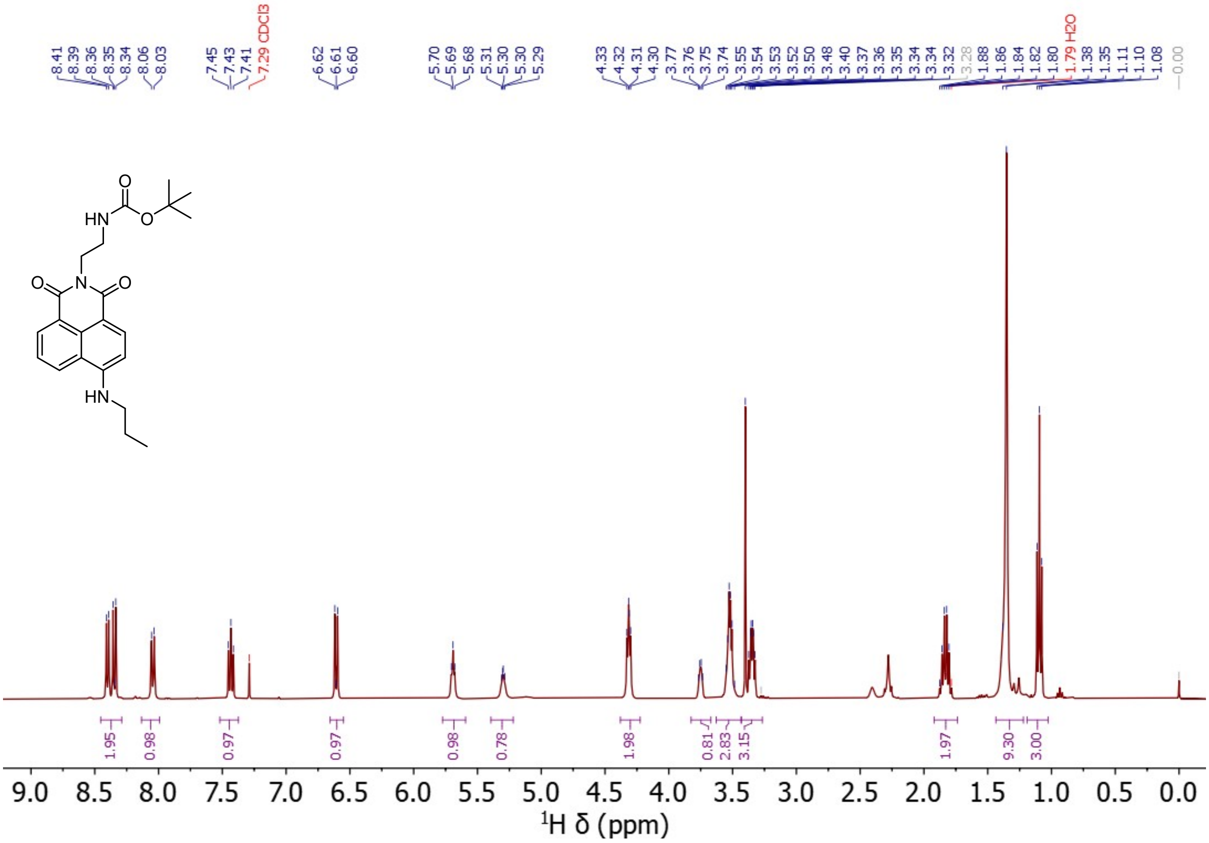

Figure S12. 400 MHz ^{1}H NMR spectrum of compound **8** in $CDCl_3$ at 294 K.

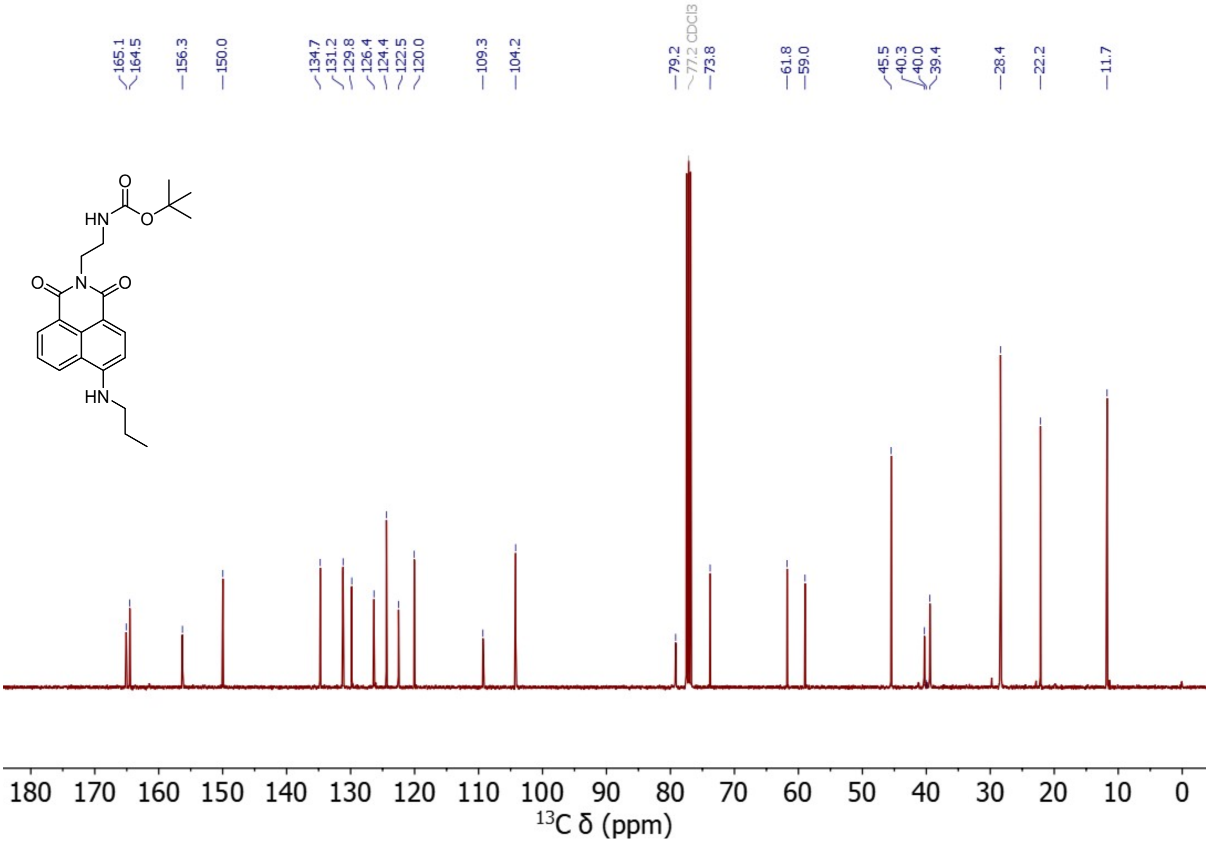

Figure S13. 101 MHz ^{13}C NMR spectrum of compound **8** in $CDCl_3$ at 294 K.

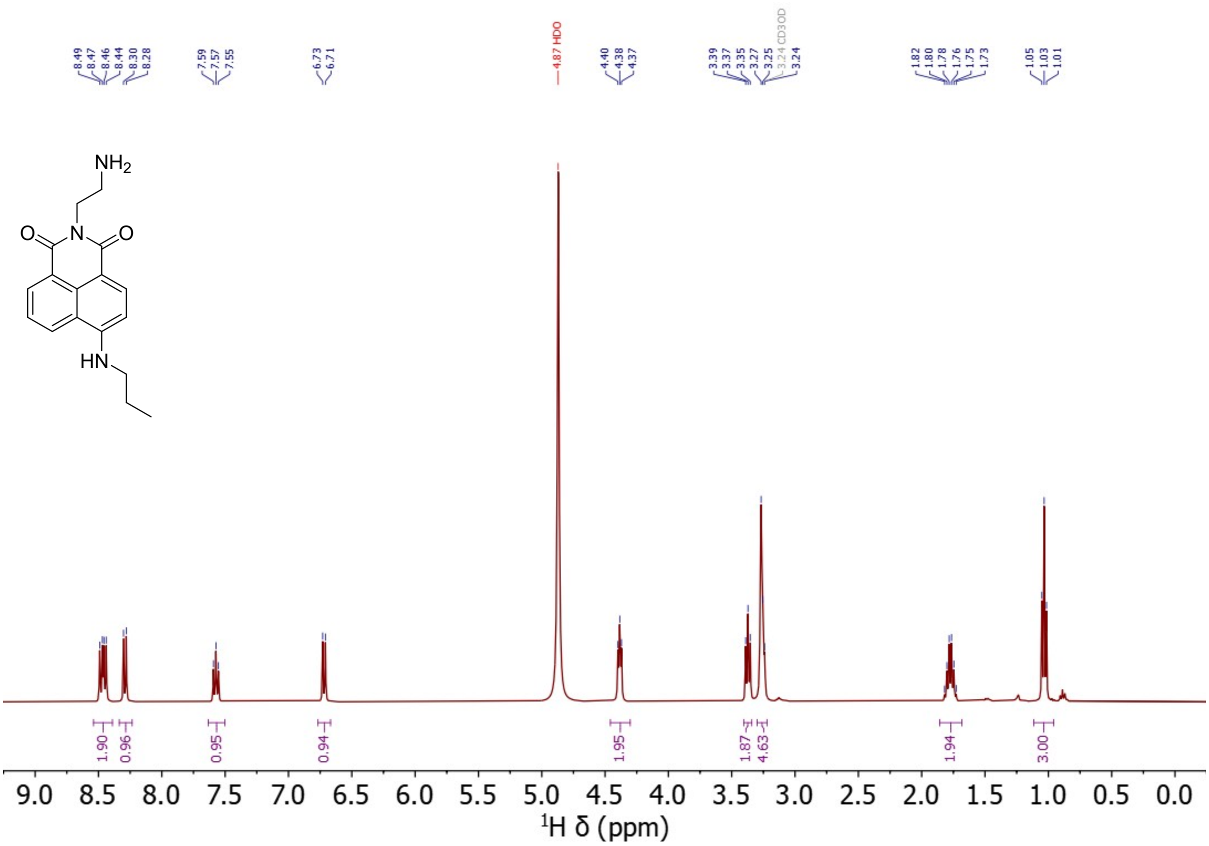

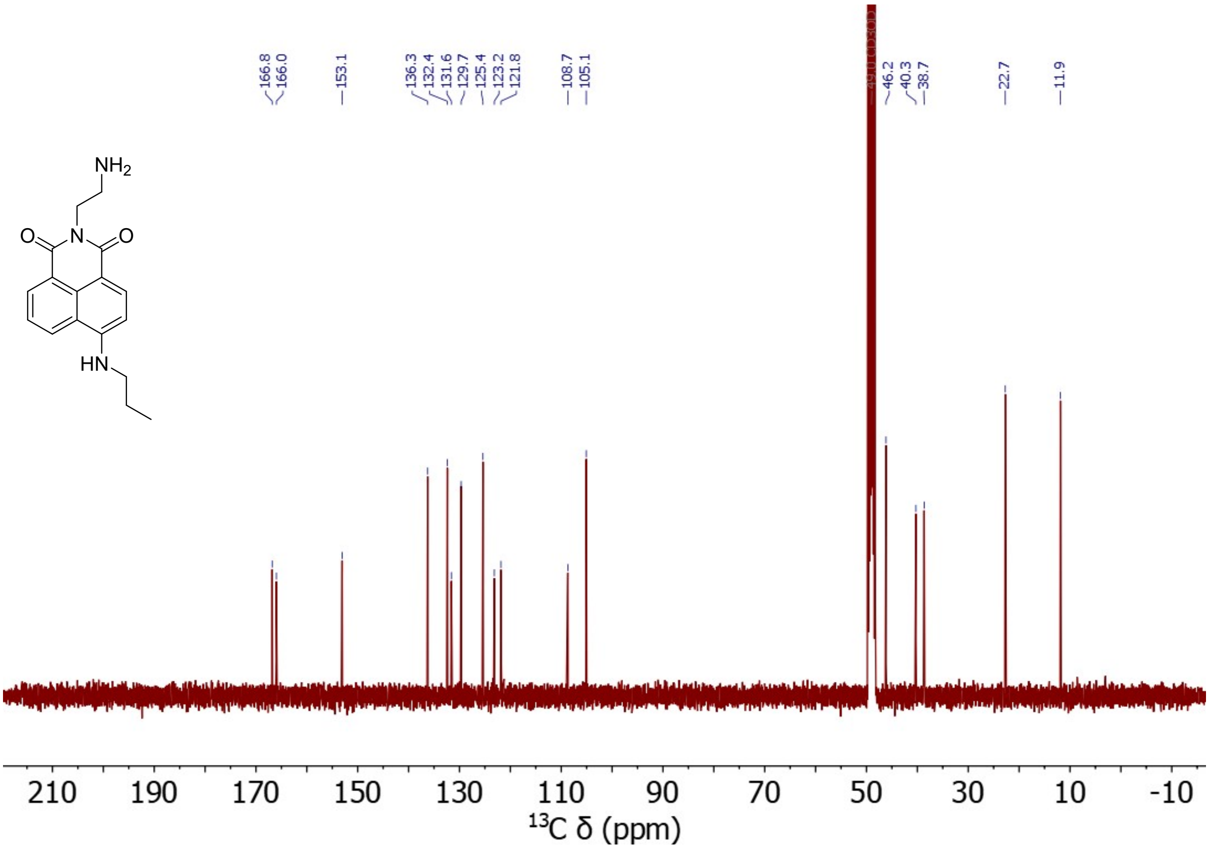

Figure S14. 400 MHz ^{1}H NMR spectrum of compound **9** in CD_3OD at 294 K.

Figure S15. 101 MHz ^{1}H NMR spectrum of compound **9** in CD_3OD at 294 K.

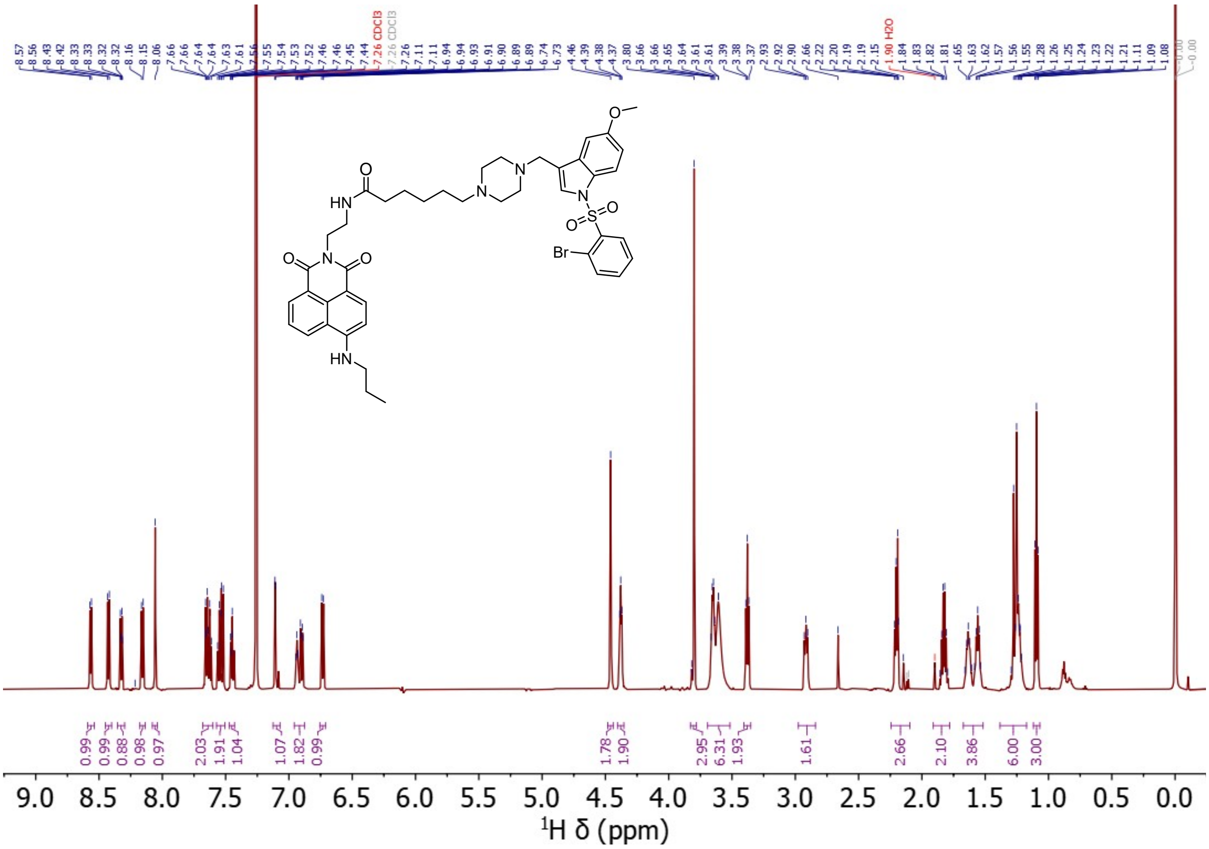

Figure S16. 600 MHz ^{1}H NMR spectrum of compound **5** in $CDCl_3$ at 296 K.

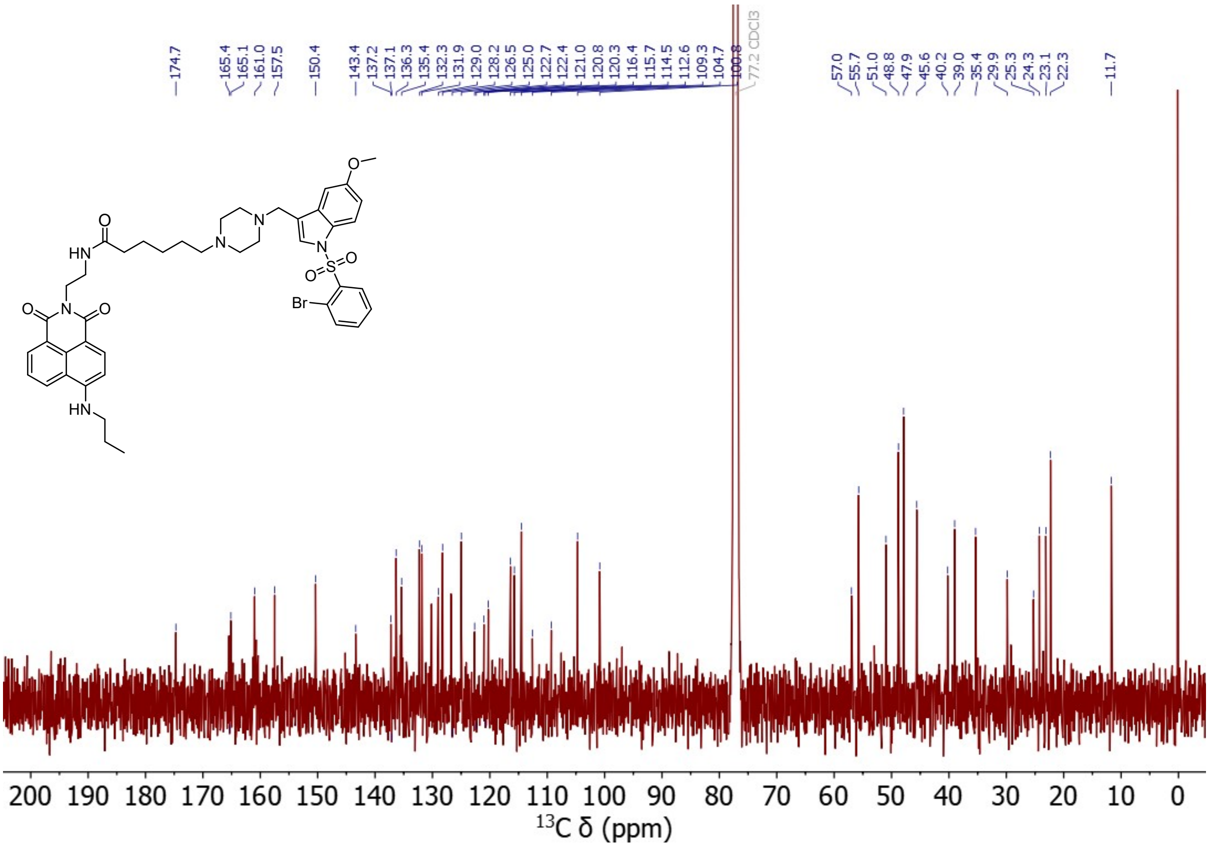

Figure S17. 151 MHz ^{13}C NMR spectrum of compound **5** in $CDCl_3$ at 296 K.

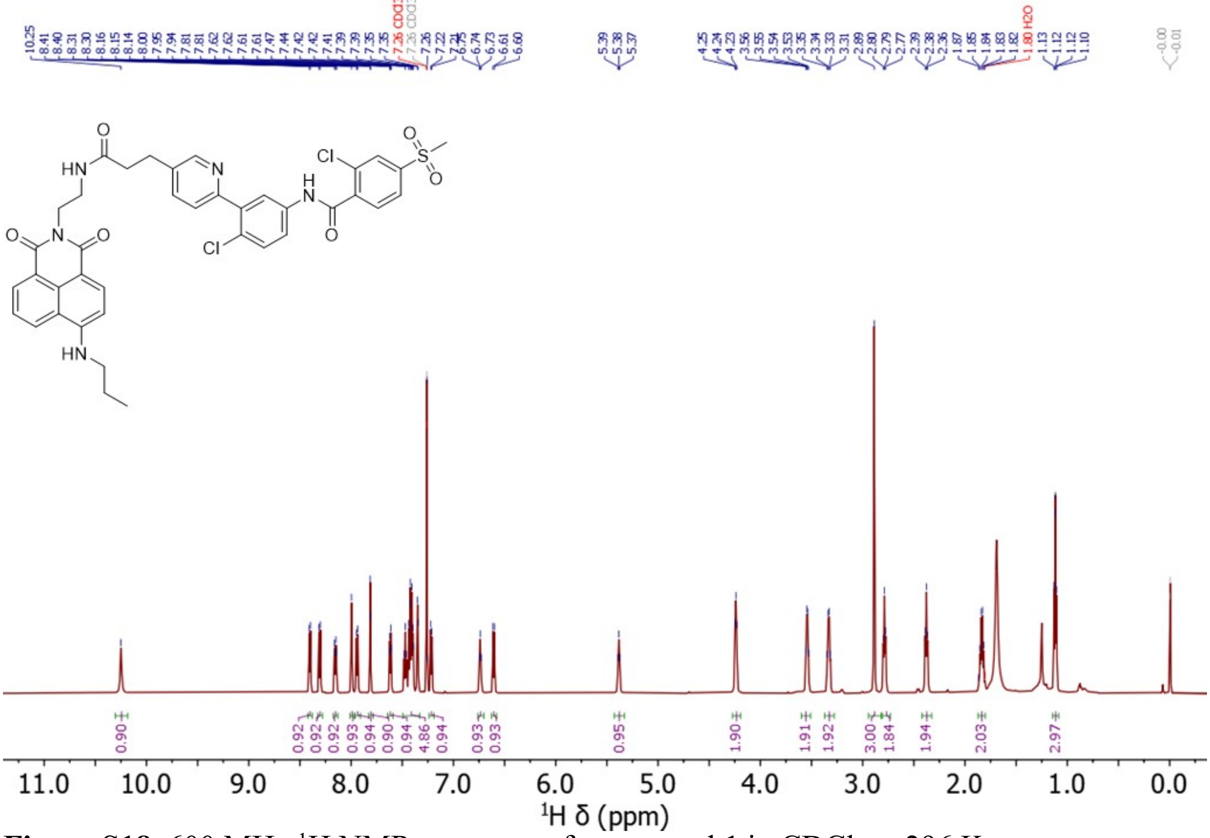

Figure S18. 600 MHz ^{1}H NMR spectrum of compound **1** in $CDCl_3$ at 296 K.

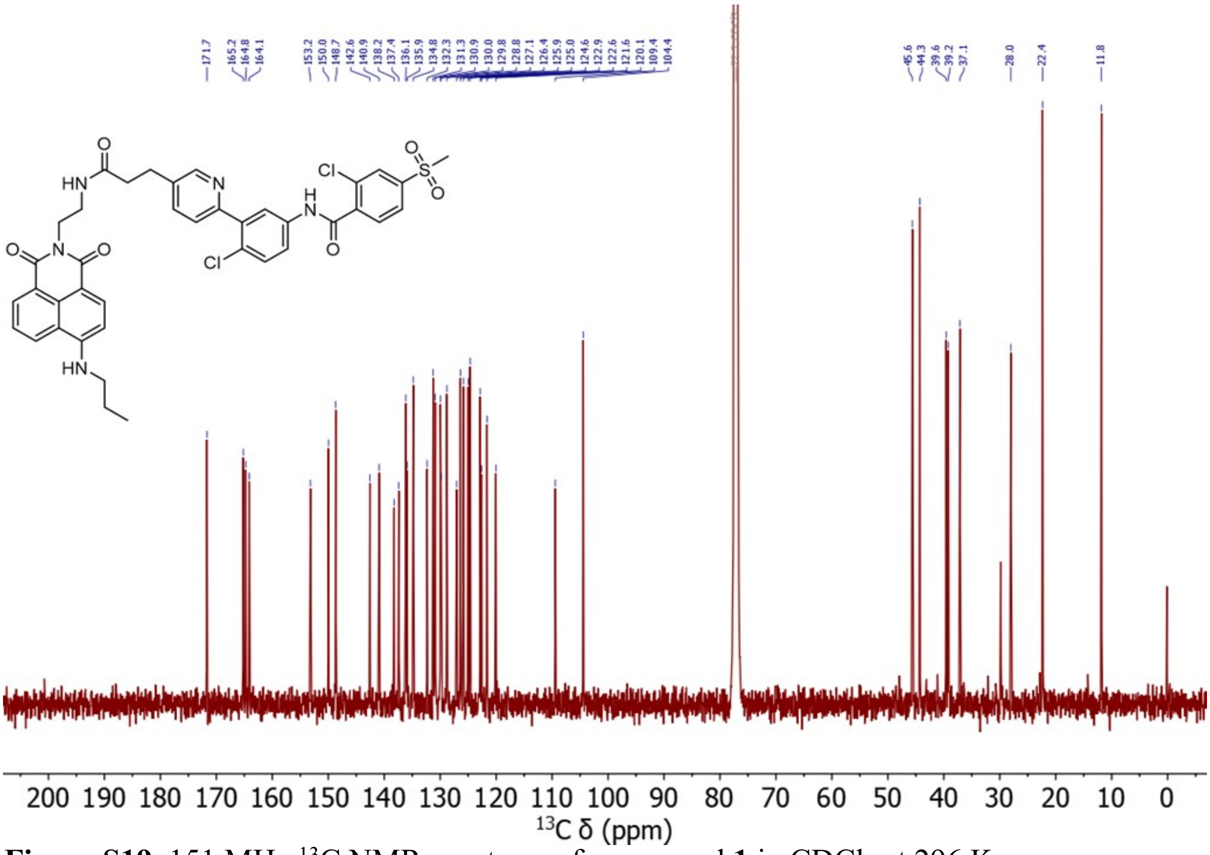

Figure S19. 151 MHz ^{13}C NMR spectrum of compound **1** in $CDCl_3$ at 296 K.

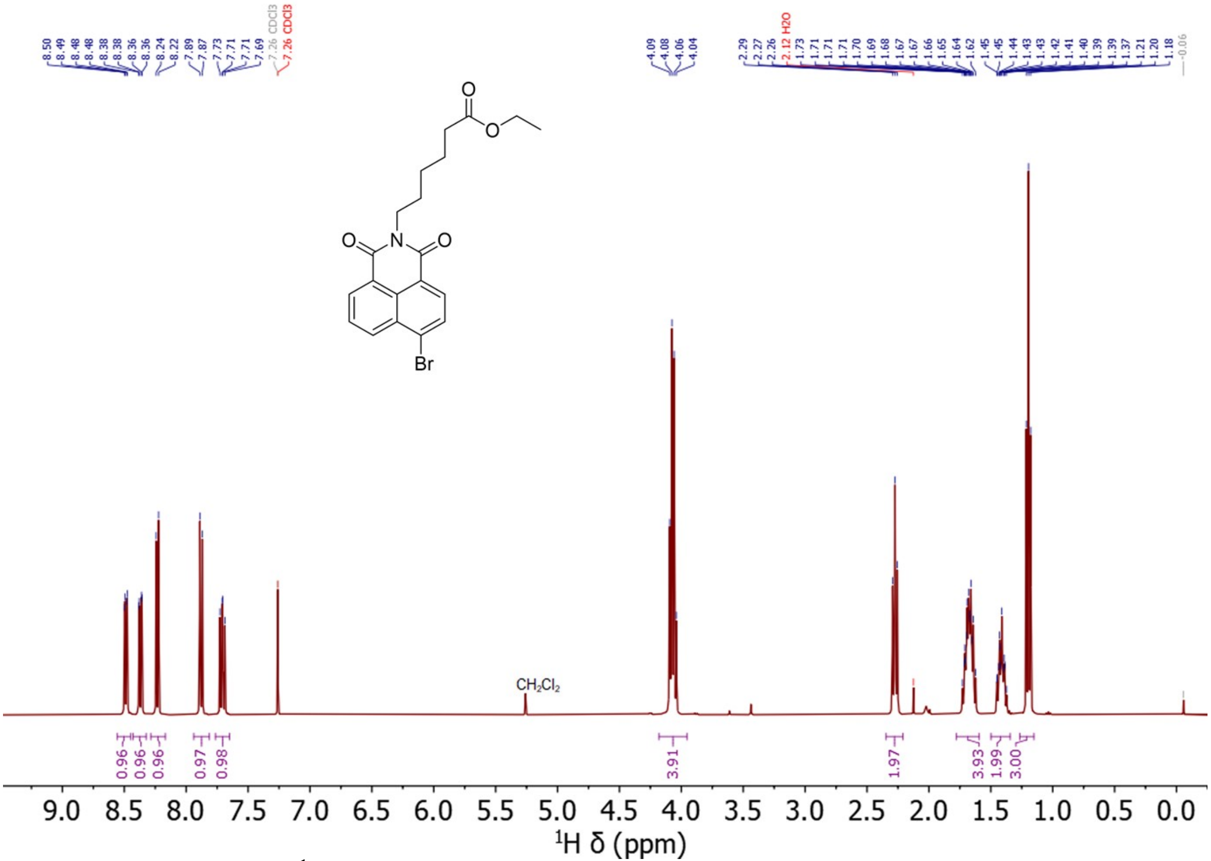

Figure S20. 400 MHz ^{1}H NMR spectrum of compound **16** in $CDCl_3$ at 294 K.

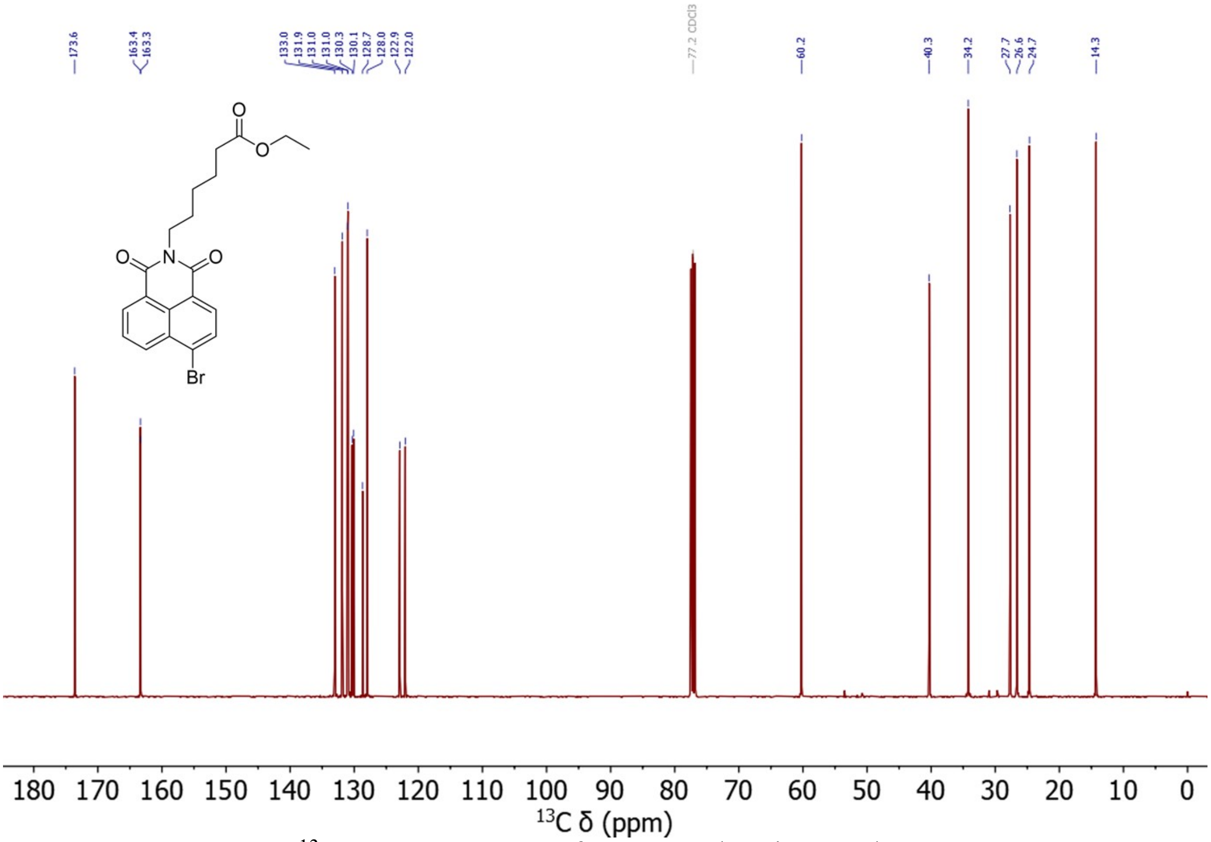

Figure S21. 101 MHz ^{13}C NMR spectrum of compound **16** in $CDCl_3$ at 294 K

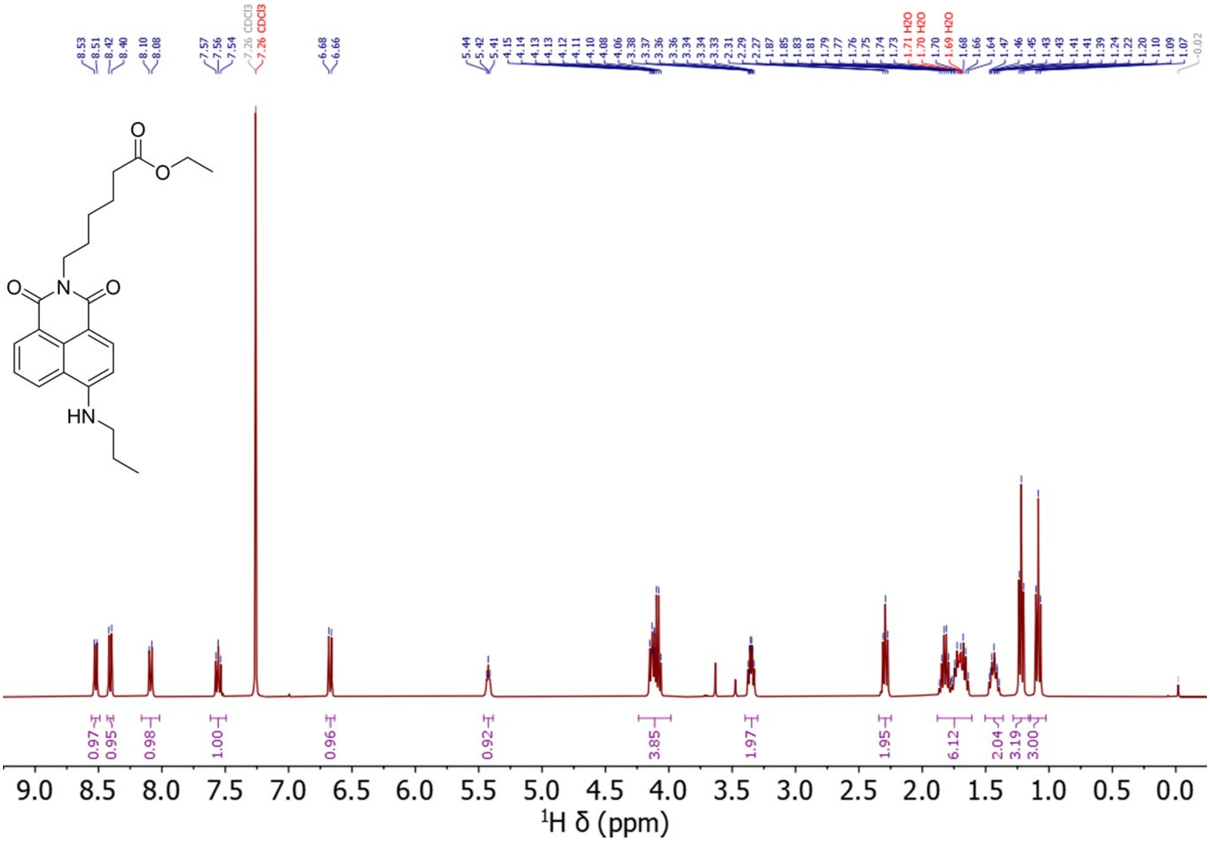

Figure S22. 400 MHz ^{1}H NMR spectrum of compound **17** in $CDCl_3$ at 294 K.

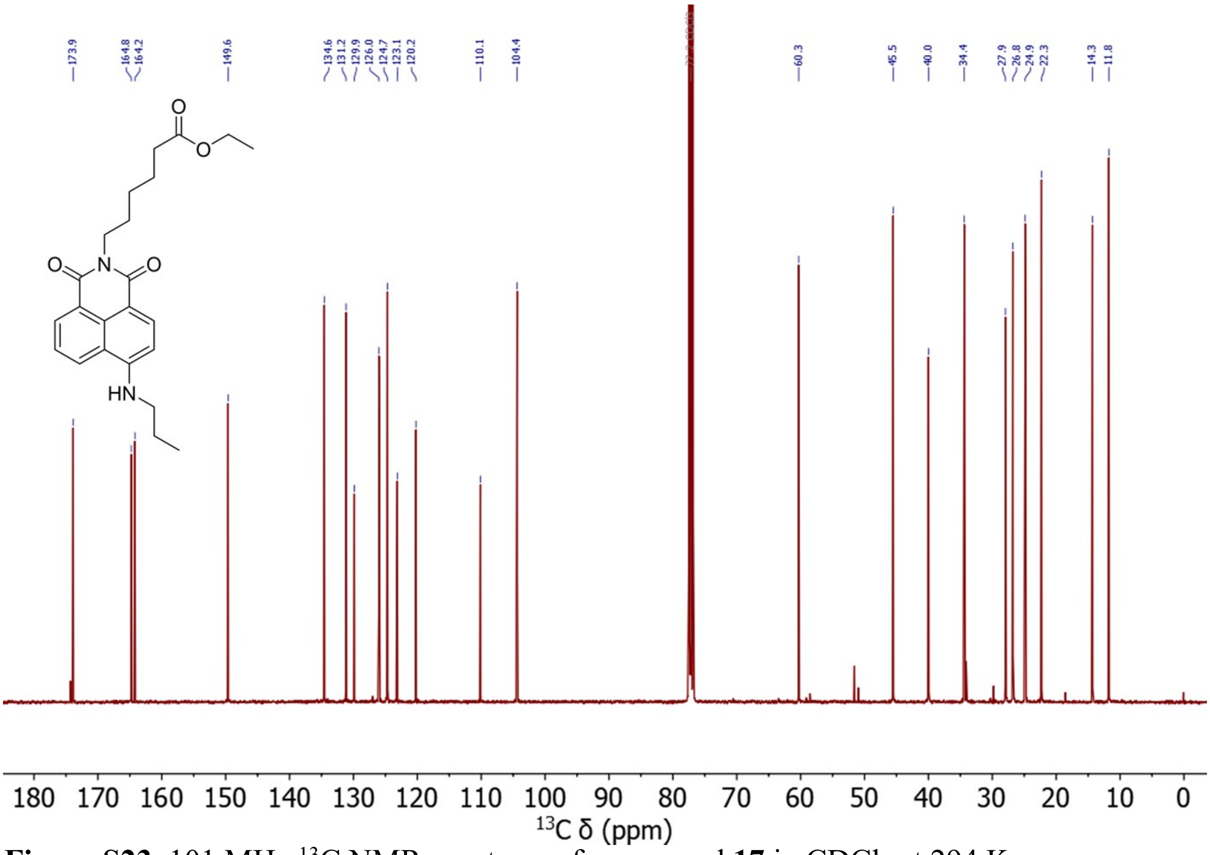

Figure S23. 101 MHz ^{13}C NMR spectrum of compound **17** in $CDCl_3$ at 294 K.

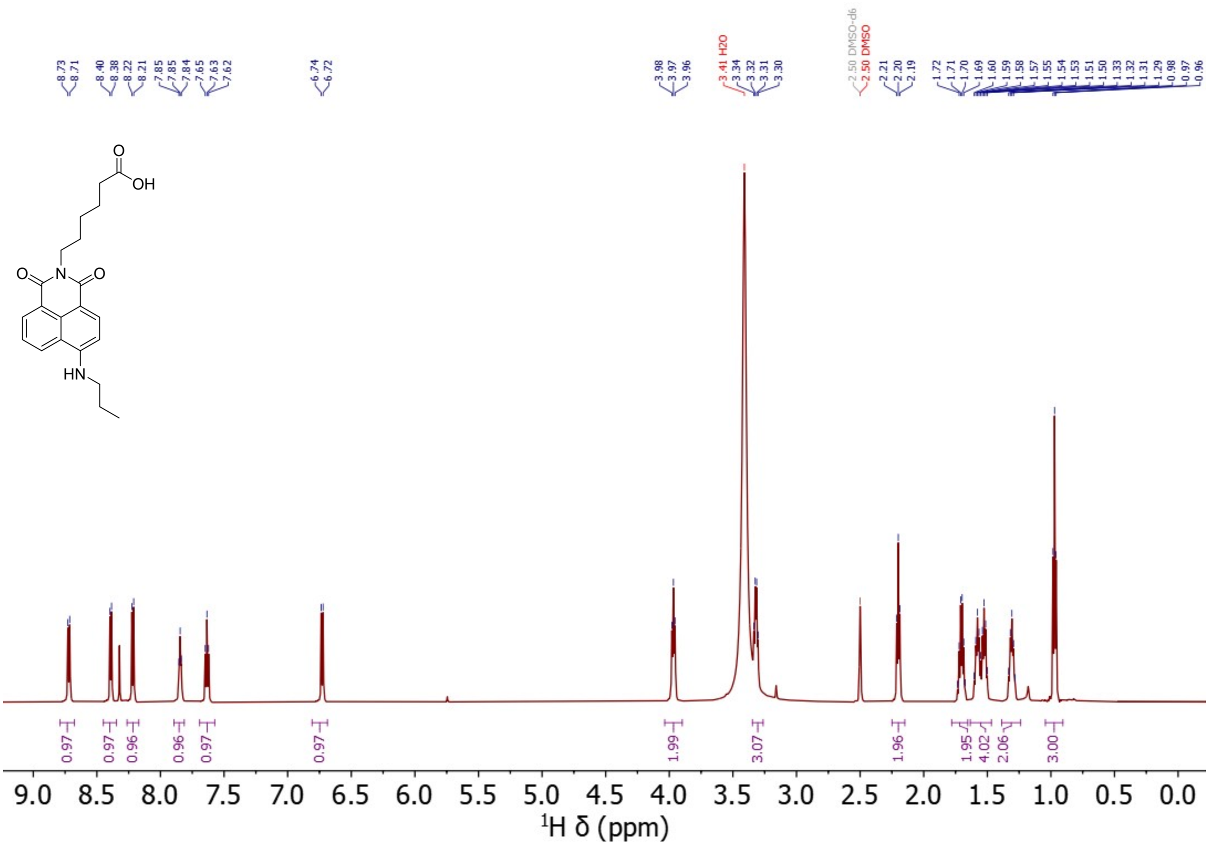

Figure S24. 600 MHz ^{1}H NMR spectrum of compound **18** in DMSO-d_6 at 296 K.

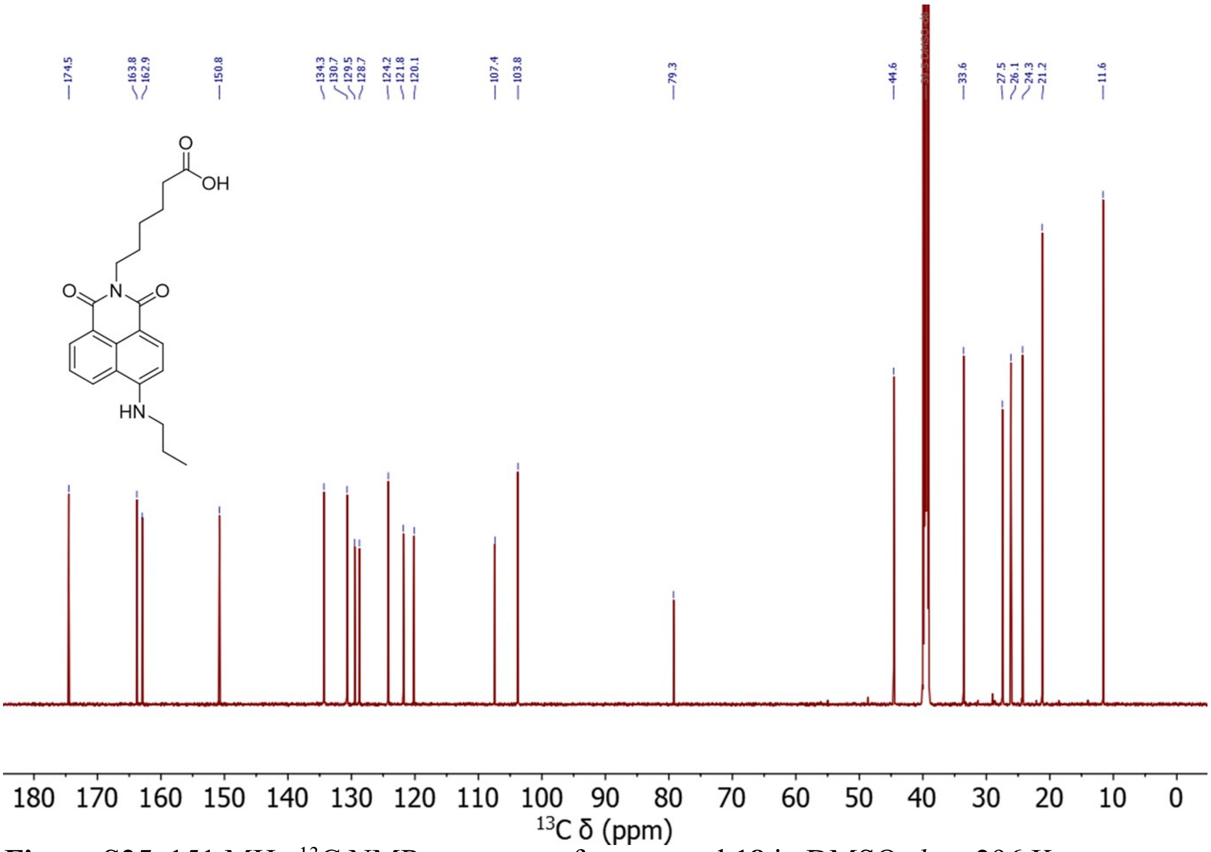

Figure S25. 151 MHz ^{13}C NMR spectrum of compound **18** in DMSO-d_6 at 296 K.

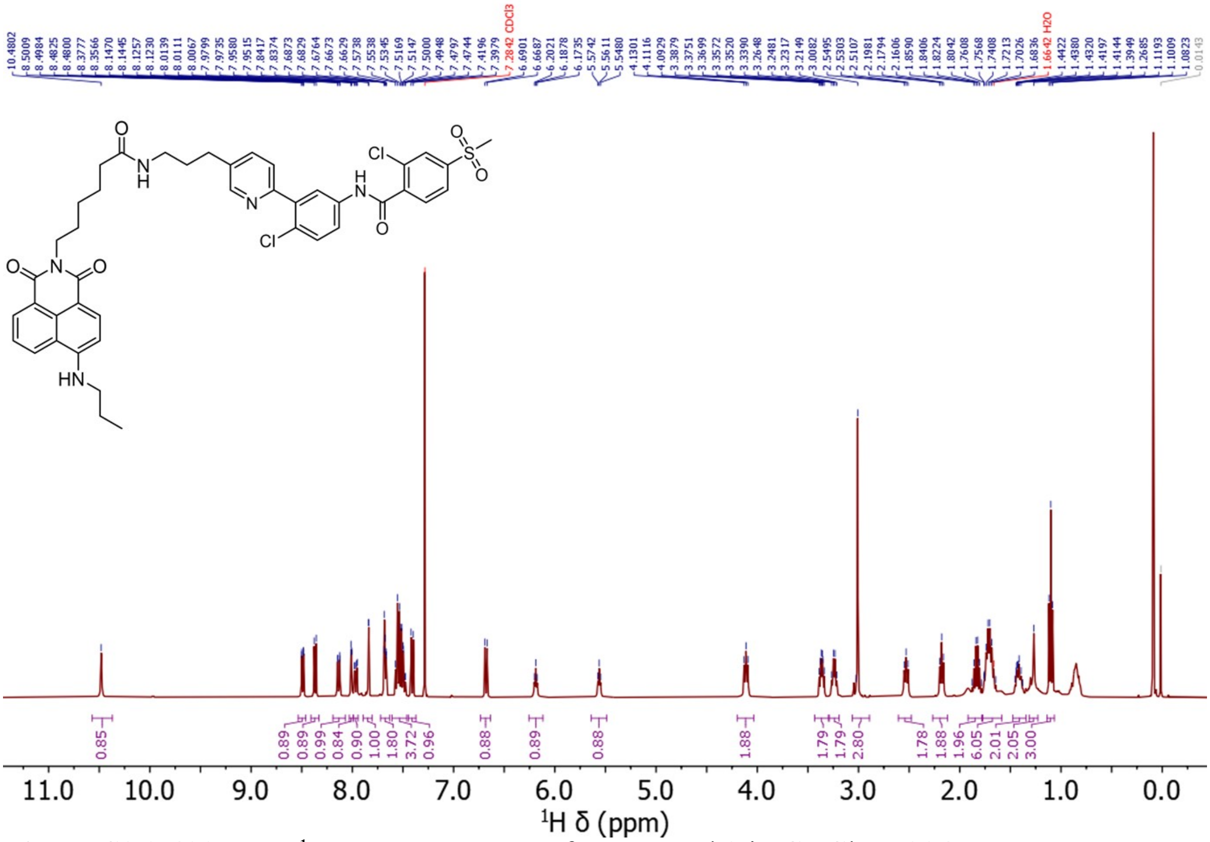

Figure S26. 400 MHz ^{1}H NMR spectrum of compound **2** in $CDCl_3$ at 294 K.

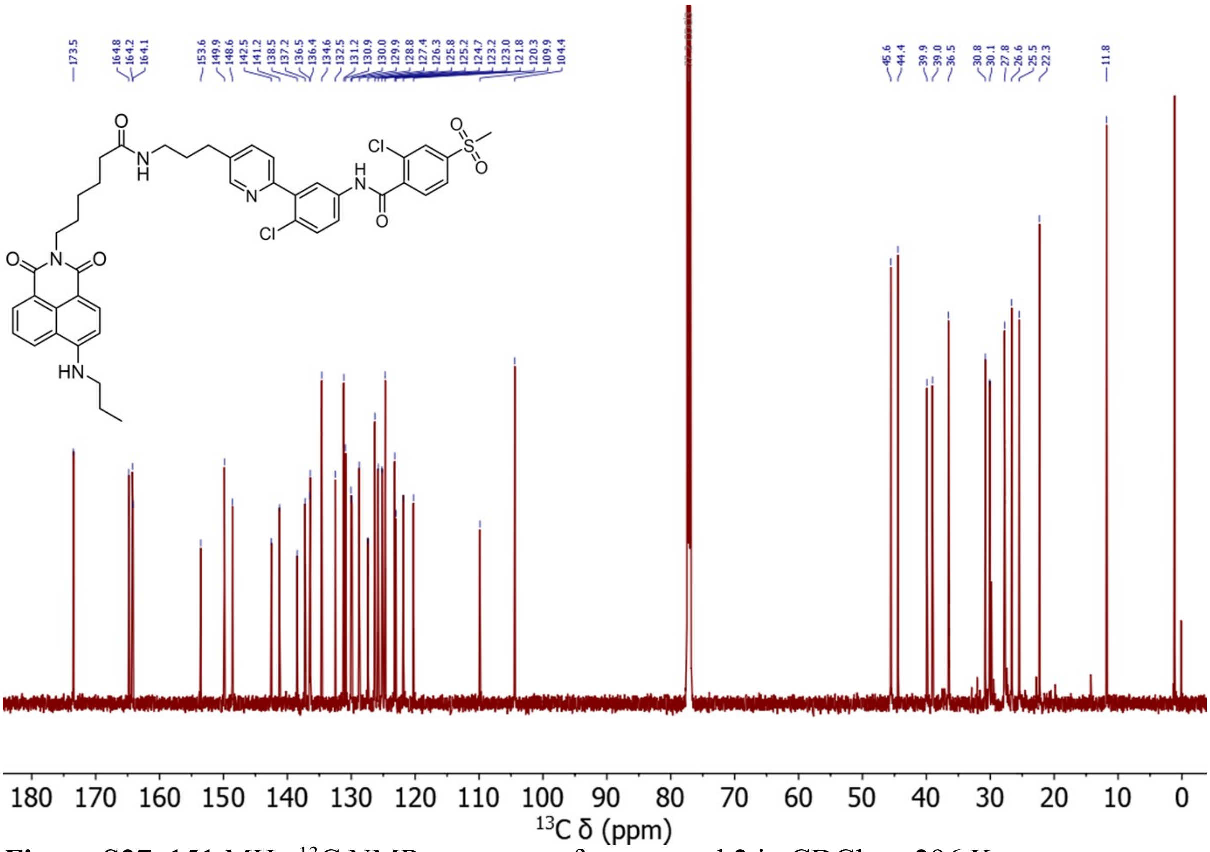

Figure S27. 151 MHz ^{13}C NMR spectrum of compound **2** in $CDCl_3$ at 296 K.

8. Photophysical Studies

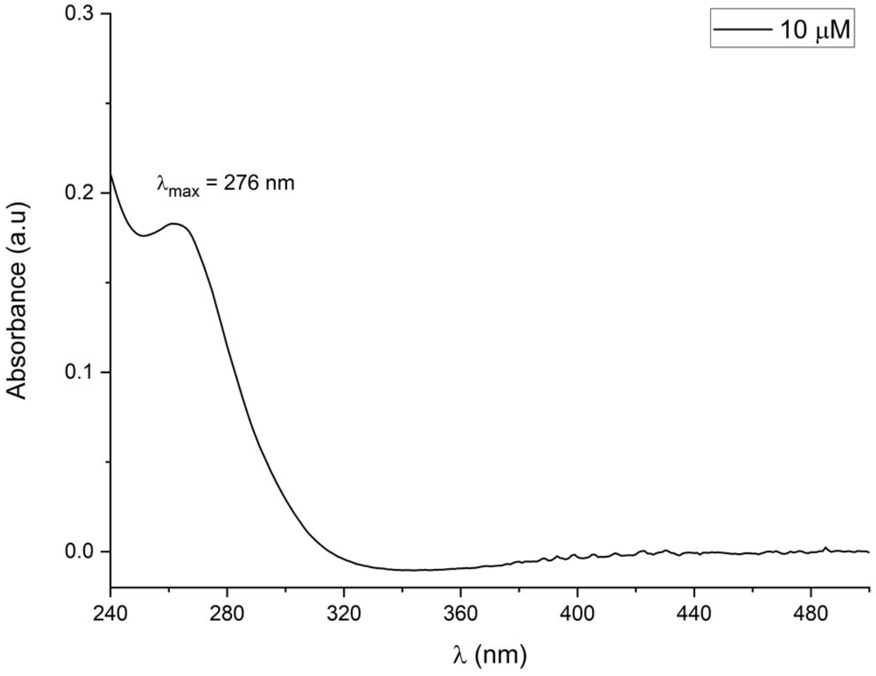

Figure S28. Electronic absorption spectrum of **Vismodegib** (0.01 M HEPES, pH 7.4).

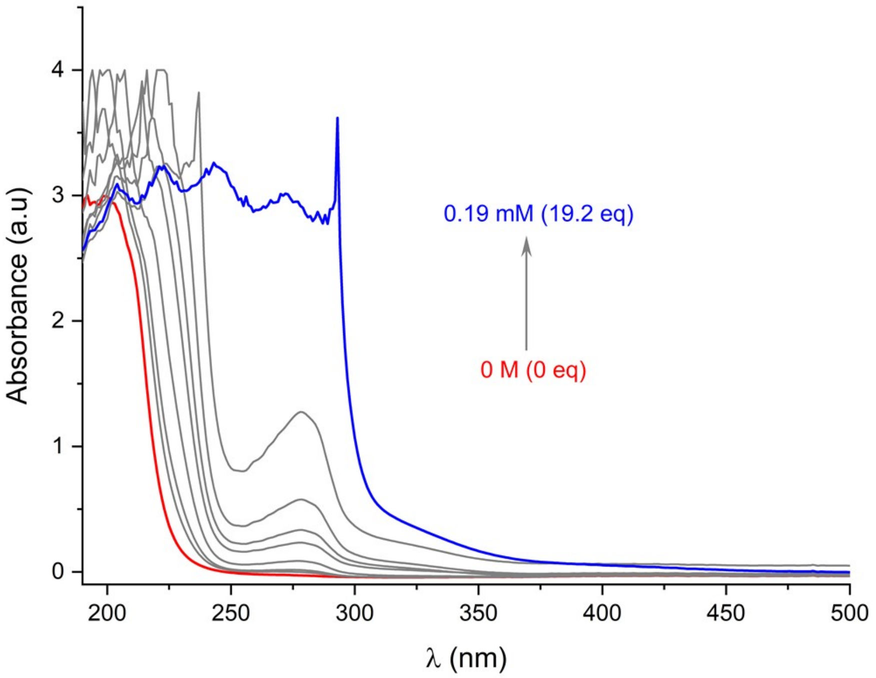

Figure S29. Electronic absorption titration spectra of **Vismodegib** (10 μM) following addition of BSA in (0.01 M HEPES, pH 7.4). Spectrum of Vismodegib in *red*, spectra upon the addition of BSA in *grey*, spectrum after the final addition of BSA in *blue*.

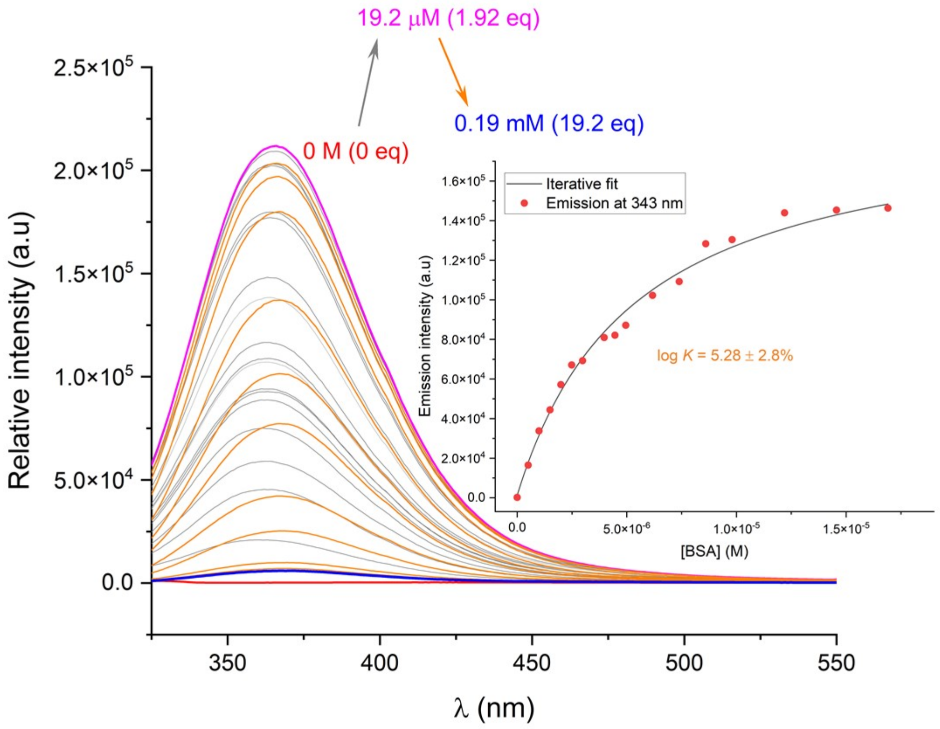

Figure S30. Steady-state fluorescence titration spectra of **Vismodegib** (10 μM, λ_{ex} = 276 nm) against BSA in 0.01 M HEPES at pH 7.4; spectrum of Vismodegib in *red*, spectra upon the incremental addition of BSA in *grey*, spectrum after the addition of 1.92 eq. BSA in *pink*, spectra upon the incremental addition of BSA in *orange*, spectrum after the final addition of BSA in *blue*. The inset shows the binding isotherm, obtained by plotting the emission intensity at 343 nm. The error on the apparent binding constant is expressed as the coefficient of variation as a percentage. *Note*: When generating the binding isotherm, the emission intensity points were truncated at 1.92 eq. of BSA added to improve the quality of the fit as the emission trend afterwards did not converge to any simple binding stoichiometry.

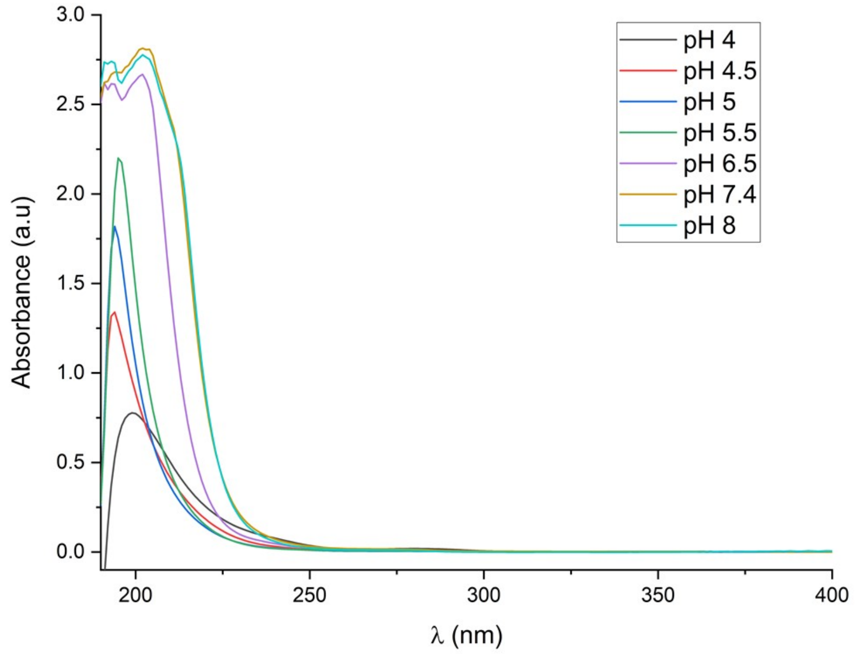

Figure S31. Electronic absorption spectra of compound **1** (1 μM) at different pH values.

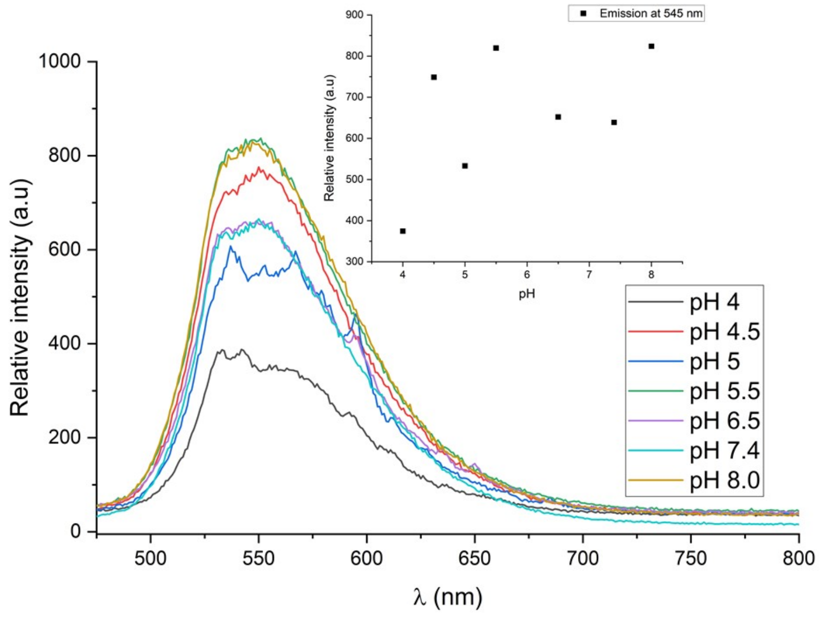

Figure S32. Steady-state fluorescence spectra of compound **1** (1 μM, λ_{ex} 458 nm) at different pH values. The inset shows the random variation of emission intensity at 545 nm with pH.

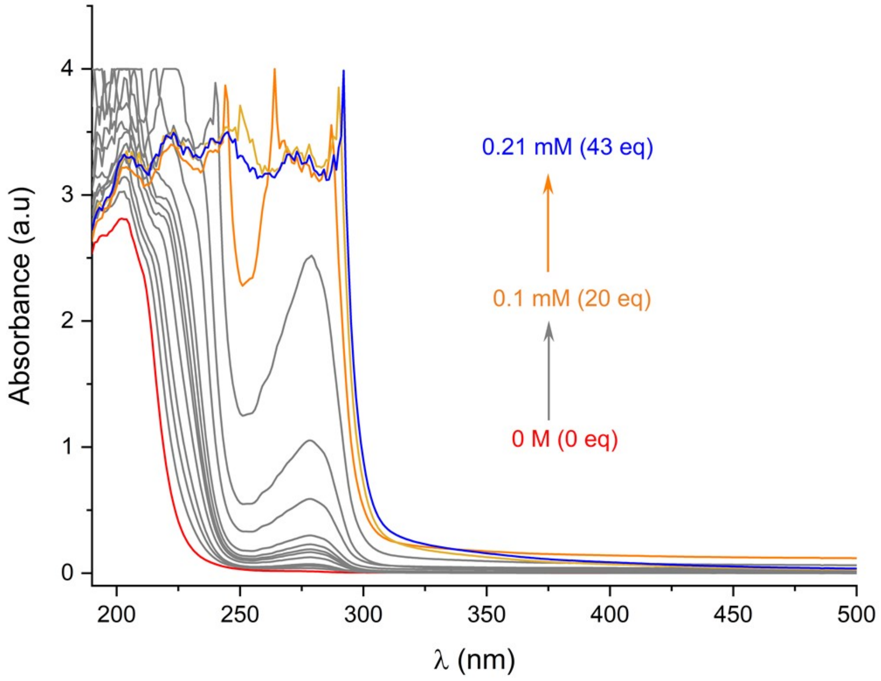

Figure S33. Electronic absorption titration spectra for compound **1** (1 μM) as a function of [BSA] (0.01 M HEPES, pH 7.4); spectrum of compound **1** in *red*, spectra following addition of BSA in *grey*, spectrum after 0.1 mM addition in *orange*, spectrum after the final addition of BSA in *blue*.

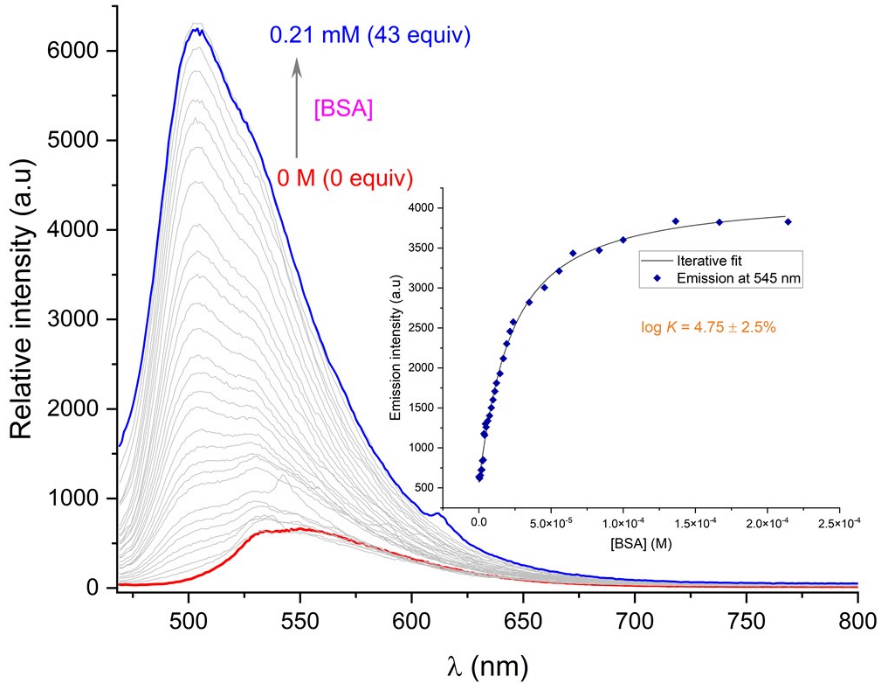

Figure S34. Steady-state fluorescence titration spectra of compound **1** (1 μM, λ_{ex} 458 nm) following addition of BSA (0.01 M HEPES, pH 7.4); spectrum of compound **1** in *red*, spectra following incremental addition of BSA in *grey*, spectrum after final addition of BSA in *blue*. The inset shows the binding isotherm, obtained plotting the emission intensity at 545 nm. Error on the apparent binding constant is expressed as the coefficient of variation as a percentage.

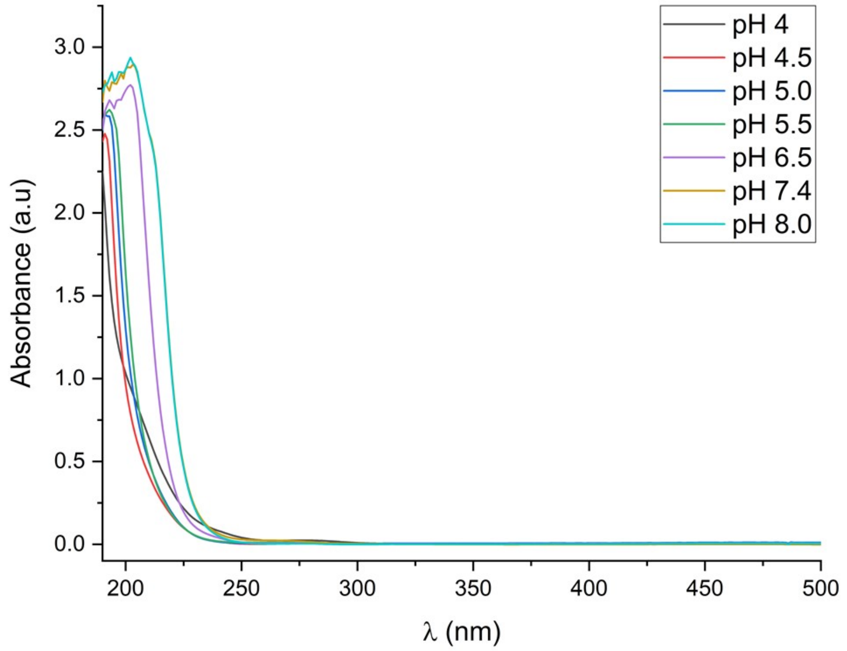

Figure S35. Electronic absorption spectra of compound **2** (1 μM) at different pH values.

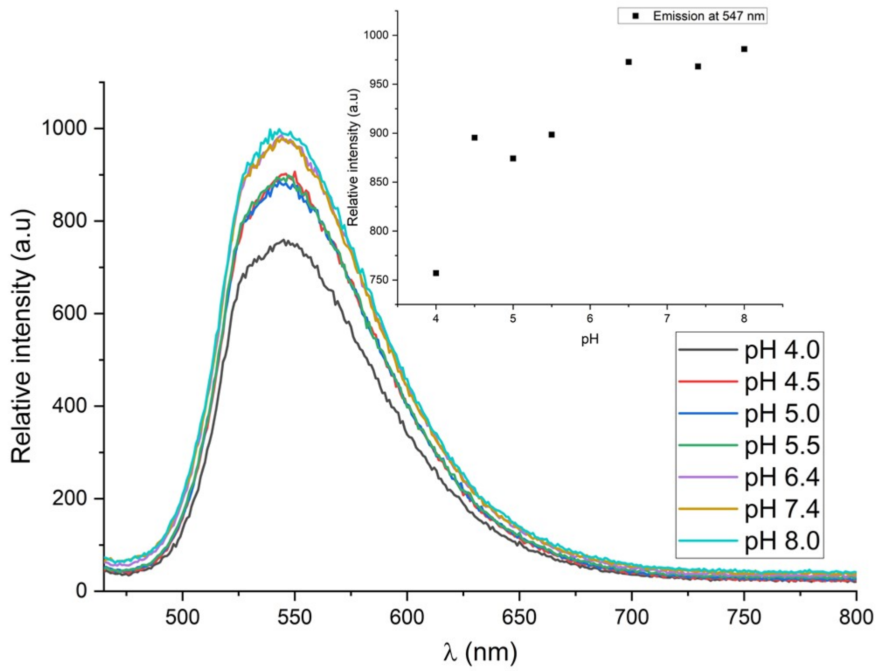

Figure S36. Steady-state fluorescence spectra of compound **2** (1 μM, λ_{ex} 458 nm) at different pH values. The inset shows the random variation of emission intensity at 547 nm with pH.

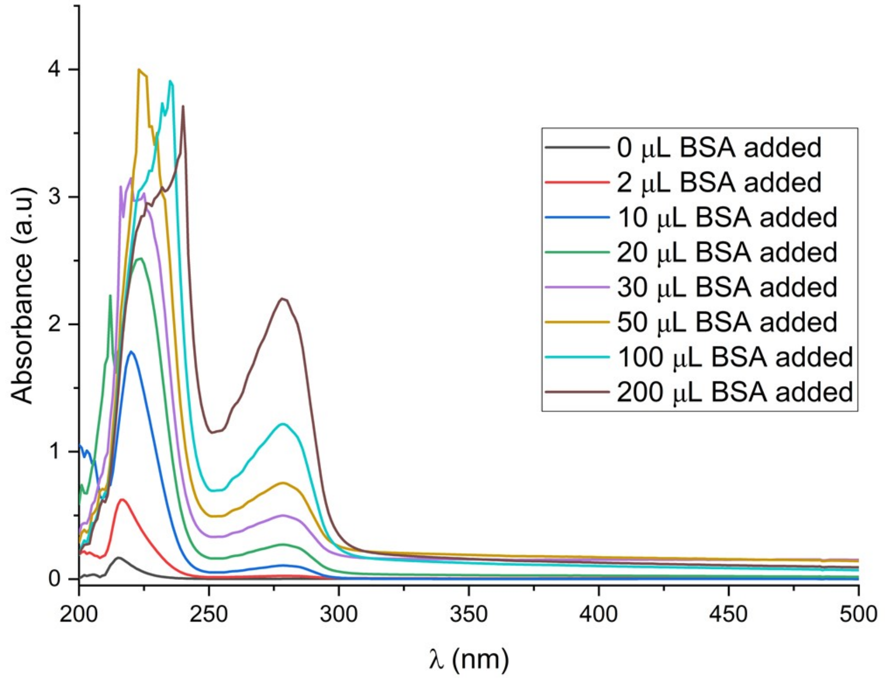

Figure S37. Electronic absorption titration spectra for compound **2** (1 μM) as a function of [BSA] (0.01 M HEPES, pH 7.4). Stock concentration of BSA = 0.5 mM.

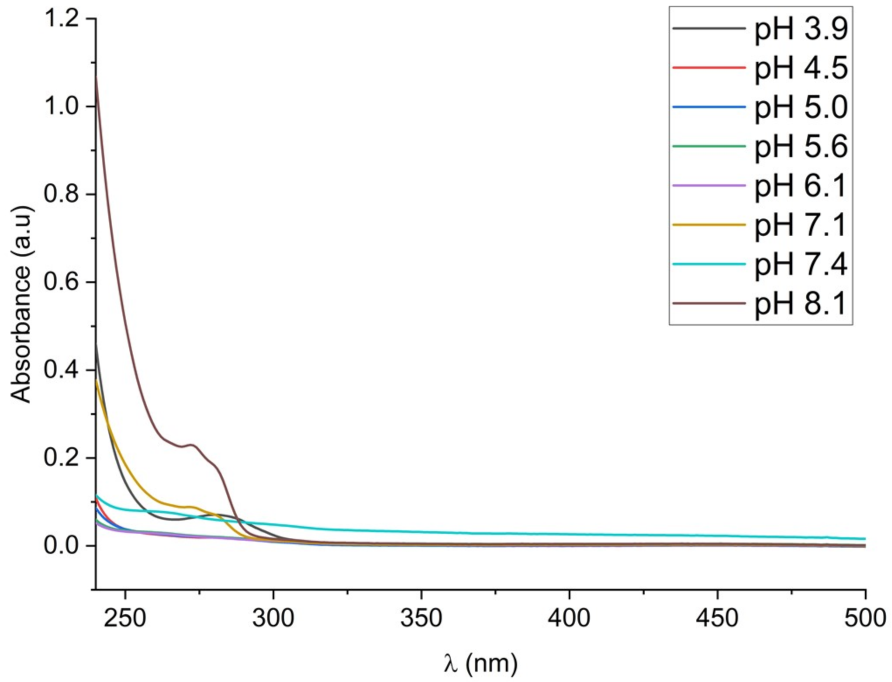

Figure S38. Electronic absorption spectra of compound **5** (1 μM) at different pH values.

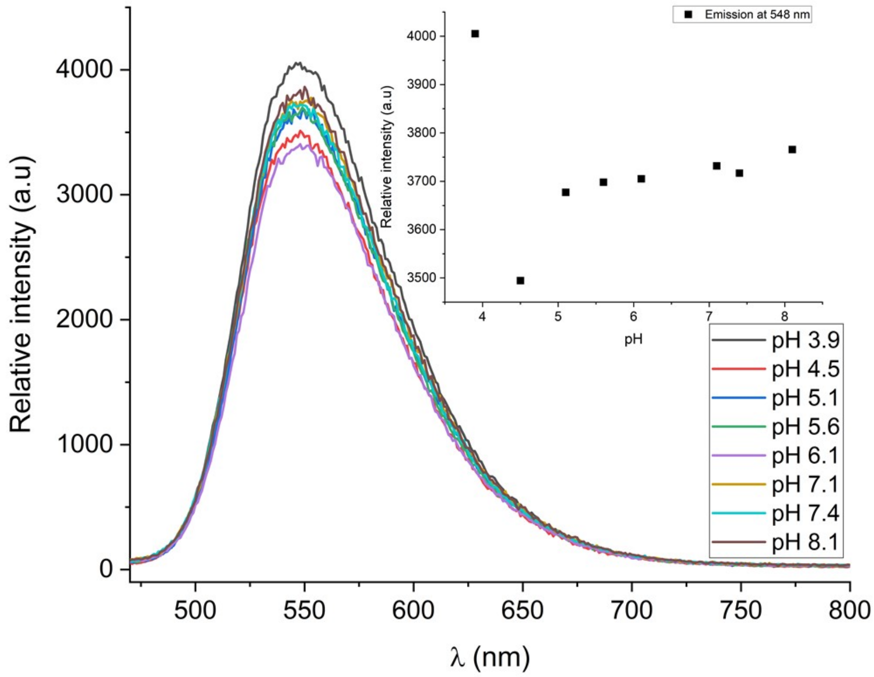

Figure S39. Steady-state fluorescence spectra of compound **5** (1 μM, λ_{ex} 450 nm) at different pH values. The inset shows the random variation of emission intensity at 548 nm with pH.

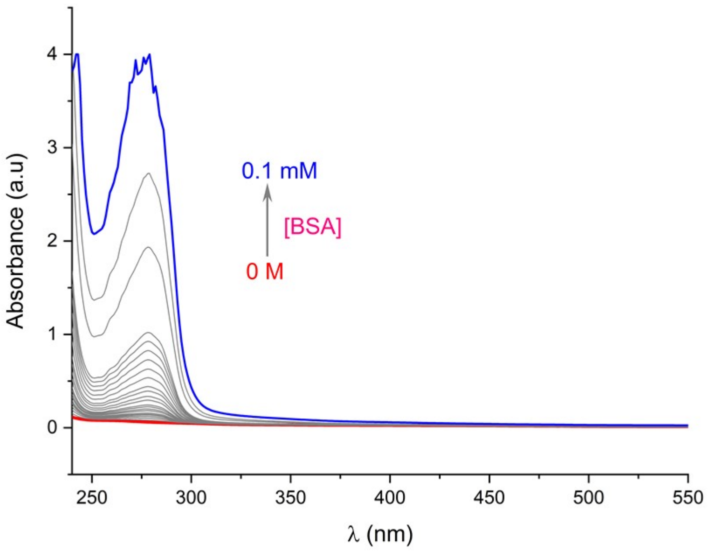

Figure S40. Electronic absorption titration spectra for compound **5** (1 μM) as a function of [BSA] (0.01 M HEPES, pH 7.4); Spectrum of compound **5** in *red*, spectra upon the addition of BSA in *grey*, spectrum after the final addition of BSA in *blue*.

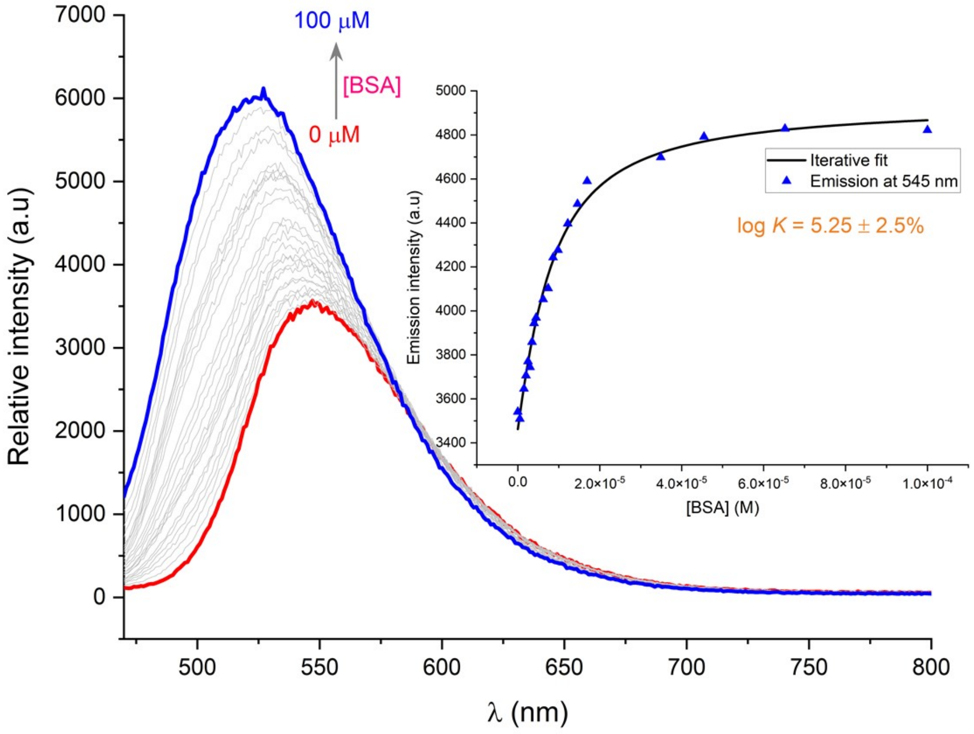

Figure S41. Steady-state fluorescence titration spectra of compound **5** (1 μM, λ_{ex} = 458 nm) following addition of BSA (0.01 M HEPES, pH 7.4); spectrum of compound **5** in *red*, spectra following incremental addition of BSA in *grey*, spectrum after final addition of BSA in *blue*. The inset shows the binding isotherm, obtained plotting the emission intensity at 545 nm. Error on the apparent binding constant is expressed as the coefficient of variation as a percentage.

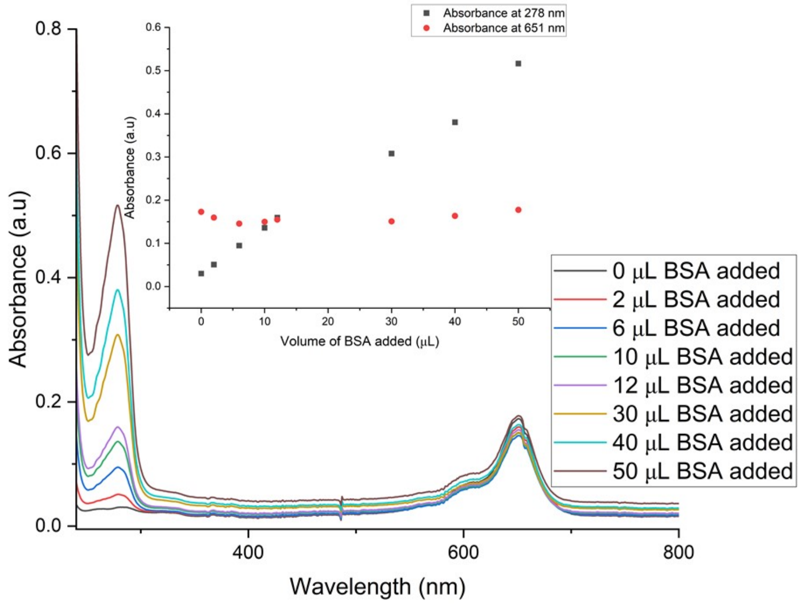

Figure S42. Electronic absorption titration spectra for compound **6** (1 μM) as a function of [BSA] (0.01 M HEPES, pH 7.4); Stock concentration of BSA is 0.5 mM. Inset shows the variation of absorbance at 278 nm (in black square) and 651 nm (red circle).

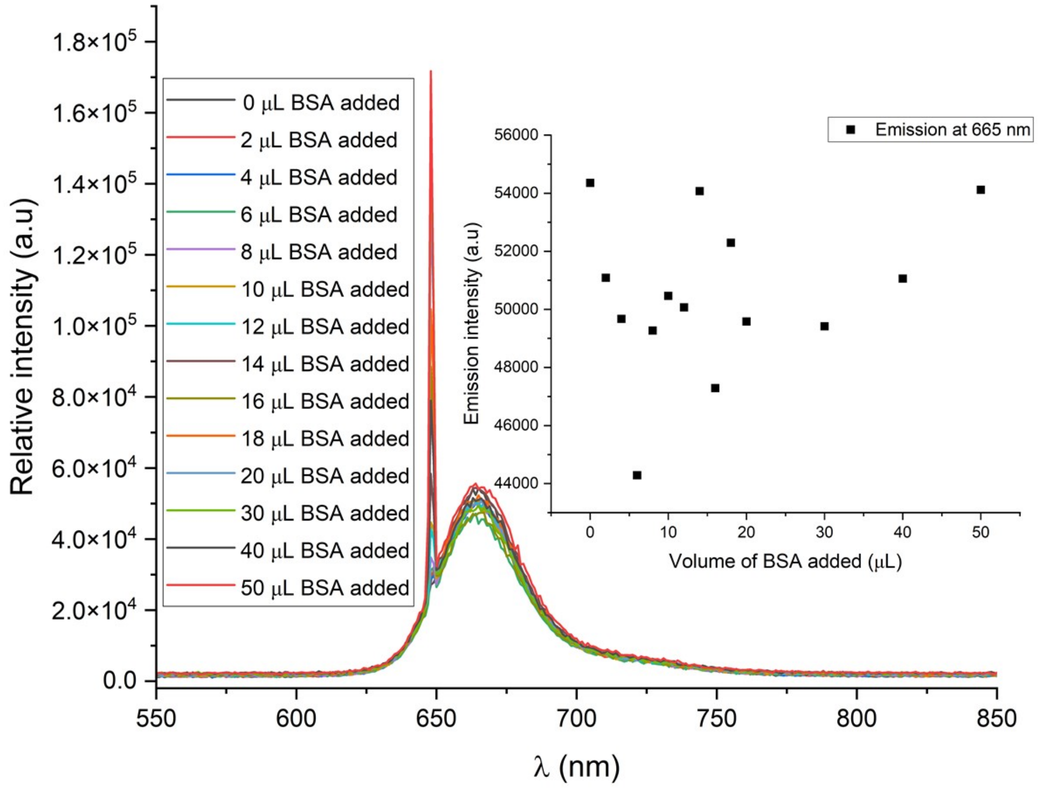

Figure S43. Steady-state fluorescence titration spectra of compound **6** (1 μM, λ_{ex} = 458 nm) following addition of BSA (0.01 M HEPES, pH 7.4); Stock concentration of BSA is 0.5 mM. Inset shows the random variation in emission intensity at 665 nm with [BSA].

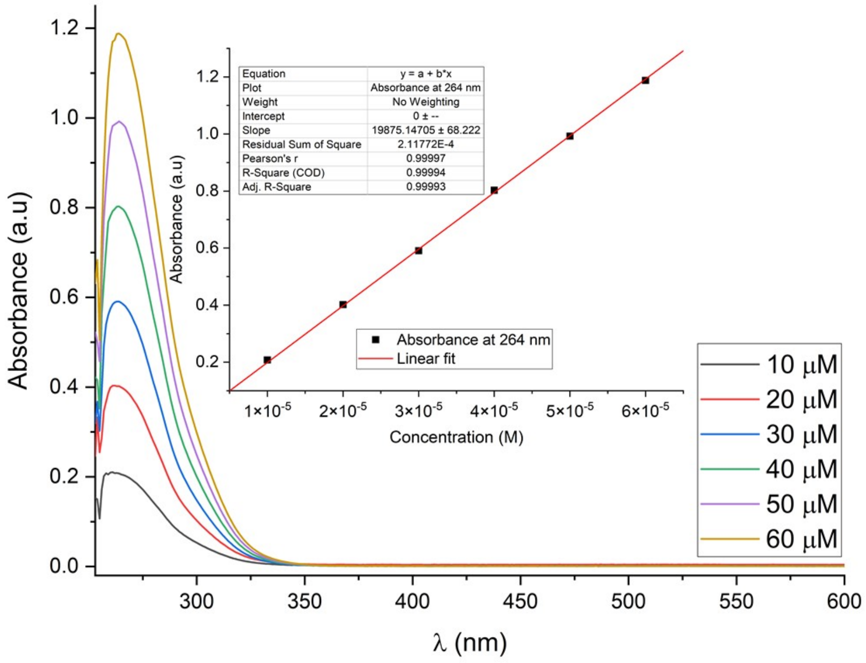

Figure S44. Molar extinction coefficient determination of **Vismodegib** in DMSO. Inset shows the linear fit of absorbance vs concentration, monitored at 264 nm.

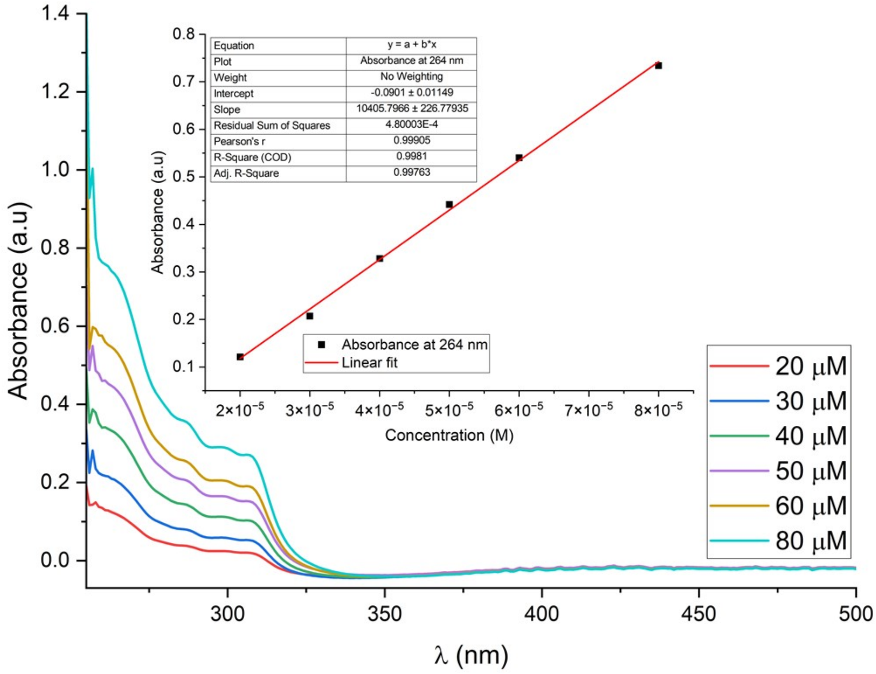

Figure S45. Molar extinction coefficient determination of **Masupirdine** in DMSO. Inset shows the linear fit of absorbance vs concentration, monitored at 264 nm.

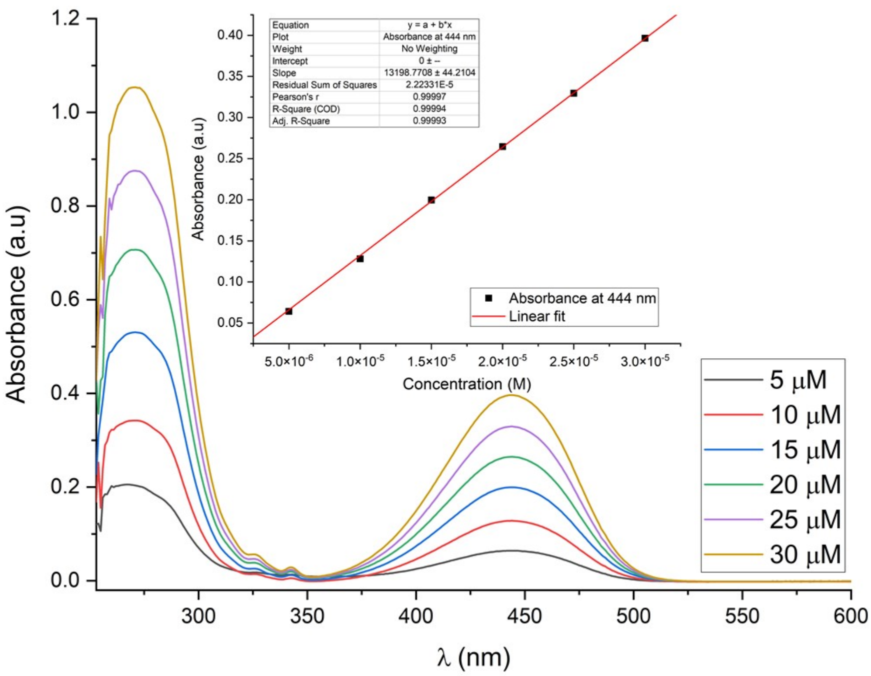

Figure S46. Molar extinction coefficient determination of compound **1** in DMSO. Inset shows the linear fit of absorbance vs concentration monitored at 444 nm.

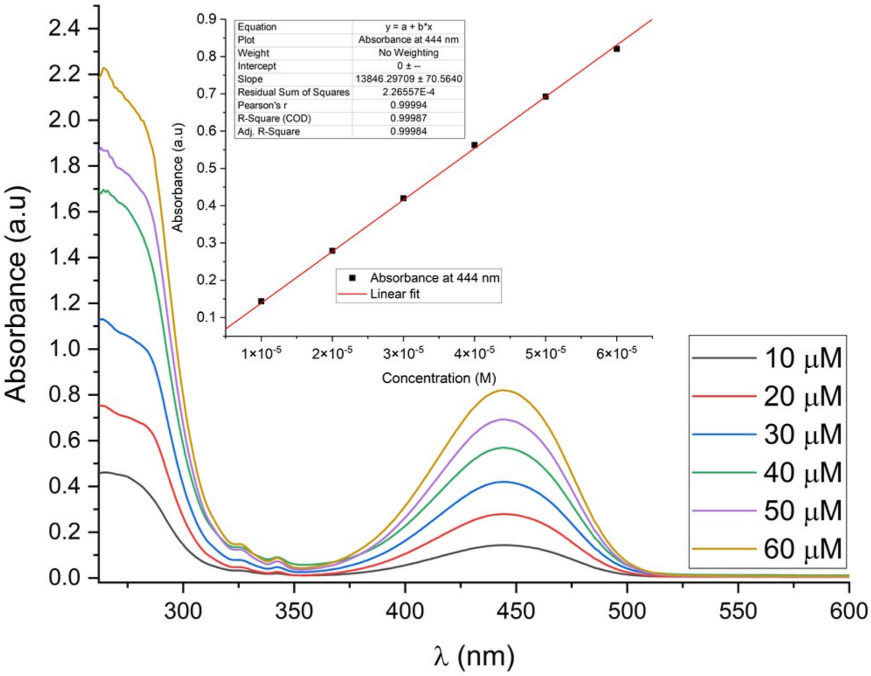

Figure S47. Molar extinction coefficient determination for compound **2** in DMSO. Inset shows the linear fit of absorbance vs concentration monitored at 444 nm.

9. X-ray crystal structure data

Table S3. Crystal data and structure refinement for **Masupirdine** and compound **12**. Crystal structures are shown in Figure 3 of the article.

**Parameters**	**Masupirdine**	Compound **12**
CCDC number	2529009	2529010
Empirical formula	$C_{21}H_{24}N_3O_3SBr$	$C_{25}H_{30}N_3O_5SBr$
Formula weight	478.40	564.49
Temperature	100 K	100 K
Wavelength	0.71073 Å	0.71073 Å
Crystal system	monoclinic	monoclinic
Space group	$P2_1/c$	$C\ 1\ 2/c1$
Unit cell dimensions	a = 10.5898(11) Å<br>b = 8.5491(9) Å<br>c = 23.402(3) Å<br>α = 90°<br>β = 93.780(4)°<br>γ = 90°	a = 30.1649(18) Å<br>b = 9.4466(6) Å<br>c = 18.2229(11) Å<br>α = 90°<br>β = 106.637(2)°<br>γ = 90°
Volume	2114.1(4) Å^3	4975.3(5) Å^3
Z	4	8
Density (calculated)	1.503 g cm^{-3}	1.507 g cm^{-3}
Absorption coefficient	2.071 mm^{-1}	1.778 mm^{-1}
F(000)	984.0	2336.0
Crystal size (mm^3)	0.24 × 0.15 × 0.02	0.4 × 0.2 × 0.1
Theta range for data collection	5.026° to 55.028°	4.536° to 55.042°
Index ranges	$-13 \leq h \leq 13$,<br>$-11 \leq k \leq 11$,<br>$-30 \leq l \leq 30$	$-39 \leq h \leq 39$,<br>$-12 \leq k \leq 12$,<br>$-23 \leq l \leq 23$
Reflections collected	97566	144845
Independent reflections	4850 [R(int) = 0.0465, R(sigma) = 0.0218]	5697 [R(int) = 0.0295, R(sigma) = 0.0171]
Completeness to theta = 73.257°	99.8%	99.4%
Theta (max)	27.514	27.521
Absorption correction	Multiscan	Multiscan
Max. and min. transmission	0.605 and 0.746	0.659 and 0.837
Refinement method	ShelXL (Least Squares minimisation)	ShelXL (Least Squares minimisation)
Data / restraints / parameters	4850/0/264	5697/0/320
Goodness-of-fit on F^2	1.071	1.064
Final R indices [I>2σ(I)]	R_1 = 0.0216,<br>wR_2 = 0.0539	R_1 = 0.0278,<br>wR_2 = 0.0735
R indices (all data)	R_1 = 0.0222,<br>wR_2 = 0.0544	R_1 = 0.0281,<br>wR_2 = 0.0737
Largest diff. peak and hole	0.35/-0.33 e.Å^{-3}	0.54/-0.93 e.Å^{-3}

10. References

1. J. Leonard, B. Lygo and G. Procter, Carrying out the reaction, In Advanced Practical Organic Chemistry, 3rd edition, CRC Press Taylor and Francis Group, USA, 2013, pp 137–189.

2. G. R. Fulmer, A. J. M. Miller, N. H. Sherden, H. E. Gottlieb, A. Nudelman, B. M. Stoltz, J. E. Bercaw and K. I. Goldberg, NMR Chemical shifts of trace impurities: Common laboratory solvents, organics, and gases in deuterated solvents relevant to the organometallic chemist, *Organometallics*, 2010, **29**, 2176 – 2179.

3. (a) P. Kuzmič, Program DYNAFIT for the analysis of enzyme kinetic data: application to HIV proteinase, *Anal. Biochem.*, 1996, **237**, 260–273; (b) P. Kuzmič, Chapter 10, DYNAFIT-A software package for enzymology, *Methods Enzymol.*, 2009, **467**, 247–280.

4. O. V. Dolomanov, L. J. Bourhis, R. J. Gildea, J. A. K. Howard and H. Puschmann, OLEX2: a complete structure solution, refinement and analysis program, *J. Appl. Cryst.*, 2009, **42**, 339 – 341.

5. G. M. Sheldrick, A short history of SHELX, *Acta Crystallogr. A*, 2008, **64**, 112 – 122.

6. G. M. Sheldrick, Crystal structure refinement with SHELXL, *Acta Crystallogr C Struct Chem.*, 2015, **71**, 3 – 8.

7. P. Kumar, A. Nagarajan, P. D. Uchil, Analysis of Cell Viability by the MTT Assay, *Cold Spring Harb. Protoc.,* 2018. DOI:10.1101/pdb.prot095505.

8. M. Ghasemi, T. Turnbull, S. Sebastian and I. Kempson, The MTT Assay: Utility, Limitations, Pitfalls, and Interpretation in Bulk and Single-Cell Analysis, *Int. J. Mol. Sci.,* 2021, **22**, 12827.

9. T-L. Cheung and D. Parker, Temperature dependent luminescence of europium/cyanine FRET pairs, *Chem. Sci.*, 2025, **16**, 19762 – 19768.

10. T-L Cheung, C. Alexander, H. Li and D. Parker, Divergent late-stage functionalisation of luminescent europium(III) complexes for targeting and imaging applications, *Chem. Commun.*, 2025, **61**, 17177 – 17180.

11. X. Xie, J. Fan, M. Liang, Y. Li, X. Jiao, X. Wang and B. Tang, A two-photon excitable and ratiometric fluorogenic nitric oxide photoreleaser and its biological applications, *Chem. Commun.*, 2017, **53**, 11941–11944.
